# Differential transmetallation of complexes of the anti-cancer thiosemicarbazone, Dp4e4mT: effects on anti-proliferative efficacy, redox activity, oxy-myoglobin and oxy-hemoglobin oxidation†

**DOI:** 10.1039/d3sc05723b

**Published:** 2023-12-15

**Authors:** Mahendiran Dharmasivam, Busra Kaya, Tharushi P. Wijesinghe, Vera Richardson, Jeffrey R. Harmer, Miguel A. Gonzalvez, William Lewis, Mahan Gholam Azad, Paul V. Bernhardt, Des R. Richardson

**Affiliations:** a Molecular Pharmacology and Pathology Program, Department of Pathology and Bosch Institute, The University of Sydney Sydney New South Wales 2006 Australia; b Centre for Cancer Cell Biology and Drug Discovery, Griffith Institute for Drug Discovery, Griffith University Nathan Brisbane Queensland 4111 Australia d.richardson@griffith.edu.au m.dharmasivam@griffith.edu.au; c Centre for Advanced Imaging, University of Queensland Brisbane Queensland 4072 Australia; d School of Chemistry and Molecular Biosciences, University of Queensland Brisbane Queensland 4072 Australia; e Department of Chemistry, University of Sydney New South Wales 2006 Australia; f Department of Pathology and Biological Responses, Nagoya University Graduate School of Medicine Nagoya 466-8550 Japan

## Abstract

The di-2-pyridylthiosemicarbazone (DpT) analogs demonstrate potent and selective anti-proliferative activity against human tumors. The current investigation reports the synthesis and chemical and biological characterization of the Fe(iii), Co(iii), Ni(ii), Cu(ii), Zn(ii), Ga(iii), and Pd(ii) complexes of the promising second generation DpT analog, di-2-pyridylketone-4-ethyl-4-methyl-3-thiosemicarbazone (Dp4e4mT). These studies demonstrate that the Dp4e4mT Co(iii), Ni(ii), and Pd(ii) complexes display distinct biological activity *versus* those with Cu(ii), Zn(ii), and Ga(iii) regarding anti-proliferative efficacy against cancer cells and a detrimental off-target effect involving oxidation of oxy-myoglobin (oxy-Mb) and oxy-hemoglobin (oxy-Hb). With regards to anti-proliferative activity, the Zn(ii) and Ga(iii) Dp4e4mT complexes demonstrate facile transmetallation with Cu(ii), resulting in efficacy against tumor cells that is strikingly similar to the Dp4e4mT Cu(ii) complex (IC_50_: 0.003–0.006 μM and 72 h). Relative to the Zn(ii) and Ga(iii) Dp4e4mT complexes, the Dp4e4mT Ni(ii) complex demonstrates kinetically slow transmetallation with Cu(ii) and intermediate anti-proliferative effects (IC_50_: 0.018–0.076 μM after 72 h). In contrast, the Co(iii) and Pd(ii) complexes demonstrate poor anti-proliferative activity (IC_50_: 0.262–1.570 μM after 72 h), probably due to a lack of transmetallation with Cu(ii). The poor efficacy of the Dp4e4mT Co(iii), Ni(ii), and Pd(ii) complexes to transmetallate with Fe(iii) markedly suppresses the oxidation of oxy-Mb and oxy-Hb. In contrast, the 2 : 1 Dp4e4mT: Cu(ii), Zn(ii), and Ga(iii) complexes demonstrate facile reactions with Fe(iii), leading to the redox active Dp4e4mT Fe(iii) complex and oxy-Mb and oxy-Hb oxidation. This study demonstrates the key role of differential transmetallation of Dp4e4mT complexes that has therapeutic ramifications for their use as anti-cancer agents.

## Abstract 2

The differential transmetallation of Dp4e4mT complexes provides valuable insights into their biological mechanism of action, off-target effects, and their potential therapeutic applications.

## Graphical abstract

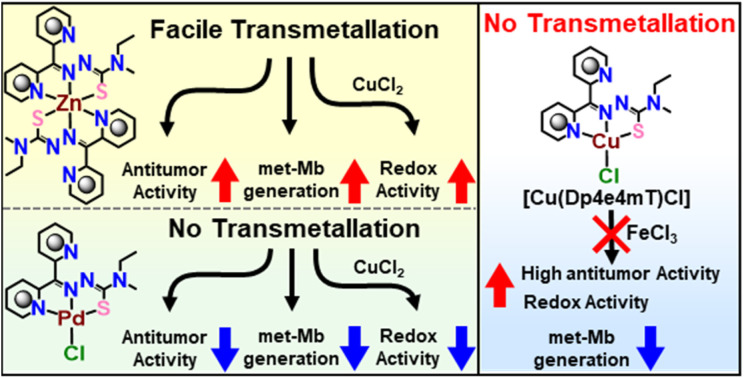

## Introduction

**Fig. 1:**
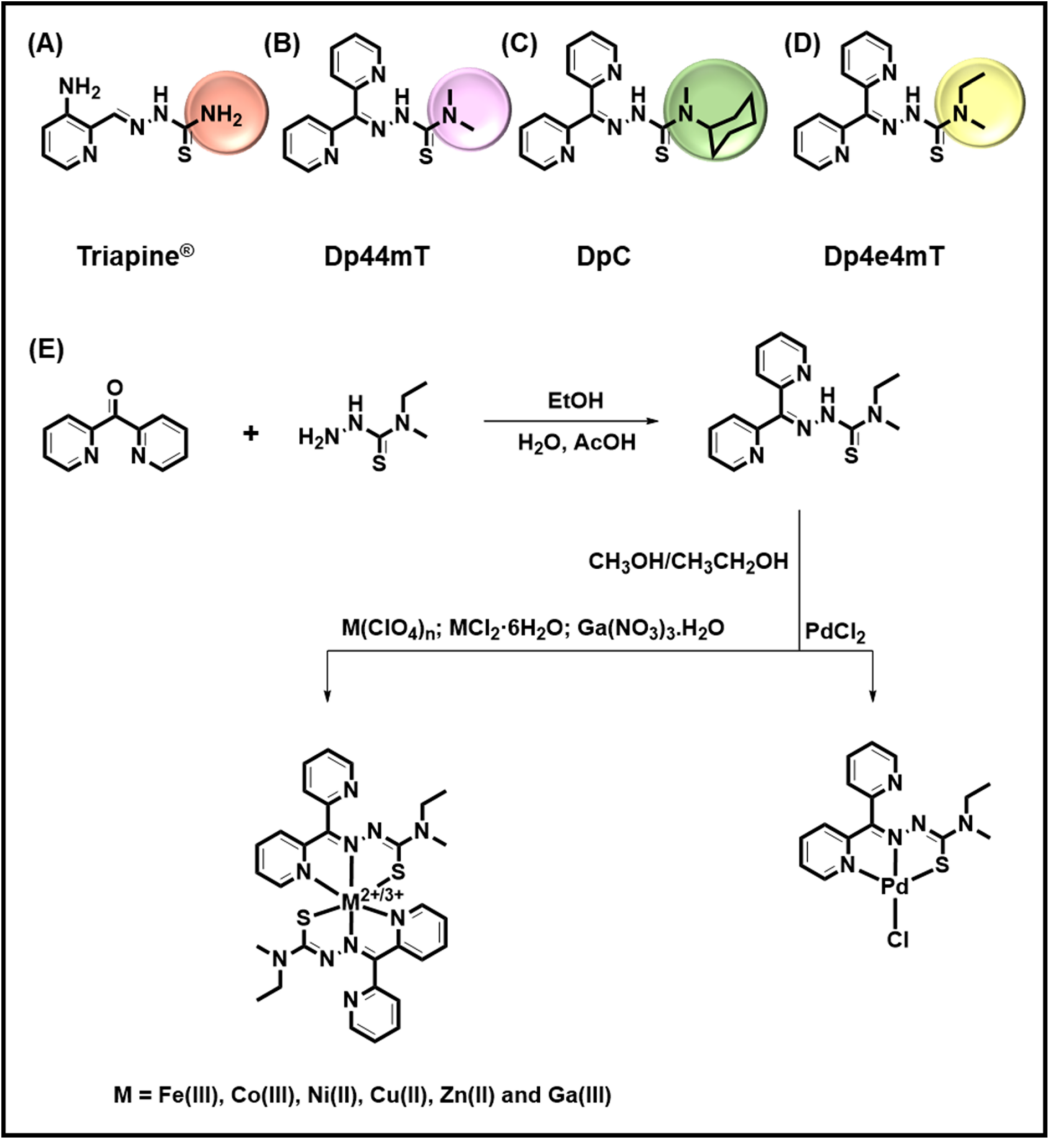
Line drawings of the structures of: (A) Triapine®; (B) Dp44mT; (C) DpC; and (D) Dp4e4mT. (E) Scheme demonstrating the synthesis of: Dp4e4mT (EtOH/H_2_O, AcOH; reflux for 2 h) and its Fe(iii), Co(iii), Ni(ii), Cu(ii), Zn(ii), Ga(iii) and Pd(ii) complexes (Fe(ClO_4_)_3_·6H_2_O, CoCl_2_·6H_2_O, NiCl_2_·6H_2_O, Cu(ClO_4_)_2_·6H_2_O, Zn(ClO_4_)_2_·6H_2_O Ga(NO_3_)_3_·H_2_O and PdCl_2_, CH_3_OH/CH_3_CH_2_OH; reflux, 2 h).

Thiosemicarbazones are a class of ligands clinically investigated for a variety of biological activities, such as anti-microbial, anti-protozoal, anti-viral, anti-fungal, anti-malarial, anti-oxidant, and anti-tumor activities.^1–4^ This research has demonstrated that N-heterocyclic thiosemicarbazones that utilize the N,N,S donor system^5^ in the formation of Cu(ii) and Zn(ii) complexes have increased anti-proliferative activity relative to their ligands both *in vitro*^6,7^ and *in vivo*.^8^ Interestingly, 3-aminopyridine-2-carboxaldehyde thiosemicarbazone (Triapine®; Fig. 1A) was tested as a potent anti-cancer agent against prostate, pancreas, lung, kidney and ovary cancers and has undergone over 20 clinical trials.^9–11^ However, Triapine® demonstrated less than optimal anti-tumor activity against some tumor-types and its dose-limiting side effects such as met-hemoglobinemia and hypoxia have seriously limited its clinical potential.^9,12^

Our laboratory pioneered the di-2-pyridylketone thiosemicarbazone class of ligands (DpT; Fig. 1) that show pronounced and selective anti-tumor activity against a variety of cancers *in vivo* and demonstrate the ability to overcome drug resistance *via* a number of mechanisms.^13–19^ This marked and safe anti-tumor activity was confirmed by multiple other groups *in vitro* and *in vivo*.^20–22^ A key part of their activity involved the “double punch” mechanism^23,24^ whereby these agents bind iron and particularly copper that are necessary for cellular proliferation (the first punch). Upon the formation of these complexes intracellularly, and particularly in lysosomes,^25,26^ these agents redox cycle to generate reactive oxygen species (ROS).^23,27^ This generation of ROS then leads to lysosomal membrane permeabilization and mitochondrial apoptosis that constitutes the “second punch”.^25^ As part of this cytotoxic mechanism, transmetallation between Zn(ii) thiosemicarbazone complexes and Cu (leading to the potently cytotoxic Cu complex) occurs in lysosomes, and plays an important role in their redox and anti-proliferative activity.^26,28–31^ Extracellular chelation of metals by thiosemicarbazones can also occur, with albumin being reported to act as a source of Cu.^32^ Our studies showed that di-2-pyridylketone 4,4-dimethyl-3-thiosemicarbazone (Dp44mT; 0.4 mg per kg per day; Fig. 1B) selectively inhibited a variety of human tumor xenografts in nude mice.^13–18,33^ Dp44mT was more effective than much higher doses of the clinically trialed thiosemicarbazone, Triapine® (12 mg per kg per day), and showed less toxicity.^16^ Unfortunately, mice administered intravenously with intensive non-optimal doses of Dp44mT demonstrated cardiac fibrosis,^16^ although the mechanism involved remained unclear.

Considering the limitations of the first generation of DpT analogs, a second generation was prepared.^13,15^ One of the best of these ligands was di-2-pyridylketone 4-cyclohexyl-4-methyl-3-thiosemicarbazone (DpC; Fig. 1C),^13,15^ which demonstrated: (1) high tolerability; (2) potent anti-cancer activity; (3) synergistic efficacy with multiple chemotherapies; and (4) the ability to overcome tamoxifen resistance and P-glycoprotein-mediated resistance.^13,15,17–19,34–36^ Due to its optimal properties, DpC entered clinical trials for advanced and resistant cancers in 2016 (NCT02688101), again highlighting its selectivity and tolerability.^37^

Studies from our laboratories *in vitro* and *in vivo* also demonstrated the Fe(iii) complexes of Dp44mT and DpC induced detrimental oxidation of the heme moiety in oxy-hemoglobin (oxy-Hb) and oxy-myoglobin (oxy-Mb) to met-Hb and met-Mb, respectively.^38,39^ Importantly, the DpC Fe(iii) complex was far less active than the Dp44mT Fe(iii) complex at oxidizing these latter proteins.^38,39^ Anecdotal reports from the clinical trial with DpC suggested muscle pain (myalgia) in patients, although the mechanism involved remained unclear.^40,41^ Considering the high levels of Mb in muscles and its key role in oxygen storage and transport, it was hypothesized that DpC may have resulted in some oxidation of oxy-Mb to met-Mb.^28,42^ Similarly, the cardiac toxicity of Dp44mT leading to fibrosis may potentially be related to its ability to oxidize oxy-Mb, disturbing essential oxygen metabolism in cardiomyocytes.

Apart from DpC, the analysis of the second generation DpT analogs also identified di-2-pyridylketone 4-ethyl-4-methyl-3-thiosemicarbazone (Dp4e4mT; Fig. 1D) as an agent with considerable promise in terms of its potency and selectivity against tumor cells *in vitro* and *in vivo*.^15^ In fact, the beneficial properties of Dp4e4mT included: (1) similar or greater anti-proliferative efficacy than DpC *in vitro*; (2) Dp4e4mT was more effective than DpC in reducing uptake of the essential metal, iron, from the iron transport protein, transferrin, and increasing iron release from tumor cells; (3) similarly to DpC, Dp4e4mT demonstrated potent efficacy against a lung tumor xenograft and tolerability *in vivo* after oral administration;^15^ (4) Dp4e4mT, like DpC, did not induce cardiac fibrosis in mice;^15^ and (5) Dp4e4mT and its 1 : 1 Cu(ii) complex demonstrated synergy when combined with the cytotoxic agents, gemcitabine or cisplatin.^15^

Considering the marked potential of Dp4e4mT as an anti-cancer agent, the current investigation examined for the first time the influence of its complexation with Fe(iii), Co(iii), Ni(ii), Cu(ii), Zn(ii), Ga(iii), and Pd(ii) to examine the effect on anti-proliferative activity and the oxidation of oxy-Hb and oxy-Mb. Apart from complexation with Cu(ii),^15^ the biological effects of complexation of Dp4e4mT with a variety of metal ions has not been examined. Previous studies with related aroylhydrazone ligands indicated that complexation with Ga(iii) could promote anti-proliferative activity,^43^ while complexation of Dp44mT and other thiosemicarbazones with Zn(ii) or Cu(ii) could suppress or totally prevent oxidation of oxy-Mb and oxy-Hb.^28,39^ As such, the role of transmetallation in the biological activity of thiosemicarbazones is critical to evaluate. This is particularly important considering that the Cu complexes play key roles in the anti-proliferative activity of this class of thiosemicarbazones.^26,28–31^ Hence, the ability of a complex to undergo facile transmetallation to its Cu species will probably dictate its potential as a clinically useful anti-tumor drug.

Herein, we report the synthesis and chemical and biological characterization of the complexes of Dp4e4mT with Fe(iii), Co(iii), Ni(ii), Cu(ii), Zn(ii), Ga(iii), and Pd(ii), and demonstrate that the Co(iii), Ni(ii), and Pd(ii) complexes display distinct biological activity to the complexes with Zn(ii), Ga(iii), and Cu(ii). This is related, in part, to the differential transmetallation activity of these two sets of complexes. In fact, Co(iii), Ni(ii), and Pd(ii) complexes demonstrate an inability to transmetallate with Fe(iii), leading to favorably low activity at oxidizing oxy-Mb and oxy-Hb. However, unlike the Co(iii) and Pd(ii) complexes, the Ni(ii) complex transmetallates with Cu(ii), leading to a redox active complex. The ability of Cu(ii) to transmetallate with the Zn(ii), Ga(iii), and to a lesser extent, Ni(ii) complexes of Dp4e4mT, was correlated with higher anti-proliferative efficacy. Understanding these transmetallation properties is important in the clinical application of these or future thiosemicarbazones and their complexes as effective anti-cancer agents.

## Results and discussion

### Synthesis of Dp4e4mT and its Fe(iii), Co(iii), Ni(ii), Cu(ii), Zn(ii), Ga(iii), and Pd(ii) complexes

Dp4e4mT was prepared by using our previously reported synthetic procedure.^15^ The complexes were obtained by reaction with salts of two- or three-valanced metal cations (M^2+/3+^; Fe(ClO_4_)_3_·6H_2_O, CoCl_2_·6H_2_O, NiCl_2_·6H_2_O, Cu(ClO_4_)_2_·6H_2_O, Zn(ClO_4_)_2_·6H_2_O Ga(NO_3_)_3_·H_2_O, and PdCl_2_) in alcoholic solvent under reflux. Fig. 1E presents a scheme for the synthetic route for Dp4e4mT and its complexes. Dp4e4mT and its complexes were characterized by ^1^H-NMR, mass spectrometry, UV-vis spectroscopy, elemental analysis, cyclic voltammetry, and single crystal X-ray diffraction analysis.

#### Crystallography

**Fig. 2:**
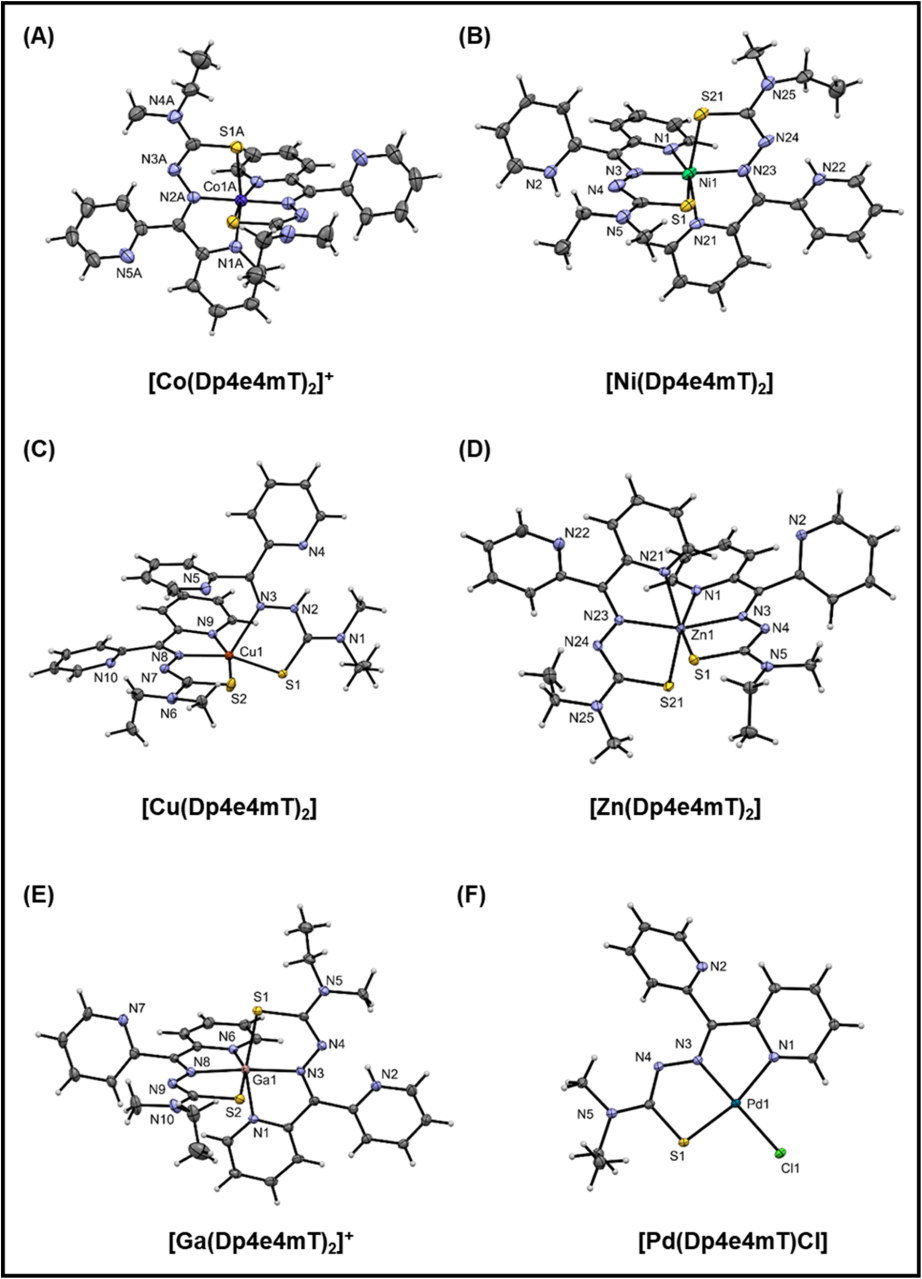
(A–F) Crystal structures of the Co(iii), Ni(ii), Cu(ii), Zn(ii), Ga(iii), and Pd(ii) complexes of Dp4e4mT. Thermal ellipsoids shown at the 30% probability level.

The X-ray crystal structures (Fig. 2A–F) and crystal packing diagrams (Fig. S1A–F†) of the Co(iii), Ni(ii), Cu(ii), Zn(ii), Ga(iii), and Pd(ii) complexes of Dp4e4mT were part of the chemical characterization, but also provide important information relevant to understanding their biological activity. For the Co(iii), Ni(ii), Zn(ii), and Ga(iii) complexes (Fig. 2A, B, D and E), the metal is coordinated to two meridionally arranged ligands in a distorted octahedral geometry. All metal ions are coordinated *via* one of the two pyridine groups, the imine nitrogen, and the sulfur atom of each ligand.

In the structure of [Co(Dp4e4mT)_2_]^+^ (Fig. 2A), there are two independent complex cations each situated on a 2-fold axis. The bond lengths are as expected for a low spin d^6^ complex with this ligand system.^44^ The *trans* N–Co–N (imine) angles are essentially linear (179.4(3)° and 178.3(3)°), and the *trans* N–Co–S angles are *ca.* 169°. These angles are a useful indicator of distortion of the complex from octahedral symmetry. The five-membered chelate ring angles are contracted from their ideal values to *ca.* 80°, as expected.

The structure of [Ni(Dp4e4mT)_2_] (Fig. 2B), finds the non-coordinating pyridine nitrogens protonated to give a di-cationic complex. There is H-bonding involving the water molecule and the chloride anions. The Ni–N and Ni–S bond lengths are consistent with structural analogs (*e.g.*, neutral [Ni(Dp4pT)_2_])^45^ showing that protonation of the pyridyl N-atoms has little effect on the coordination sphere. The *trans* N–Ni–N (imine) angle is 176.0(4)° that is consistent with the electronic preference of the high spin d^8^ complex for octahedral symmetry. Further, the *trans* N–Ni–S angles contract to *ca.* 160° due to the longer Ni(ii) coordinate bonds relative to Co(iii), which tighten the bite angles across the tridentate ligand.

The structure of [Cu(Dp4e4mT)_2_] is unusual (Fig. 2C), with one ligand binding in a tridentate N, N, S mode, while the other binds in a bidentate N, S fashion *via* the imine and S-donor. This bidentate coordinated ligand is protonated in the 3-postion (N2 in Fig. 2C). A water molecule is H-bonded to a non-coordinated pyridine and also the perchlorate anion (N2–H2–N4; Fig. S1C†). In the d^9^ electronic ground state, the Jahn–Teller effect results in an extension of the coordinate bonds along one axis of an ideal 6-coordinate complex, as we previously reported in the structure of [Cu(Dp44mT)_2_].^27^ In the current structure (Fig. 2C), the axial distortion is so great that the pyridyl ligand dissociates, completely leaving an elongated bond to the imine donor N3 (2.315(2) Å) compared with the equatorially coordinated N8 (1.988(2) Å). The closely related benzoylpyridine thiosemicarbazone complex [Cu(Bp44mT)_2_], also reported by our laboratories,^46^ shows a similar 5-coordinate geometry with one pyridyl donor dissociated.

Further, the Cu–S bonds in [Cu(Dp4e4mT)_2_] are appreciably different lengths (*i.e.*, Cu–S2 2.2630(7) and Cu–S1 2.3195(6) Å), which can be attributed to ligand protonation (Fig. 2C). The HN–C=S group (including S1) present as a thioamide tautomer has lowered donor capacity relative to the deprotonated ligand (including S2), where the imine-thiolate (N=C–S^−^) form is present. The geometry of the ensuing five-coordinate complex is distorted square pyramidal (*τ* = 0.04: *τ* = (*α* − *β*)/60), where *α* and *β* are the two largest coordinate angles. The consequences of this electronically driven distortion are appreciable, and in solution, only one ligand remains coordinated. In fact, this dissociation has biological consequences in terms of redox chemistry and the oxidation of heme in oxy-Mb and oxy-Hb, and this will be discussed further below.

In the neutral [Zn(Dp4e4mT)_2_] complex (Fig. 2D), the coordinate bond lengths are as expected,^45^ and the *trans* N–Zn–N (imine) (160.22(7)°) and *trans* N–Zn–S (*ca.* 154°) coordinate angles deviate markedly from their ideal octahedral values. This is observed as the d^10^ Zn(ii) atom has no electronic preference for octahedral geometry. The structure of [Ga(Dp4e4mT)(HDp4e4mT)](NO_3_)_2_ · 2¾H_2_O crystallized with two molecules in the asymmetric unit (Fig. S1E†). Both thioamide moieties are deprotonated, while one of the two non-coordinating pyridines is protonated. The charge balance is completed by two nitrate anions. The coordinate bond lengths in [Ga(Dp4e4mT)_2_]^+^ are similar to those found in other thiosemicarbazone complexes.^47^ The coordination geometry of [Ga(Dp4e4mT)_2_]^+^ is essentially the same as [Ni(Dp4e4mT)_2_]^2+^, with *trans* coordinate angles N–Ga–N (imine) (173.2(2)/175.5(2)) and *trans* N–Ga–S angles of *ca.* 160° (*cf.*Fig. 2B and E). In this case, the enhanced ionic character of the coordinate bonds to the Ga(iii) ion (relative to Ni(ii)) compensates for the loss of ligand field stabilization energy (d^10^ for Ga(iii) and d^8^ for Ni(ii)).

In accord with its low spin d^8^ ground state, [Pd(Dp4e4mT)Cl] adopts a distorted square planar geometry (Fig. 2F) comprising one pyridine group, the imine nitrogen, the sulfur atom, and a chlorido co-ligand. The non-coordinating pyridine ring participates in π–π interactions with adjacent molecules. The coordinate bonds and angles are consistent with structural analogs.^48^

### Solution tramsmetallation studies

**Fig. 3:**
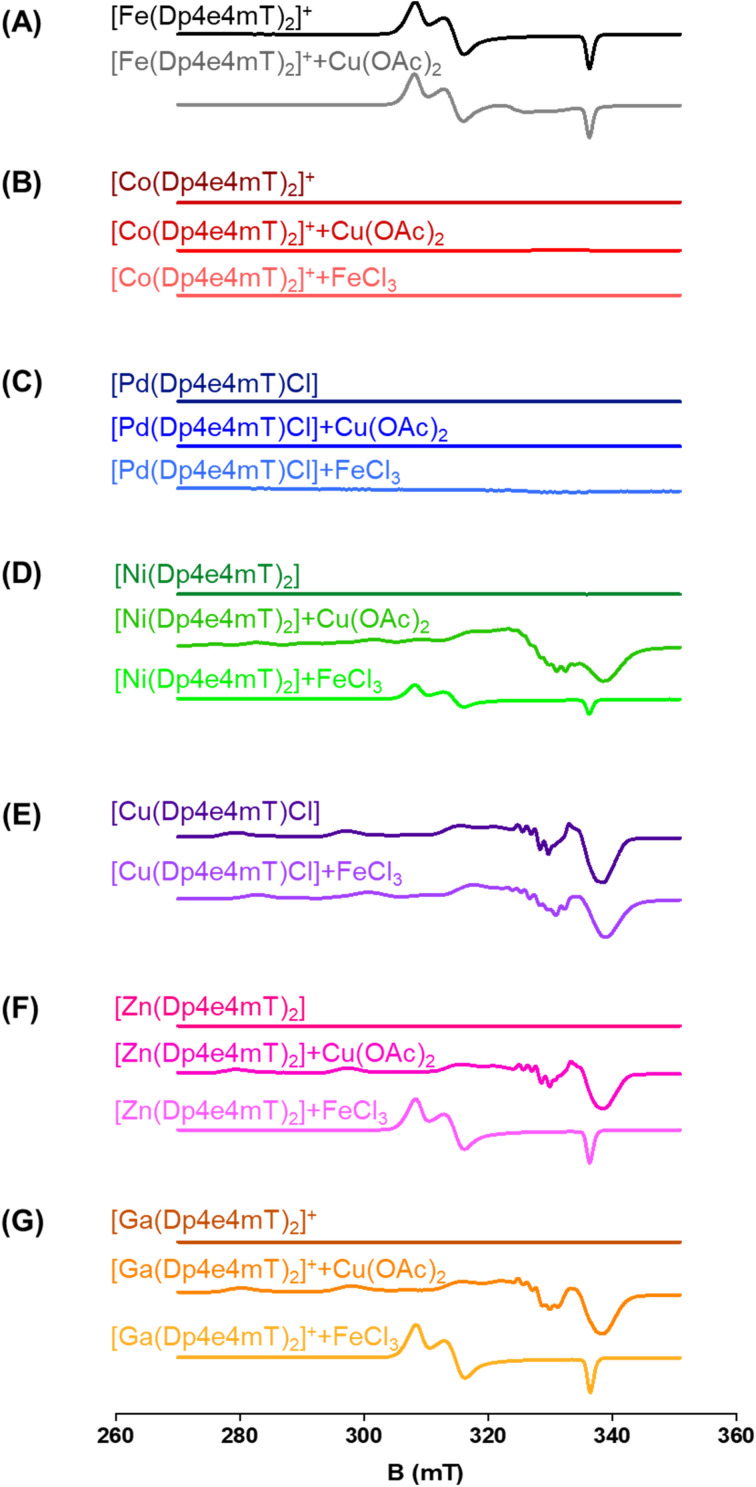
(A–G) The transmetallation of the Dp4e4mT complexes was examined using X-band CW EPR spectroscopy. The complexes (500 μM in DMSO : CHCl_3_ 1 : 1) and their products after reaction with equimolar amounts of Cu(OAc)_2_ and FeCl_3_ after a 24 h/20 °C incubation.

The capacity of the Fe(iii), Ni(ii), Co(iii), Zn(ii), Ga(iii), and Pd(ii) complexes of Dp4e4mT to transmetallate with Cu(ii) and/or Fe(iii) to result in redox active Cu(ii) and Fe(iii) complexes could be critical in understanding their anti-proliferative efficacy, redox activity, and ability to oxidize oxy-Mb and oxy-Hb. To examine this, LC-MS spectrometry was performed in DMSO after the addition of 1 equivalent of FeCl_3_ or Cu(OAc)_2_ and the solutions then incubated for 24 h/20 °C due to slow reaction kinetics. This solution was then diluted 1 : 1 in methanol and LC-MS performed using a mobile phase consisting of 90% MeOH, 9.9% water, and 0.1% formic acid (Fig. S2–S9†). These investigations were supported by EPR studies in DMSO after the addition of 1 equivalent of FeCl_3_ or Cu(OAc)_2_ incubated under the same conditions (Fig. 3A–G). As a third method to investigate transmetallation, kinetic studies using time-resolved UV-vis spectroscopy were performed under pseudo-first order conditions with a 10-fold excess of FeCl_3_ or Cu(OAc)_2_ in DMSO (Fig. S11–S16†). The pseudo-first order rate constants were calculated from the time-dependent UV-vis spectroscopy data (at all wavelengths) with Reactlab Kinetics^49^ and collected in Table S2.†

#### [Fe(Dp4e4mT)_2_]^+^

Upon adding 1 equivalent of Cu(OAc)_2_, the LC-MS analysis of [Fe(Dp4e4mT)_2_]^+^ (*m*/*z*: 652.2; Fig. S2A and B†) demonstrated little transmetallation in DMSO to [Cu(Dp4e4mT)]^+^, [Cu(Dp4e4mT)MeOH]^+^, and [Cu(Dp4e4mT)DMSO]^+^ (*m*/*z*: 361.1, 393.1, and 439.1, respectively; Fig. S2B†). This latter mixture of Cu(ii) complexes is referred to hereon as [Cu(Dp4e4mT)X]. A peak at *m*/*z*: 767.1 is also observed and is assigned to dimer formation (*i.e.*, 2× [Cu(Dp4e4mT)] with formate ion from the LC-MS solvent system; Fig. S2B†).

To further examine this transmetallation reaction using EPR, addition of Cu(OAc)_2_ to [Fe(Dp4e4mT)_2_]^+^ led to minor changes in its spectrum (Fig. 3A). A signal at ∼330 mT may be due to the small amount of [Cu(Dp4e4mT)(DMSO)]^+^(Fig. 3A), which supports the LC-MS data (Fig. S2B†). The EPR spectra of [Fe(Dp4e4mT)_2_]^+^ (Fig. 3A and S10A†) is typical of a six-coordinate low spin ferric complex with rhombic symmetry. The EPR signal of [Fe(Dp4e4mT)_2_]^+^ is similar to that recently reported for the Fe(iii) complexes of the closely related phenyl-1-(2-pyridinyl)-2-propen-1-one-4-phenylthiosemicarbazone (PPP4pT) ligands.^42^ No signals from free Cu(ii) were observed in the EPR spectra due to the choice of Cu(OAc)_2_, which is dominantly present (in DMSO) as its dimeric form, where it possesses a spin triplet ground state.^50^

UV-vis spectroscopy demonstrated that the reaction of [Fe(Dp4e4mT)_2_]^+^ with Cu(OAc)_2_ was rather unusual (Fig. S11A and Table S2†). Upon mixing, a peak at 550 nm immediately formed and then diminished in intensity. This reaction occurred in two consecutive first order steps (*t*_½_ ∼6 min and ∼1 h) to give a spectrum indistinguishable from the starting complex [Fe(Dp4e4mT)_2_]^+^. The origin of the intermediate species is unknown, but may correspond to [Cu(Dp4e4mT)DMSO]^+^ observed in LC-MS (Fig. S2B†).

#### [Co(Dp4e4mT)_2_]^+^ and [Pd(Dp4e4mT)Cl]

Upon analyzing the LC-MS results, it was evident that upon the addition of 1 equivalent of FeCl_3_ or Cu(OAc)_2_, the spectra of [Co(Dp4e4mT)_2_]^+^ (*m*/*z*: 655.2; Fig. S3A–C†) and [Pd(Dp4e4mT)Cl] (*m*/*z*: 404.02; Fig. S4A–C†) remained unaltered. These findings were further confirmed by EPR, with no evidence of transmetallation when [Co(Dp4e4mT)_2_]^+^ and [Pd(Dp4e4mT)Cl] were reacted with FeCl_3_ or Cu(OAc)_2_ (Fig. 3B and C). Of interest, [Co(Dp4e4mT)_2_]^+^ and [Pd(Dp4e4mT)Cl] are diamagnetic and EPR silent, with only weak EPR signals from high spin (unreacted) Fe(iii) being apparent at low field (100–200 mT; data not shown). UV-vis spectroscopy demonstrated that [Co(Dp4e4mT)_2_]^+^ (Fig. S12A and B†) and [Pd(Dp4e4mT)Cl] (Fig. S13A and B†) exhibited no significant changes to their spectra upon the addition of either Cu(OAc)_2_ or FeCl_3_ over timescales of more than 12 h. Hence, it can be concluded that [Co(Dp4e4mT)_2_]^+^ and [Pd(Dp4e4mT)Cl] are substitution-inert, with no transmetallation occurring in DMSO (Fig. S12A, B, S13A and B†).

#### [Ni(Dp4e4mT)_2_]

Examining LC-MS, upon adding 1 equivalent of FeCl_3_, [Ni(Dp4e4mT)_2_] exhibits a mixture of [Fe(Dp4e4mT)_2_]^+^ (*m*/*z*: 652.2) and *N*-ethyl-*N*-methyl-1-(pyridine-2-yl)-3, 9a-dihydro-4*H*-pyrido[1,2-*d*][1, 2, 4] triazin-4-amine-nickel (termed [Ni(Dp4e4mT-S)]; *m*/*z*: 328.10; Fig. S5A and B†). These observations suggest incomplete transmetallation of [Ni(Dp4e4mT)_2_] to [Fe(Dp4e4mT)_2_]^+^ (Fig. S5A†). The species, [Ni(Dp4e4mT-S)], is suggested based on reports of transition metal-triggered partial desulfuration and intra-molecular cyclization in multiple thiosemicarbazone systems.^51–56^ Interestingly, [Ni(Dp4e4mT-S)] (*m*/*z*: 328.0) was also observed in the spectrum of [Ni(Dp4e4mT)_2_] itself (Fig. S5B†). This finding suggests [Ni(Dp4e4mT)_2_] dissociates into a mixture of the 2 : 1 and 1 : 1 L : Ni complexes in DMSO and that upon the addition of FeCl_3_, the stable 1 : 1 complex does not undergo transmetallation (Fig. S5A and B†).

In contrast, upon the addition of Cu(OAc)_2_, [Ni(Dp4e4mT)_2_] completely transmetallates into [Cu(Dp4e4mT)X] (Fig. S5B and C†). Again, the peak at *m*/*z*: 769.2 is attributed to dimers of [Cu(Dp4e4mT)] (*i.e.*, 2× [Cu(Dp4e4mT)] with formate ion). Of note, in contrast to the addition of FeCl_3_ to [Ni(Dp4e4mT)_2_] (Fig. S5A and B†), [Ni(Dp4e4mT-S)] (*m*/*z*: 328.0) was not evident upon the addition of Cu(OAc)_2_ to [Ni(Dp4e4mT)_2_] (Fig. S5B and C†), again supporting complete transmetallation.

Similar observations were also noted using EPR spectroscopy. A partial reaction of FeCl_3_ with [Ni(Dp4e4mT)_2_] occurred with the relatively weak signal of [Fe(Dp4e4mT)_2_]^+^ being evident (Fig. 3D). In fact, this latter [Fe(Dp4e4mT)_2_]^+^ signal was *ca.* 30% the intensity observed with the Dp4e4mT Zn(ii) and Ga(iii) complexes that underwent complete transmetallation with FeCl_3_ (*cf.*Fig. 3D with F and G). EPR also provided strong support for the transmetallation of [Ni(Dp4e4mT)_2_] upon the addition of Cu(OAc)_2_ (Fig. 3D), with the resultant spectrum being superimposable with [Cu(Dp4e4mT)(OAc)]. A second, unidentified Cu(ii) complex is also present after transmetallation of [Ni(Dp4e4mT)_2_] with Cu(OAc)_2_ (Fig. 3D) that may correspond to [Cu(Dp4e4mT)(DMSO)]^+^ observed using LC-MS (Fig. S5B and C†). Of note, d^8^ [Ni(Dp4e4mT)_2_] is high spin, paramagnetic, and thus, EPR silent due to a large zero-field splitting (Fig. 3D). Further evidence of transmetallation was provided by UV-vis spectroscopy, where the reaction of [Ni(Dp4e4mT)_2_] with Cu(OAc)_2_ was appreciable (Fig. S14A†) and much faster (*t*_½_ ∼2 h) than with FeCl_3_, which was unreactive (Fig. S14B and Table S2†).

#### [Cu(Dp4e4mT)_2_]

In DMSO, LC-MS showed that [Cu(Dp4e4mT)_2_] (*m*/*z*: 660.2) dissociates into [Cu(Dp4e4mT)X]^+^, and liberates the ligand *m*/*z*: 300.2 (Fig. S6A†). The 767.1 peak is attributed to the dimer of [Cu(Dp4e4mT)]^+^ with formate ion from the LC-MS solvent system (Fig. S6A†). Upon the addition of 1 equivalent of FeCl_3_, the liberated ligand becomes coordinated to Fe(iii) to form [Fe(Dp4e4mT)_2_]^+^ (*m*/*z*: 652.2; Fig. S6B†), while the stable [Cu(Dp4e4mT)X]^+^ does not undergo transmetallation (Fig. S6B†).

#### [Cu(Dp4e4mT)Cl]

As expected, the 1 : 1 Cu : L complex (*i.e.*, [Cu(Dp4e4mT)Cl]) is stable and not involved in transmetallation after the addition of FeCl_3,_ with [Cu(Dp4e4mT)X]^+^ being evident (Fig. S7A and B†). Again, the peak at *m*/*z*: 767.1 is suggested to be the [Cu(Dp4e4mT)] dimer with formate ion (Fig. S7A and B†). Similarly, examination of the EPR spectrum of [Cu(Dp4e4mT)Cl] after addition of FeCl_3_ revealed no evidence of [Fe(Dp4e4mT)_2_]^+^, although more than one Cu(ii) signal was observed (Fig. 3E). These later EPR signals may represent a mixture of [Cu(Dp4e4mT)Cl] and [Cu(Dp4e4mT)(DMSO)]^+^, which is consistent with the LC-MS data (Fig. S7B†). The EPR spectrum of [Cu(Dp4e4mT)Cl] is typical of an essentially square planar complex of the tridentate thiosemicarbazone with a chlorido co-ligand (Fig. S10B†).^27,42^ UV-vis spectroscopy demonstrated the reaction of [Cu(Dp4e4mT)Cl] with FeCl_3_ showed no evidence of transmetallation (Fig. S11B†), as demonstrated by LC-MS (Fig. S7B†) and EPR (Fig. 3E).

#### [Zn(Dp4e4mT)_2_] and [Ga(Dp4e4mT)_2_]^+^

Significant LC-MS alterations were observed upon the addition of FeCl_3_ or Cu(OAc)_2_ to [Zn(Dp4e4mT)_2_] (Fig. S8A–C†) or [Ga(Dp4e4mT)_2_]^+^ (Fig. S9A–C†). Upon the addition of FeCl_3_ or Cu(OAc)_2_, facile transmetallation of [Zn(Dp4e4mT)_2_] (*m*/*z*: 661.4) was observed to either [Fe(Dp4e4mT)_2_]^+^ (*m*/*z*: 652.2; Fig. S8A and B†) or [Cu(Dp4e4mT)_2_] (*m*/*z*: 660.2; Fig. S8B and C†), respectively. Similarly, LC-MS demonstrated that [Ga(Dp4e4mT)_2_]^+^ (*m*/*z*: 665.2) underwent facile transmetallation to [Fe(Dp4e4mT)_2_]^+^ (*m*/*z*: 652.4) upon titration of FeCl_3_ (Fig. S9A and B†) or formed [Cu(Dp4e4mT)X] upon adding Cu(OAc)_2_ (Fig. S9B and C†). This observation was further confirmed by EPR spectroscopy, where upon the addition of FeCl_3_ or Cu(OAc)_2_, both [Zn(Dp4e4mT)_2_] and [Ga(Dp4e4mT)_2_]^+^ reacted to yield [Fe(Dp4e4mT)_2_]^+^ or [Cu(Dp4e4mT)(OAc)], respectively (Fig. 3F and G). Both [Zn(Dp4e4mT)_2_] and [Ga(Dp4e4mT)_2_]^+^ are diamagnetic, and thus, EPR silent. The UV-vis spectroscopy demonstrated the reaction between [Zn(Dp4e4mT)_2_] and Cu(OAc)_2_ was rapid (Table S2†), with complete transmetallation to [Cu(Dp4e4mT)(OAc)] occurring during mixing time (Fig. S15A†). When [Zn(Dp4e4mT)_2_] was reacted with FeCl_3_ (Fig. S15B†), a slower and measurable spectral change was observed (*k*_obs_ 8.91 × 10^−3^ s^−1^, *t*_½_ ∼1 min; Table S2†). The transmetallation reactions of [Ga(Dp4e4mT)_2_]^+^ with Cu(OAc)_2_ (Fig. S16A†) and FeCl_3_ (Fig. S16B†) were facile as determined by UV-vis spectroscopy, and occurred at similar rates (*t*_½_ ∼3–4 h) to afford [Cu(Dp4e4mT)(OAc)] and [Fe(Dp4e4mT)_2_]^+^, respectively.

Collectively, the combined data (in DMSO) from LC-MS (Fig. S2–S9†), EPR (Fig. 3A–G), and time-resolved UV-vis spectroscopy (Fig. S11–S16†), indicates that [Zn(Dp4e4mT)_2_] undergoes facile transmetallation upon the addition of Cu(ii) and Fe(iii) (affording [Cu(Dp4e4mT)Cl] and [Fe(Dp4e4mT)_2_]^+^) on timescales of seconds to minutes (Table S2†). The [Ga(Dp4e4mT)_2_]^+^ complex is also susceptible to transmetallation by Cu(ii) and Fe(iii), but on a timescale of several hours. The [Ni(Dp4e4mT)_2_] complex reacts very sluggishly with Fe(iii) (the reaction being only ∼20% complete even after 3 days), but its reaction with Cu(ii) is markedly faster and complete within a few hours (Table S2†). The [Co(Dp4e4mT)_2_]^+^ and [Pd(Dp4e4mT)Cl] complexes exhibited no reactivity with either Cu(OAc)_2_ or FeCl_3_, and as such, these complexes are substitution-inert.

### Transmetallation in aqueous solutions using UV-vis spectroscopy

Transmetallation studies were also conducted using UV-vis spectroscopy in aqueous solutions at pH 7.4 (phosphate buffer; 100 mM) and pH 5 (acetate buffer; 150 mM) to simulate physiological conditions, as thiosemicarbazones of this class transverse the cytosol (pH 7.4) to target lysosomes (pH 5), where transmetallation occurs.^25,26,28^ Studies in aqueous solution were important to compare the results performed in DMSO above so as to interpret the biological studies and particularly the redox assays below where the same pH values were employed.

Transmetallation of the complexes of Dp4e4mT with Cu(OAc)_2_ in water was possible as it is soluble in aqueous solution at pH 7.4 and 5, while FeCl_3_ precipitates under these conditions and cannot be assessed. Kinetic data in aqueous solution were consistent with the DMSO results, although some differences in reactivity were demonstrated between these solvent systems (Table S2†). The UV-vis spectra of all complexes in water (*ca*. 10 μM solutions) are compared in Fig. S17† and mirror the spectra in DMSO (see Experimental section). The spectra of [Fe(Dp4e4mT)_2_]^+^, [Co(Dp4e4mT)_2_]^+^, and [Pd(Dp4e4mT)Cl] are similar, and display an asymmetric maximum around 393 nm with a prominent shoulder (∼450 nm). The [Ni(Dp4e4mT)_2_] spectrum is similarly asymmetric, although the peak intensities are reversed relative to the Fe(iii), Co(iii), and Pd(ii) spectra (Fig. S17†). Both [Cu(Dp4e4mT)(OH_2_)]^+^ and [Ga(Dp4e4mT)_2_]^+^ exhibit quite symmetrical maxima at a wavelength of ∼415 nm. The spectrum of [Zn(Dp4e4mT)_2_] is unique in this group, with a symmetrical peak at ∼400 nm.

The reaction of Cu(OAc)_2_ with [Fe(Dp4e4mT)_2_]^+^ (at pH 7.4 and 5.0) led to an immediate prominent shoulder at 500 nm (Fig. S18A and B†), which slowly subsided (*k*_obs_ 7.3 × 10^−4^ s^−1^, *t*_½_ ∼15 min), while no marked change to the *λ*_max_ of [Fe(Dp4e4mT)_2_]^+^ at 393 nm was observed. These spectral changes suggest a transitory alteration in the structure of [Fe(Dp4e4mT)_2_]^+^ upon the addition Cu(OAc)_2_, although the precise species formed is unclear. Of note, LC-MS (Fig. S2B†) and EPR (Fig. 3A) demonstrated limited transmetallation of [Fe(Dp4e4mT)_2_]^+^ to [Cu(Dp4e4mT)X]^+^ upon titration with Cu(OAc)_2_ in DMSO. Considering these observations, the UV-vis spectral changes of transmetallation of [Fe(Dp4e4mT)_2_]^+^ with Cu(OAc)_2_ in aqueous solution may be obscured by the broad peak in the Fe complex spectrum (Fig. S18A and B†).

There was no clear evidence of transmetallation upon mixing Cu(OAc)_2_ with [Co(Dp4e4mT)_2_]^+^ or [Pd(Dp4e4mT)Cl] (Fig. S18C–F†), with limited spectral changes being observed. No pronounced spectral alterations were observed for [Co(Dp4e4mT)_2_]^+^ with or without Cu(OAc)_2_ at either pH 7.4 and 5.0 (Fig. S18C and D†). At pH 7.4, [Pd(Dp4e4mT)Cl] underwent hydrolysis of its chlorido ligand to generate [Pd(Dp4e4mT)(OH_2_)]^+^, with a spectrum that is very similar to [Pd(Dp4e4mT)Cl] (Fig. S18E and F†). This reaction occurred regardless of whether Cu(OAc)_2_ was present. Chlorido ligand hydrolysis was somewhat faster at pH 5.0.

The reaction of Cu(OAc)_2_ with [Ni(Dp4e4mT)_2_] was more complicated. In pH 7.4 buffer, the spectral changes were minimal with or without Cu(OAc)_2_ present (Fig. S19A and B†). At pH 5, a more significant spectral change was observed when Cu(OAc)_2_ was added, which was modelled as two consecutive first order reactions (*k*_obs_ 3 × 10^−3^ s^−1^ and 7.1 × 10^−5^ s^−1^; Fig. S19B and Table S2†). The faster step (*t*_½_ ∼4 min) involved only small changes to the initial spectrum, suggesting partial ligand dissociation from [Ni(Dp4e4mT)_2_]. In contrast, the second much slower step (*t*_½_ ∼2½ h) led to a species with a spectrum consistent with [Cu(Dp4e4m)(OH_2_)]^+^ (Fig. S17†). In the absence of Cu(OAc)_2_ at pH 5.0, there was little change to the spectrum of [Ni(Dp4e4mT)_2_] (Fig. S19B,† blue curves). Therefore, transmetallation of [Ni(Dp4e4mT)_2_] by Cu(ii) is possible, but only at pH 5.0, and over quite an extended timescale. This finding is in contrast to the much more reactive Zn(ii) and Ga(iii) complexes below.

At pH 7.4, [Zn(Dp4e4mT)_2_] reacted immediately (during mixing time) with a 10-fold excess of Cu(OAc)_2_ to give a UV-vis spectrum that shifted from a *λ*_max_ of 400 nm ([Zn(Dp4e4mT)_2_]) to a *λ*_max_ of 417 nm ([Cu(Dp4e4mT)(OH_2_)]^+^) (Fig. S19C†). In the absence of Cu(OAc)_2_, no significant reaction of [Zn(Dp4e4mT)_2_] occurred. The results were essentially the same at pH 5.0 (Fig. S19D†) and demonstrates that [Zn(Dp4e4mT)_2_] is labile and highly reactive towards Cu(OAc)_2_ in solution.

Similarly, [Ga(Dp4e4mT)_2_]^+^ reacted immediately and quantitatively with Cu(OAc)_2_ to yield a spectrum consistent with [Cu(Dp4e4mT)(OH_2_)]^+^ (*λ*_max_ 417 nm) at pH 7.4 (Fig. S19E†). Interestingly, the spectrum of [Ga(Dp4e4mT)_2_]^+^ also changed at pH 7.4 without added Cu(OAc)_2_ (Fig. S19E;† blue curves), where a rapid and significant shift of the *λ*_max_ occurred from ∼415 nm to 400 nm (*k*_obs_ 2.2 × 10^−2^ s; *t*_½_ ∼30 s), and was accompanied by a decrease in intensity. A change in the coordination sphere must be taking place due to partial (or complete) dissociation of one tridentate ligand. However, at pH 5, [Ga(Dp4e4mT)_2_]^+^ (*λ*_max_ 415 nm) was stable in the absence of Cu(OAc)_2_ (Fig. S19F;† blue curves). In the presence of Cu(OAc)_2_ at pH 5, complete conversion of [Ga(Dp4e4mT)_2_]^+^ to [Cu(Dp4e4mT)(OH_2_)]^+^ occurred during mixing, with no subsequent spectral changes (Fig. S19F;† blue curves).

In summary, the kinetic results in aqueous solution are consistent with the data in DMSO, although a general trend is that the reactions are much more rapid in water. Both [Zn(Dp4e4mT)_2_] and [Ga(Dp4e4mT)_2_]^+^ are most susceptible to transmetallation with Cu(OAc)_2_

### Electrochemical properties

**Fig. 4:**
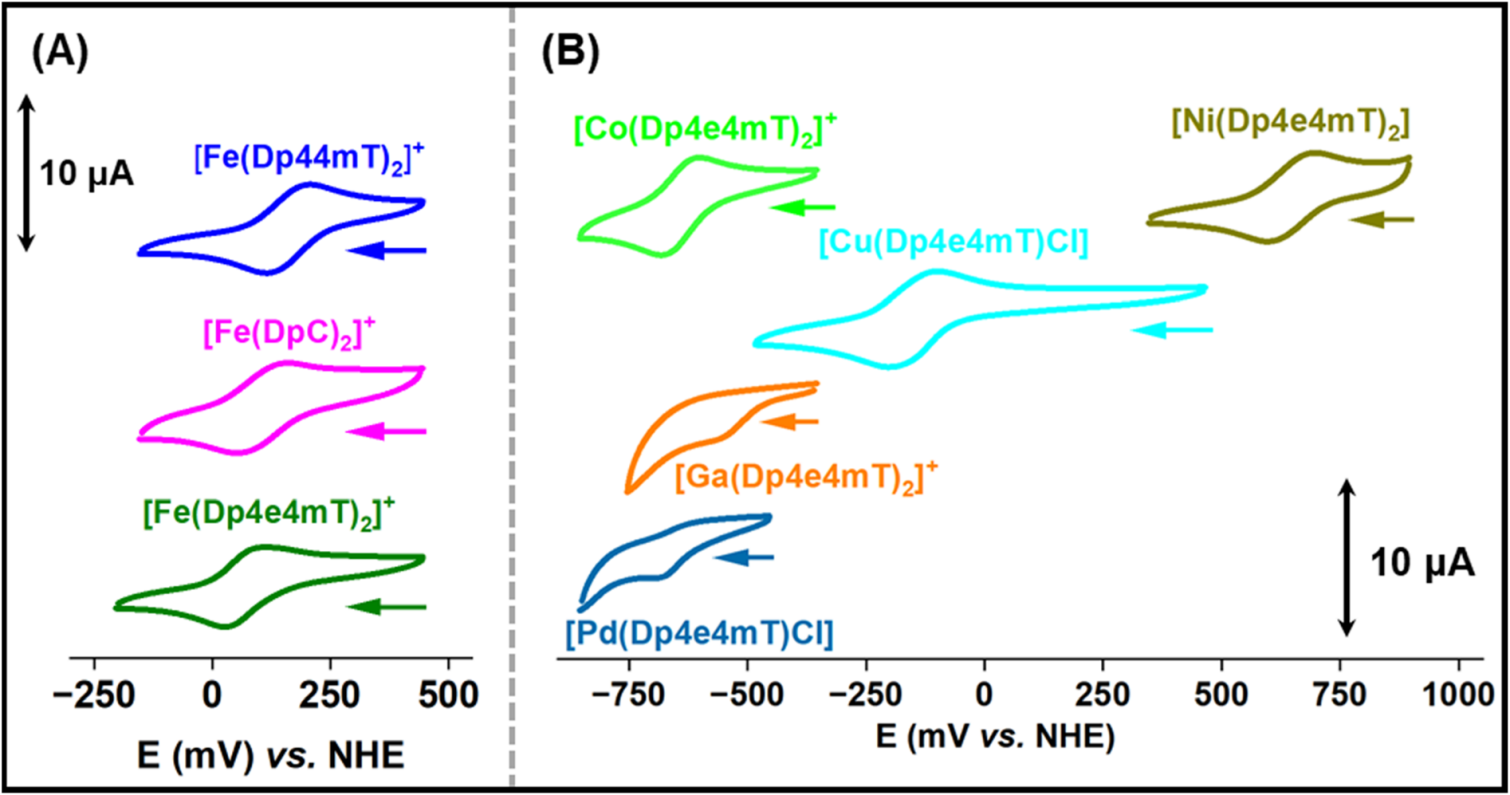
Cyclic voltammograms of: (A) [Fe(Dp44mT)_2_]^+^, [Fe(DpC)_2_]^+^, and [Fe(Dp4e4mT)_2_]^+^. (B) [Co(Dp4e4mT)_2_]^+^, [Ni(Dp4e4mT)_2_], [Cu(Dp4e4mT)Cl], [Ga(Dp4e4mT)_2_]^+^, and [Pd(Dp4e4mT)Cl] (1 mM in MeCN/H_2_O 7 : 3 (v/v) + 0.1 M Et_4_NClO_4_) at a sweep rate of 100 mV s^−1^. The voltammograms were recorded using a glassy carbon working electrode *versus* a non-hydrogen electrode (NHE).

The redox potentials of Fe(iii)- and Cu(ii)-thiosemicarbazone complexes have been extensively studied and are important for understanding intracellular redox activity and ROS generation.^23,27,28,42^ Considering this, the electrochemical properties of [Fe(Dp44mT)_2_]^+^, [Fe(DpC)_2_]^+^, [Fe(Dp4e4mT)_2_]^+^, [Co(Dp4e4mT)_2_]^+^, [Ni(Dp4e4mT)_2_], [Cu(Dp4e4mT)Cl], [Ga(Dp4e4mT)_2_]^+^, and [Pd(Dp4e4mT)Cl] were investigated with cyclic voltammetry in MeCN : H_2_O (70 : 30 v/v; Fig. 4A and B). This solvent system was implemented to provide sufficient solubility and because it has been used by our laboratories previously to measure potentials of thiosemicarbazone complexes enabling effective comparison.^28,45,57–59^ The observed redox potential values of the complexes are listed in Table 1.

**Table 1:** Redox potentials of [Fe(Dp44mT)_2_]^+^, [Fe(DpC)_2_]^+^, [Fe(Dp4e4mT)_2_]^+^, [Co(Dp4e4mT)_2_]^+^, [Ni(Dp4e4mT)_2_], [Cu(Dp4e4mT)Cl], [Ga(Dp4e4mT)_2_]^+^, and [Pd(Dp4e4mT)Cl] in the presence of water [MeCN/H_2_O 7 : 3 (v/v) + 0.1 M Et_4_NClO_4_]

Complexes	Redox potential (mV *vs.* NHE)
[Fe(Dp44mT)_2_]^+/0^	+166
[Fe(DpC)_2_]^+/0^	+149
[Fe(Dp4e4mT)_2_]^+/0^	+70
[Co(Dp4e4mT)_2_]^+/0^	–642
[Ni(Dp4e4mT)_2_]^+/0^	+647
[Cu(Dp4e4mT)(Cl)]^±^	–151
[Ga(Dp4e4mT)_2_]^+/0^	–533 (irreversible)
[Pd(Dp4e4mT)Cl]^±^	–626 (irreversible)

**Fig. 5:**
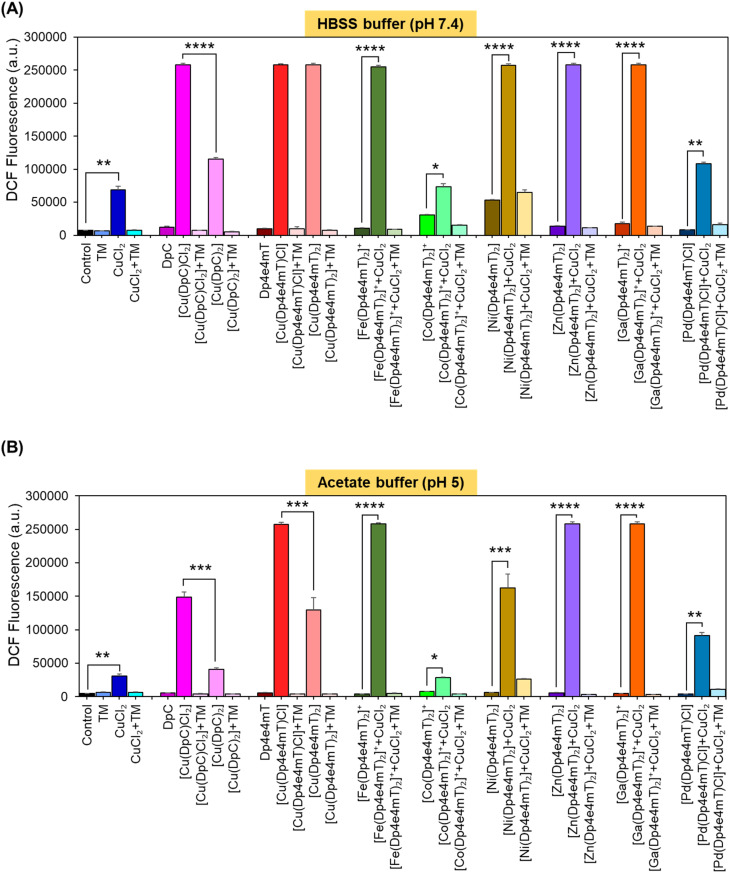
The Ni(ii), Zn(ii), and Ga(iii) complexes of Dp4e4mT transmetallate with Cu(ii) to become markedly redox active. The redox activity of the Cu(ii), Fe(iii), Co(iii), Ni(ii), Zn(ii), Ga(iii), and Pd(ii) complexes of Dp4e4mT (10 μM) were compared to the 1 : 1 and 2 : 1 L : M complexes of DpC by examining the oxidation of The investigations were conducted at: (A) cytoplasmic pH (pH 7.4); and (B) lysosomal pH (pH 5.0). The results are mean ± SD (3 experiments). Statistical significance as indicated on graphs: *p < 0.05; **p < 0.01; ***p < 0.001; ****p < 0.0001.

The Fe(iii) complexes, [Fe(Dp44mT)_2_]^+^, [Fe(DpC)_2_]^+^, and [Fe(Dp4e4mT)_2_]^+^, exhibited reversible Fe(III/II) couples at +166, +149 and +70 mV *versus* NHE, respectively (Table 1 and Fig. 4A). The potentials for [Fe(Dp44mT)_2_]^+^ and [Fe(DpC)_2_]^+^ agree with those reported under the same conditions.^28^ The potential of the [Fe(Dp4e4mT)_2_]^+/0^ couple is appreciably lower than [Fe(Dp44mT)_2_]^+/0^ and [Fe(DpC)_2_]^+/0^ due to the more strong electron donating (*N*-ethyl group) in Dp4e4mT^60^ relative to the dimethyl and cyclohexyl moieties in Dp44 mT and DpC, respectively (Table 1and Fig. 4A). The [Cu(Dp4e4mT)Cl] complex showed reversible a one electron Cu(ii/i) couple in MeCN : H_2_O solution (70 : 30 v/v) at −151 mV *versus* the NHE (Table 1 and Fig. 4B), at a potential accessible to biological reductants, with the redox activity being confirmed below (Fig. 5A and B).

The Ni(ii) complex exhibited a high potential quasi-reversible [Ni(Dp4e4mT)_2_]^+/0^ response at +647 mV *versus* NHE, which is most likely a ligand-centered oxidation rather than a Ni(III/II) process.^45^ The potential of the quasi-reversible [Co(Dp4e4mT)_2_]^+/0^ couple is very low, as expected for a Co(III/II) system, and effectively makes the Co(ii) complex inaccessible to biological reductants. The Ga(iii) and Pd(ii) complexes of Dp4e4mT demonstrated irreversible single electron reductions at low potentials (−533 and −626 mV, respectively), which are most likely ligand-centered redox reactions (Table 1 and Fig. 4B). Due to their low values, these latter complexes are effectively redox-inert (Table 1 and Fig. 4B).

## Biological studies

### The efficacy of Dp4e4mT and its complexes at inhibiting cellular proliferation of SK-N-MC neuroepithelioma cells and AsPC-1 pancreatic cancer cells

The anti-proliferative activity of Dp4e4mT relative to its complexes with Fe(iii), Co(iii), Ni(ii), Cu(ii), Zn(ii), Ga(iii), and Pd(ii) was examined over incubation periods of 24-, 48-, and 72 h/37 °C (Table 2). These studies were performed using human SK-N-MC neuroepithelioma and AsPC-1 pancreatic adenocarcinoma cells that we have previously examined to assay other ligands.^15,28,43^ This activity was compared to three controls, namely the “gold standard” iron chelator for iron overload disease, desferrioxamine (DFO),^61^ that demonstrates poor anti-proliferative activity relative to the potent efficacy of Dp44mT and DpC.^15,33^

**Table 2:** The IC_50_ (μM) values of Dp4e4mT and its Fe(iii), Co(iii), Ni(ii), Cu(ii), Zn(ii), Ga(iii), and Pd(ii) complexes relative to the control agents, Dp44mT, DpC and DFO at inhibiting the growth of SK-N-MC and AsPC-1 cells, and mortal cell-type MRC-5 fibroblasts, as determined by the MTT assay after a 24, 48 and 72 h incubation at 37 °C. Results are mean ± SD (3 experiments)

Compounds	IC_50_ (μM)						
	SK-N-MC			AsPC-1			MRC-5
	24 h	48 h	72 h	24 h	48 h	72 h	72 h
DFO	>100	45.270 ± 0.061	8.184 ± 2.460	>100	22.760 ± 0.084	3.074 ± 0.585	28.910 ± 0.101
Dp44mT	>25	0.043 ± 0.006	0.006 ± 0.001	4.636 ± 0.071	0.042 ± 0.006	0.015 ± 0.003	1.073 ± 0.007
DpC	22.300 ± 0.083	0.088 ± 0.007	0.028 ± 0.009	4.921 ± 0.034	0.087 ± 0.034	0.018 ± 0.007	2.987 ± 0.008
Dp4e4mT	>25	0.043 ± 0.006	0.006 ± 0.001	3.783 ± 0.091	0.045 ± 0.008	0.009 ± 0.002	1.811 ± 0.518
[Fe(Dp4e4mT)_2_]^+^	2.678 ± 0.071	1.531 ± 0.037	0.214 ± 0.005	22.380 ± 0.058	1.581 ± 0.077	0.201 ± 0.031	1.980 ± 0.006
[Co(Dp4e4mT)_2_]^+^	5.498 ± 0.004	1.474 ± 0.061	0.818 ± 0.051	9.522 ± 0.091	2.681 ± 0.078	1.570 ± 0.257	3.901 ± 0.007
[Ni(Dp4e4mT)_2_]	>25	0.075 ± 0.025	0.018 ± 0.006	>25	0.234 ± 0.083	0.076 ± 0.054	>5
[Cu(Dp4e4mT)Cl]	0.492 ± 0.034	0.031 ± 0.008	0.003 ± 0.001	1.074 ± 0.006	0.042 ± 0.006	0.003 ± 0.001	0.391 ± 0.072
[Cu(Dp4e4mT)_2_]	0.516 ± 0.006	0.034 ± 0.006	0.004 ± 0.001	1.142 ± 0.034	0.044 ± 0.007	0.003 ± 0.003	0.342 ± 0.121
[Zn(Dp4e4mT)_2_]	19.350 ± 0.081	0.044 ± 0.007	0.004 ± 0.002	1.728 ± 0.027	0.048 ± 0.007	0.006 ± 0.001	0.756 ± 0.093
[Ga(Dp4e4mT)_2_]^+^	16.220 ± 0.007	0.043 ± 0.006	0.004 ± 0.001	2.431 ± 0.015	0.047 ± 0.006	0.005 ± 0.001	0.774 ± 0.014
[Pd(Dp4e4mT)Cl]	>25	0.661 ± 0.062	0.285 ± 0.121	>25	1.341 ± 0.074	0.262 ± 0.078	1.040 ± 0.011

Assessing the ligands and their anti-proliferative effects in both cell-types demonstrated increased activity as a function of incubation time, with the thiosemicarbazone ligands demonstrating markedly greater efficacy than DFO under all conditions (Table 2). In both cell-types after 72 h, Dp4e4mT was significantly (*p* < 0.01) more active than clinically trialed DpC (IC_50_: 0.018–0.028 μM), and particularly DFO, which demonstrated an IC_50_ value of 3.074–8.184 μM after 72 h (Table 2). The activity of all ligands against SK-N-MC cells was comparable to our previous studies assessing these agents.^15^ Comparing the two cell-types, these latter thiosemicarbazone ligands showed generally similar activity over incubation periods of 48- and 72 h. However, after a 24 h incubation, AsPC-1 cells were more sensitive to Dp4e4mT, Dp44mT, and DpC (IC_50_: 3.783–4.921 μM) relative to SK-N-MC cells (IC_50_: 22.30–>25 μM).

Complexation of Dp4e4mT with metal ions generally caused a pronounced alteration in the anti-proliferative activity of the ligand particularly after a 24 h incubation (Table 2). Both cell-types responded similarly to the complexes in terms of anti-proliferative activity over all incubation times. The exceptions were most prominent after 24 h, where the Ga(iii) and Zn(ii) complexes demonstrated considerably lower efficacy (IC_50_: 16.220–19.350 μM) in SK-N-MC cells than AsPC-1 cells (IC_50_: 1.728–2.431 μM, respectively), while the Fe(iii) complex was significantly (*p* < 0.0001) less effective in AsPC-1 cells (IC_50_ 22.380 μM) than SK-N-MC cells (IC_50_ 2.678 μM; Table 2).

In both cell-types, [Cu(Dp4e4mT)Cl] and [Cu(Dp4e4mT)_2_] demonstrated the most potent anti-proliferative activity of all agents examined (*i.e.*, ligands and complexes) under all incubation conditions (IC_50_: 0.003–0.004 μM after 72 h). The least active complex examined after 72 h in both cell-types was [Co(Dp4e4mT)_2_]^+^ (IC_50_: 0.818–1.570 μM after 72 h; Table 2). Broadly, the complexes can be classed into two groups in terms of anti-proliferative activity against both cell-types (Table 2). That is: Group (A) Dp4e4mT Cu(ii), Zn(ii), and Ga(iii) complexes that demonstrated similar and potent efficacy (IC_50_: 0.003–0.004 μM after 72 h); and Group (B) the significantly (*p* < 0.01–0.05) less potent complexes of (in order of activity): Ni(ii) > Fe(iii) > Pd(ii) > Co(iii) (IC_50_: 0.018–1.570 μM after 72 h). The reason for the strikingly similar activity of the Group (A) agents is probably due to the ability of the Zn(ii) and Ga(iii) complexes to transmetallate with Cu(ii) to generate the potently cytotoxic Cu(ii) complex (Fig. 3F, G, S8B, C, S9B, C, S15A and S16A†). Considering this, our previous studies examining Zn(ii) complexes of DpC and (*E*)-3-phenyl-1-(2-pyridinyl)-2-propen-1-one-4,4-dimethyl-3-thiosemicarbazone (PPP44mT), demonstrate they transmetallate with Cu(ii) in lysosomes, which is an important target for their potent anti-proliferative activity.^26,28^

In contrast, regarding the Group (B) Dp4e4mT Co(iii) and Pd(ii) complexes that possessed the poorest anti-proliferative efficacy, these failed to effectively transmetallate with Cu(ii) (Fig. 3B, C, S3B, C, S4B, C, S12A and S13A†), suggesting its importance in anti-tumor activity. In contrast, the agent with most active anti-proliferative activity in the Group (B) complexes, namely [Ni(Dp4e4mT)_2_], underwent transmetallation with Cu(ii) (Fig. 3D, S5B, C and S14A†), which may, in part, mediate its anti-proliferative activity. Of relevance, the rate of the transmetallation reaction of [Ni(Dp4e4mT)_2_] with Cu(OAc)_2_ was slower than [Ga(Dp4e4mT)_2_]^+^ and especially [Zn(Dp4e4mT)_2_] (Table S2†). This observation may explain the lower anti-proliferative activity of the Ni(ii) complexes of Dp4e4mT *versus* the Zn(ii) and Ga(iii) complexes (Table 2).

Similarly, [Fe(Dp4e4mT)_2_]^+^ also demonstrated little transmetallation with Cu(ii) (Fig. S2A and B†), but its anti-proliferative efficacy was significantly (*p* < 0.001) less than [Ni(Dp4e4mT)_2_] after 48- and 72 h in both cell-types (Table 2). It is possible that [Fe(Dp4e4mT)_2_]^+^ provides the Fe to tumor cells that is a critical nutrient for growth.^62,63^ In fact, other Fe(iii) complexes, such as structurally similar ferric pyridoxal isonicotinoyl hydrazone,^64^ as well as markedly different complexes (*e.g.*, ferric-nitrilotriacetate and ferric citrate)^64,65^ can supply Fe for essential metabolic processes in cells. As such, the ability of [Fe(Dp4e4mT)_2_]^+^ to act as an Fe donor may counter its anti-neoplastic activity.

To examine the selectivity of the Dp4e4mT complexes against neoplastic *versus* normal mortal cells, their anti-proliferative efficacy was compared to mortal MRC-5 fibroblasts after a 72 h/37 °C incubation (Table 3). All agents demonstrated selectivity, with significantly (*p* < 0.0001–0.001) less anti-proliferative activity against MRC-5 cells than either SK-N-MC neuroepithelioma or AsPC-1 pancreatic cancer cells. To further examine selectivity, an *in vitro* therapeutic index was calculated as per our previous investigations.^28,57,59,66,67^ This parameter is derived from the ratio of the IC_50_ of MRC-5 cells/IC_50_ of SK-N-MC or AsPC-1 cells; with high ratios indicating greater selective anti-proliferative activity against neoplastic cells (Table 3). The mean therapeutic index is the average of the therapeutic indices calculated for both tumor cell-types. The lowest mean therapeutic index of 3 was demonstrated for [Co(Dp4e4mT)]^+^ and [Pd(Dp4e4mT)Cl], while the highest (210) was observed for Dp4e4mT (Table 3). The mean therapeutic index of Dp4e4mT exceeded that obtained for clinically trialed DpC (157), as well as that of Dp44mT (112) and DFO (6). Six of the nine Dp4e4mT complexes including those of Cu(ii), Ni(ii), Zn(ii), and Ga(iii) demonstrated high mean therapeutic indices of 99–173, with the Ga(iii) complex demonstrating the greatest value. In contrast, the Dp4e4mT complexes of Co(iii), Fe(iii) and Pd(ii), demonstrated relatively low mean therapeutic indices of 3–9 (Table 3).

**Table 3:** The *in vitro* therapeutic and mean therapeutic index values for DFO, Dp44mT, DpC, Dp4e4mT, and the Dp4e4mT complexes using the neoplastic SK-N-MC and AsPC-1 cell-types relative to mortal MRC-5 fibroblasts after a 72 h/37 °C incubation. Results are from mean IC_50_ values from 3 experiments in each cell-type

Compounds	Therapeutic index		Mean therapeutic index
	SK-N-MC	AsPC-1	
DFO	3	9	6
Dp44mT	152	71	112
DpC	149	165	157
Dp4e4mT	301	120	210
[Fe(Dp4e4mT)_2_]^+^	9	9	9
[Co(Dp4e4mT)_2_]^+^	4	2	3
[Ni(Dp4e4mT)_2_]	250	62	156
[Cu(Dp4e4mT)Cl]	130	130	130
[Cu(Dp4e4mT)_2_]	85	113	99
[Zn(Dp4e4mT)_2_]	187	125	156
[Ga(Dp4e4mT)_2_]^+^	192	154	173
[Pd(Dp4e4mT)Cl]	3	3	3

In summary, the results in Tables 2 and 3 indicate that several complexes, particularly Cu(ii), and those undergoing facile transmetallation, namely Ga(iii) and Zn(ii), demonstrate both marked and selective anti-proliferative activity. Indeed, facile transmetallation of the complex with Cu(ii) was an important property leading to a high therapeutic index.

### Differential redox activity of the Dp4e4mT complexes after addition of Cu(ii): the role of transmetallation

A key criterion for the anti-tumor efficacy of the DpT group of thiosemicarbazones is their ability to form redox active Cu complexes that potently generate ROS, which results in lysosomal membrane permeabilization and apoptosis.^25,26,28^ As such, it was important to assess the redox activity of the Dp4e4mT ligand and its complexes to dissect the mechanism(s) of their anti-proliferative activity observed in Table 2.

Redox activity in cells was examined using an established *in vitro* method examining 2′,7′-dichlorodihydrofluorescein (H_2_DCF) oxidation to fluorescent 2′,7′-dichlorofluorescein (DCF) at cytosolic pH (pH 7.4 using HBSS buffer) relative to lysosomal pH (pH 5; acetate buffer; Fig. 5).^25,26,28,42,46^ To these buffers containing the complexes, l-cysteine (100 μM) was added as a reducing agent, followed by H_2_DCF (5 μM). Then, hydrogen peroxide (H_2_O_2_; 100 μM) was added to initiate hydroxyl radical generation. Both [Cu(DpC)Cl_2_] and [Cu(DpC)_2_] were used as positive controls due to their documented redox activity.^28,42^ In contrast, the ligand, DpC, was implemented as a negative control, as it is redox inert.^28,42^ As an additional control, the established Cu chelator, tetrathiomolybdate (TM), was utilized to remove Cu from the thiosemicarbazone complexes to indicate its role in their redox activity.^27,28,42^ Estimation of H_2_DCF oxidation by measuring fluorescence (*λ*_excitation_ 485 nm and *λ*_emission_ 530 nm) is shown in Fig. 5. Additionally, UV-vis spectroscopy (300–700 nm) of the same solution for each complex was also performed to understand both the oxidation of H_2_DCF and the formation of fluorescent DCF upon excitation at 503 nm (Fig. S20A–F and S21A–F†).

The ligands, TM, DpC, and Dp4e4mT, did not significantly (*p* > 0.05) increase DCF fluorescence relative to the control at cytosolic (pH 7.4) and lysosomal pH (pH 5), suggesting they were redox-inactive (Fig. 5A and B), as described previously for TM and DpC.^28,42^ In contrast, CuCl_2_, [Cu(DpC)_2_], and particularly [Cu(DpC)Cl_2_] significantly (*p* < 0.0001–0.01) increased DCF fluorescence *versus* the control at both pH's (Fig. 5A and B). The 1 : 1 L : M complex [Cu(DpC)Cl_2_] was significantly (*p* < 0.0001–0.001) more active at increasing DCF fluorescence than the 2 : 1 L : M complex, [Cu(DpC)_2_], as demonstrated previously.^45^ The addition of TM to all complexes supplemented with CuCl_2_ significantly (*p* < 0.0001–0.01) diminished DCF fluorescence, indicating the importance of Cu(ii) in the redox activity observed (Fig. 5A and B).

Of all complexes examined in the absence of CuCl_2_, [Cu(Dp4e4mT)Cl], and [Cu(DpC)Cl_2_] demonstrated the greatest efficacy at increasing DCF fluorescence (Fig. 5A and B). Of note, all complexes showed decreased activity at oxidizing H_2_DCF at pH 5 relative to pH 7.4. The redox activity of [Fe(Dp4e4mT)_2_]^+^ was markedly and significantly (*p* < 0.0001) less redox active *versus* Dp4e4mT : Cu(ii) or DpC : Cu(ii) at both pH's (Fig. 5A and B). Similarly, we previously reported the low redox activity of the Dp44mT Fe(iii) complex relative to its Cu(ii) complex.^32^

While the addition of CuCl_2_ significantly (*p* < 0.05) increased the activity of [Co(Dp4e4mT)_2_]^+^ in oxidizing H_2_DCF at both pH's, this was not significantly (*p* > 0.05) different to CuCl_2_ alone (Fig. 5A and B). This lack of activity was validated through UV-vis spectral studies under aqueous conditions where titration of [Co(Dp4e4mT)_2_]^+^ with Cu(OAc)_2_ resulted in no appreciable spectral alteration consistent with transmetallation (Fig. S18C and D†). In contrast, at both pH's, the addition of CuCl_2_ to [Fe(Dp4e4mT)_2_]^+^, [Ni(Dp4e4mT)_2_], [Zn(Dp4e4mT)_2_], and [Ga(Dp4e4mT)_2_]^+^, markedly and significantly (*p* < 0.0001–0.001) increased DCF fluorescence relative to the respective complexes alone (Fig. 5A and B). These results suggested transmetallation to the potently active Cu(ii) complex, and this was validated by LC-MS (Fig. S2A, B, S5B, C, S8B, C, S9B and C†), EPR (Fig. 3A, D, F and G), and UV-vis spectroscopy (Fig. S11A, S14A, S15A and S16A†) in DMSO, and UV-vis spectral studies in aqueous solution (Fig. S18A, B, S19A–F, S20C–F and S21A–D†).

Upon the addition of CuCl_2_ to [Pd(Dp4e4mT)Cl] at both pH's, the redox activity observed was approximately half that demonstrated with the Dp4e4mT Fe(iii), Ni(ii), Zn(ii), and Ga(iii) complexes that undergo transmetallation (Fig. 5A and B). No evidence of transmetallation between Cu(ii) and [Pd(Dp4e4mT)Cl] was observed using LC-MS (Fig. S4B and C†), EPR (Fig. 3C), and UV-vis spectroscopy in DMSO (Fig. S13A†), or under aqueous conditions using UV-vis (Fig. S18E and F†). Similarly to the results observed with [Co(Dp4e4mT)_2_]^+^, the lack of transmetallation of [Pd(Dp4e4mT)Cl] with Cu(ii) could explain its lower anti-proliferative activity (Table 2).

Collectively, these results in Fig. 5A and B indicate that the complexes of Dp4e4mT with Fe(iii), Ni(ii), Zn(ii), and Ga(iii) undergo transmetallation to the potently redox active Cu(ii) complex upon the addition of CuCl_2_, and this could play a role in the marked anti-proliferative activity in tumor cells (Table 2). The inability of the Co(iii) and Pd(ii) complexes to undergo transmetallation results in significantly lower redox activity and anti-proliferative effects (Table 2).

### Differential oxidation of oxy-myoglobin and oxy-hemoglobin by complexes of Dp4e4mT

**Fig. 6:**
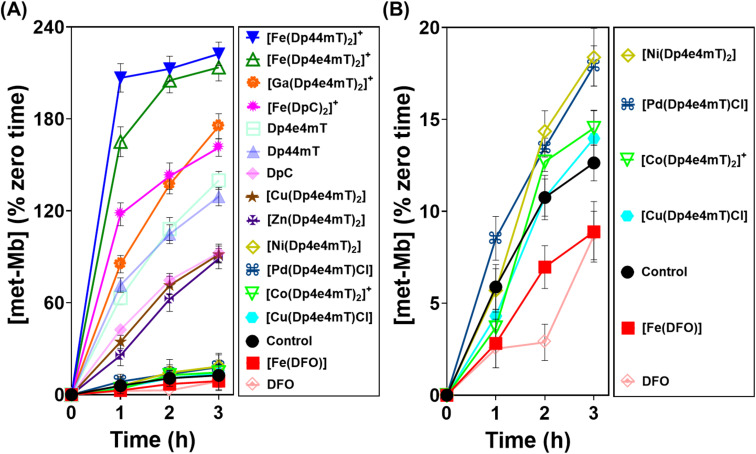
(A and B) Investigation of the effects of Dp4e4mT and its Fe(iii), Co(iii), Ni(ii), Cu(ii), Zn(ii), Ga(iii), and Pd(ii) complexes *versus* the positive controls DFO, Dp44mT, DpC and their Fe(iii) complexes (10 μM) on met-Mb generation (% zero time) after an incubation of 1–3 h/20 °C with purified oxy-Mb (40 μM). Results are mean ± SD (3 experiments).

**Fig. 7:**
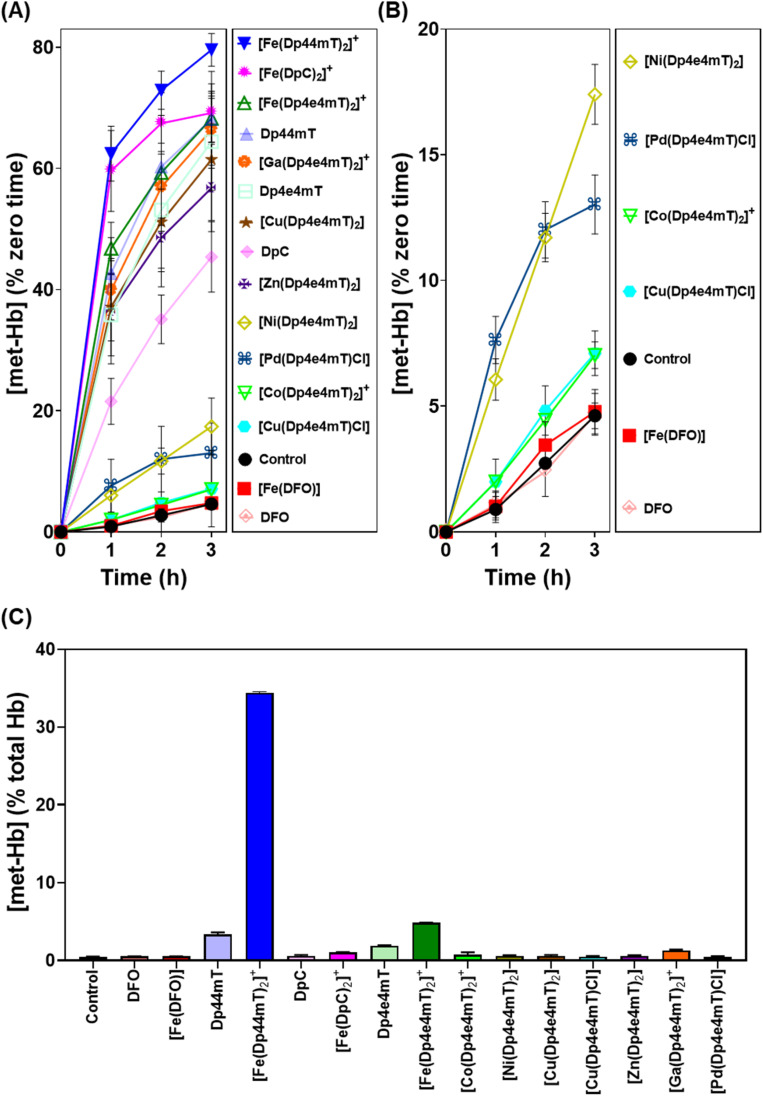
(A and B) Investigation of the effects of Dp4e4mT and its Fe(iii), Co(iii), Ni(ii), Cu(ii), Zn(ii), Ga(iii), and Pd(ii) complexes *versus* DFO, Dp44mT, DpC and their Fe(iii) complexes (10 μM) on met-Hb generation (% zero time) after an incubation of 1–3 h/20 °C with purified oxy-Hb (40 μM). (C) Investigation of the effects of Dp4e4mT and its Fe(iii), Co(iii), Ni(ii), Cu(ii), Zn(ii), Ga(iii), or Pd(ii) complexes relative to the positive controls DFO, Dp44mT, DpC, and their Fe(iii) complexes (10 μM) on oxy-Hb oxidation in intact human erythrocytes after a 3 h/37 °C incubation. Results are mean ± SD (3 experiments).

As demonstrated above in Fig. 5, complexation with metal ions markedly influences the redox behavior of Dp4e4mT. Previous studies demonstrated that the Fe(iii) complexes of clinically trialed thiosemicarbazones such as Triapine®, COTI-2, and DpC resulted in the detrimental oxidation of oxy-Hb and oxy-Mb.^9,39,40,42^ As such, it was important to examine the effect of the Dp4e4mT complexes on oxy-Hb and oxy-Mb oxidation (Fig. 6 and 7). Comparisons were made to three controls, namely DFO, Dp44mT, and DpC, as well as their Fe(iii) complexes. The Fe(iii) complex of DFO acted as a negative control, as it does not induce oxidation of oxy-Mb or oxy-Hb.^28,39^ In contrast, the Fe(iii) complexes of Dp44mT and DpC were positive controls, as they induce oxidation of oxy-Mb.^28,39,42^

Examining the ligands, there was a marked time-dependent increase in oxy-Mb oxidation by Dp4e4mT, Dp44mT, and DpC, to 140%, 129%, and 92% of the control, respectively, after a 3 h incubation (Fig. 6A). However, the corresponding Fe(iii) complexes of these ligands resulted in significantly (*p* < 0.01) higher oxidation of oxy-Mb namely to 213%, 222%, and 161% of the control, respectively. As the ligands themselves are redox inert^28,39^ (Fig. 5), the observed redox activity can be explained by the ability of the ligands to bind contaminating Fe(iii) in solution, generating the redox active Fe(iii) complexes. Recent studies in our laboratories^28,42^ under analogous conditions indicated the presence of chelatable, non-specifically bound iron in the same Mb preparation used herein, supporting this hypothesis.

Regarding the effect of the other complexes on inducing oxy-Mb oxidation, the Group (A) and (B) complexes described above displayed distinct differences (Fig. 6A and B). That is, the Zn(ii) and Ga(iii) complexes of Dp4e4mT in Group (A) induced marked oxy-Mb oxidation, probably because of their facile transmetallation with Fe(iii), as shown by LC-MS (Fig. S8A, B, S9A and B†), EPR (Fig. 3F and G), and UV-vis spectral studies (Fig. S15B and S16B†). In contrast, the Co(iii) and Pd(ii) complexes of Group (B) displayed no appreciable oxidation of oxy-Mb (Fig. 6A and B), as they are redox inert and do not undergo transmetallation with Fe(iii) (Fig. S3A, B, S4A and B†). Considering the low activity of [Ni(Dp4e4mT)_2_] at oxidizing oxyMb (Fig. 6A and B), incomplete transmetallation with FeCl_3_ was observed using LC-MS (Fig. S5A and B†), EPR (Fig. 3D), and UV-vis spectroscopy (Fig. S14B and Table S2†).

The Cu(ii) complexes of Dp4e4mT showed two different effects on oxy-Mb oxidation depending on whether the Cu : L 1 : 1 or 1 : 2 complexes were assessed. The 1 : 1 Cu : L complex ([Cu(Dp4e4mT)Cl]) was demonstrated to be stable to transmetallation upon titration with Fe(iii), as shown by LC-MS (Fig. S7A and B†), EPR (Fig. 3E), and UV-vis spectroscopy (Fig. S11B†), and did not significantly lead to oxidation of oxy-Mb (Fig. 6B). Indeed, of all the thiosemicarbazone complexes examined, [Cu(Dp4e4mT)Cl] demonstrated the lowest activity at causing oxy-Mb oxidation. On the other hand, LC-MS, EPR, and UV-vis spectrophotometry on Cu(ii) complexes of other pyridyl thiosemicarbazones have consistently shown that the [CuL_2_] complexes (including [Cu(Dp4e4mT)_2_]) are unstable in solution and dissociate to [Cu(Dp4e4mT)X] (X = solvent or chloride) and one equivalent of free ligand^27,28,42,45^ (Fig. S6A and B†). It is this free ligand that chelates free Fe(iii) in solution to form the redox active [Fe(Dp4e4mT)_2_]^+^ complex, which in turn, oxidizes oxy-Mb (Fig. 6A).

Our previous studies indicated oxy-Mb was more susceptible than oxy-Hb to the oxidative activity of thiosemicarbazone Fe(iii) complexes to met-Mb and met-Hb, respectively.^28,39^ This observation was again confirmed herein with the Dp4e4mT complexes comparing the oxidation of oxy-Mb (Fig. 6A and B) and oxy-Hb (Fig. 7A and B). Similar trends in oxidative activity of the complexes were observed comparing oxy-Mb and oxy-Hb (*cf.*Fig. 6A, B, 7A and B). However, while the Ni(ii), Pd(ii), and Co(iii) complexes of Dp4e4mT still induced low levels of oxy-Hb oxidation (Fig. 6B), these were more pronounced relative to the control than that observed for oxy-Mb (Fig. 6B). The reason for this observation is unknown, but may be due to a non-specific effect of the complex on oxy-Hb that potentiates heme oxidation.

Physiologically, oxy-Hb is found in erythrocytes, and in the current investigation, the ligands and complexes (10 μM) were incubated with intact human erythrocytes for 3 h/37 °C to assess their effects on oxy-Hb oxidation (Fig. 7C). As expected from our previous studies,^28,39^ DFO, DpC and their respective Fe(iii) complexes did not did not induce any significant (*p* > 0.05) increase in oxy-Hb oxidation to met-Hb in erythrocytes *versus* the control (0.5% of the total; Fig. 7C). In contrast, Dp44mT and particularly [Fe(Dp44mT)_2_]^+^, induced a pronounced and significant (*p* < 0.001) increase in oxy-Hb oxidation to 2.3% and 34.4%, respectively, confirming our previous observations.^28,39^ On the other hand, Dp4e4mT and [Fe(Dp4e4mT)_2_]^+^ were significantly (*p* < 0.0001) less reactive than [Fe(Dp44mT)_2_]^+^ in oxidizing oxy-Hb levels to 1.9% and 4.8%, respectively (Fig. 7C). In marked contrast, the Co(iii), Ni(ii), Cu(ii), Zn(ii), and Pd(ii) complexes of Dp4e4mT had no (*p* > 0.05) significant effect on oxy-Hb oxidation in erythrocytes *versus* the control. Only the Ga(iii) complex of Dp4e4mT slightly, but significantly (*p* < 0.05), increased oxy-Hb oxidation to 1.3% relative to the control (Fig. 7C).

Overall, examining both oxy-Mb and oxy-Hb (Fig. 6 and 7), the ability of the complexes to undergo transmetallation with Fe(iii) was associated with increased oxidation of heme. The thiosemicarbazone complex with lowest ability to oxidize oxy-Mb and oxy-Hb was the 1 : 1 Cu : L complex ([Cu(Dp4e4mT)Cl]), which also demonstrated the greatest anti-proliferative efficacy (Table 2).

## Conclusions

An in-depth investigation has been conducted to comprehend the impact of differential transmetallation of the metal complexes of Dp4e4mT on their redox and biological activities, as well as the detrimental oxidation of oxy-Mb and oxy-Hb. These properties are crucial to understand in terms of their translation as clinically useful anti-cancer agents. Combined LC-MS, EPR, and UV-vis spectroscopy and/or DCF oxidation studies demonstrate the facile transmetallation of [Zn(Dp4e4mT)_2_] and [Ga(Dp4e4mT)_2_]^+^ with Fe(iii) and Cu(ii). Relative to the Zn(ii) and Ga(iii) complexes of Dp4e4mT, its Ni(ii) complex demonstrates kinetically slow transmetallation with Cu(ii) and Fe(iii). The Cu(ii) complexes of Dp4e4mT display a distinctly different mode of activity, with the 1 : 1 [Cu(Dp4e4mT)Cl] complex being stable to transmetallation, while [Cu(Dp4e4mT)_2_] dissociates to [Cu(Dp4e4mT)X] and a free Dp4e4mT ligand that then binds Fe(iii). Our current studies also reveal that [Co(Dp4e4mT)_2_]^+^ and [Pd(Dp4e4mT)Cl] are unreactive and do not undergo transmetallation.

Due to their facile transmetallation with Cu(ii), both [Zn(Dp4e4mT)_2_], and [Ga(Dp4e4mT)_2_]^+^ exhibit potent and strikingly comparable anti-proliferative efficacy to [Cu(Dp4e4mT)Cl] and [Cu(Dp4e4mT)_2_] (*i.e.*, IC_50_ 0.003–0.006 μM after 72 h; Table 2). Moreover, these complexes exhibit high mean therapeutic indices, with the [Ga(Dp4e4mT)_2_]^+^ complex the highest value. In contrast, [Co(Dp4e4mT)_2_] and [Pd(Dp4e4mT)Cl] that are inert to transmetallation demonstrated markedly less potency and very low mean therapeutic indices, correlating with their poor transmetallation activity. Between these extremes, [Ni(Dp4e4mT)_2_] demonstrated intermediate anti-proliferative activity that reflected kinetically slower transmetallation with Cu(ii) than either [Zn(Dp4e4mT)_2_] or [Ga(Dp4e4mT)_2_]^+^.

The complexes, [Co(Dp4e4mT)_2_]^+^, [Ni(Dp4e4mT)_2_], and [Pd(Dp4e4mT)Cl], exhibited minimal impact on both oxy-Mb and oxy-Hb oxidation, probably due to their lack of redox activity and -inability to undergo facile transmetallation with Fe(iii). In contrast, [Zn(Dp4e4mT)_2_] and [Ga(Dp4e4mT)_2_]^+^ significantly increased oxy-Mb and oxy-Hb oxidation, probably *via* transmetallation to [Fe(Dp4e4mT)_2_]^+^. Through a different mechanism, [Cu(Dp4e4mT)_2_] dissociated in solution to the 1 : 1 L : M complex and liberating the free ligand that then binds contaminating Fe(iii) in solution, resulting in the subsequent oxidation of oxy-Mb and oxy-Hb. Under more physiological conditions examining intact erythrocytes, incubation with [Co(Dp4e4mT)_2_]^+^, [Ni(Dp4e4mT)_2_], [Cu(Dp4e4mT)Cl], [Cu(Dp4e4mT)_2_], [Zn(Dp4e4mT)_2_], and [Pd(Dp4e4mT)Cl], had negligible effects on oxy-Hb oxidation, except for a slight effect observed with [Ga(Dp4e4mT)_2_]^+^.

In summary, these studies highlight the critical role of transmetallation on the potent anti-proliferative and selective anti-tumor efficacy of Dp4e4mT complexes and their unfavorable off-target activity *i.e.*, oxy-Mb and oxy-Hb oxidation. Especially notable was the potent anti-proliferative activity of [Cu(Dp4e4mT)Cl] and its marked redox activity, while its lack of transmetallation with Fe(iii) totally prevented detrimental oxidation of oxy-Mb and oxy-Hb. These properties indicates the potential of [Cu(Dp4e4mT)Cl] as an effective anti-proliferative agent. The current investigation examining the transmetallation of Dp4e4mT complexes provides valuable insights into their biological mechanism of action, off-target effects, and their potential therapeutic applications.

## Data availability

Unit cell packing diagrams from crystallography, LC-MS, EPR, UV-vis, and ^1^H-NMR spectra (Fig. S1–S29†) are available in the ESI.† Access to all data can be obtained from the authors upon written request.

## Author contributions

M. D. and D. R. R. conceived the study, designed the investigation, supervised staff and students, wrote and edited the manuscript, and obtained grant funding; D. R. R. designed and characterized Dp4e4mT; M. D. and B. K. synthesized and characterized the complexes, performed experiments and edited manuscript; T. W, V. R., J. R. H., M. A. G, W. L., M. G. A, P. V. B performed experiments, edited the manuscript, wrote some sections, and contributed to interpretation.

## Conflicts of interest

No financial conflict of interest exists.

## Supplementary Material

SC-015-D3SC05723B-s001

SC-015-D3SC05723B-s002
