# Supplementary material for: Differential transmetallation of complexes of the anti-cancer thiosemicarbazone, Dp4e4mT: effects on anti-proliferative efficacy, redox activity, oxy-myoglobin and oxy-hemoglobin oxidation
Source: Chem Sci. 2023 Dec 15;15(3):974–90. doi: 10.1039/d3sc05723b (PMC10793205; doi:10.1039/d3sc05723b)
Supplement: SC-015-D3SC05723B-s001 [file SC-015-D3SC05723B-s001.pdf]

Electronic Supplementary Material (ESI) for Chemical Science.
This journal is © The Royal Society of Chemistry 2023

Differential Transmetallation of Complexes of the Anti-Cancer Thiosemicarbazone, Dp4e4mT: Effects on Anti-Proliferative Efficacy, Redox Activity, and Oxy-Myoglobin and Oxy-Hemoglobin Oxidation

Mahendiran Dharmasivam,[1,2*] Busra Kaya,[2] Tharushi P. Wijesinghe,[2] Vera Richardson,[2] Jeffrey R. Harmer,[3] Miguel A. Gonzalvez,[4] William Lewis,[5] Mahan Gholam Azad,[2] Paul V. Bernhardt,[4] and Des R. Richardson,[1,2,6*]

[1]*Molecular Pharmacology and Pathology Program, Department of Pathology and Bosch Institute, The University of Sydney, Sydney, New South Wales, 2006, Australia;*

[2]*Centre for Cancer Cell Biology and Drug Discovery, Griffith Institute for Drug Discovery, Griffith University, Nathan, Brisbane, Queensland, 4111, Australia;*

[3]*Centre for Advanced Imaging, University of Queensland, Brisbane, Queensland, 4072;*

[4]*School of Chemistry and Molecular Biosciences, University of Queensland, Brisbane, Queensland, 4072;*

[5]*Department of Chemistry, University of Sydney, New South Wales, 2006, Australia; and*

[6]*Department of Pathology and Biological Responses, Nagoya University Graduate School of Medicine, Nagoya 466-8550, Japan.*

*<u>**Corresponding authors**</u>**:** **Dr. Des R. Richardson** and **Dr. Mahendiran Dharmasivam**, Centre for Cancer Cell Biology, Griffith Institute for Drug Discovery, Griffith University, Nathan, Brisbane, 4111, Queensland, Australia. Email: d.richardson@griffith.edu.au; Email: m.dharmasivam@griffith.edu.au

Table of Contents

Supplementary Procedures

General Methods…………..……… ………………………………………………..….….…..S4

Chemicals……..…………..……… ………………………………………….…..….….…..S4

Synthesis of Ligands and Complexes..…………………...………………..…………..…..…S4

Crystallographic Studies………………………..……………………………..………………S8

Electrochemistry….…………………….……………………...………………..……..…….S8

Transmetallation Studies..…………..……………………..…………………………………S8

Cell Culture…...……..……………………………………………………..…………...S9

Cellular Proliferation Assay…………...………………………………………..…………S9

ROS Generation Analysis with H_2DCF……………………………………………………..S10

UV-Vis Spectrophotometry Examining met-Mb and met-Hb Formation…………………...S11

Oxidation of Oxy-Hb in Human Erythrocytes……………………..…………..…………S11

Statistics…………………………….………………………..……….…………….S12

Supplementary Tables

Table S1: Crystal and Refinement Data Table…………..………..……..………..........…..S13

Table S2: Pseudo First Order Rate Constants……..………...………..……….............…S14

Supplementary Figures

Figures S1: Unit cell packing diagrams for Dp4e4mT complexes…....…..…..……………S15

Figures S2-S9: Transmetallation Studies of LC-MS Figures……..……..…..……………S16-S23

Figure S10: EPR Spectra of Fe(III) and Cu(II) complexes..……………..……………......S24

Figures S11-S16: UV-Vis Spectrophotometry Studies of Transmetallation in DMSO…………………………………………………………………………….S25-S30

Figure S17. UV-Vis spectra of complexes in aqueous solutions at pH 7.4…………………S31

Figure S18, S19: UV-Vis Spectrophotometry Studies of Transmetallation in Phosphate and Acetate Buffers………………………………………………………..……...….S32, S33

Figure S20, S21: UV-Vis Spectrophotometry Studies Using DCF……………….......S34, S35

Figures S22-S29: ^{1}H and ^{13}C NMR Spectra of Co(III), Zn(II), Ga(III), and Pd(II) Complexes in d_6-DMSO…………………………………………………………………....S36-S39

Supplementary References

References……………………...………...………………………………………..…......S40

General Methods

The ^1H and ^{13}C NMR spectra were acquired using a Bruker Avance III 500 NMR spectrometer (Billerica, MA) in DMSO-d_6, with the chemical shifts (δ) calibrated relative to the internal standard represented by the solvent peak (**Figures S22-S29**). Electrospray ionization mass spectrometry (ESI-MS) measurements were conducted utilizing a Bruker amaZon SL mass spectrometer. Liquid Chromatography-Mass Spectrometer (LC-MS) measurements were conducted utilizing a Thermo Fisher ISQ™ EM Single Quadrupole Mass Spectrometer (Thermo Fisher, MA). The determination of elements (C, H, N, and S) in the compounds was performed using a Thermo Scientific Flash 2000 CHNS/O analyzer (Waltham, MA). Spectral measurements for electronic absorption were taken using a Shimadzu UV-1800 spectrophotometer (Kyoto, Japan) across the range of 200 to 800 nm.

Chemicals

N-ethylmethylamine, carbon disulfide, NaOH, sodium chloroacetate, hydrochloric acid, hydrazine hydrate, di(2-pyridyl)ketone, $Fe(ClO_4)_3·6H_2O$, $CoCl_2·6H_2O$, $NiCl_2·6H_2O$, $Cu(ClO_4)_2·6H_2O$, $Zn(ClO_4)_2·6H_2O$, $Ga(NO_3)_3·H_2O$, $PdCl_2$, MTT, and DFO were obtained from Sigma-Aldrich (St. Louis, MO). Solvents of analytical grade were purchased from Merck and were used as received without further purification.

Experimental

Synthesis and Characterization of Ligands and Metal Complexes.

The synthesis and characterization of Dp44mT, DpC, Dp4e4mT, $[Fe(Dp44mT)_2]^+$, $[Fe(DpC)_2]^+$, [Cu(Dp44mT)Cl], $[Cu(DpC)Cl_2]$, $[Cu(DpC)_2]$ and [Cu(Dp4e4mT)Cl] were performed as described previously by our laboratory.[1-4]

General Synthetic Procedure for Preparing Metal Complexes

A hot alcoholic solution (15 mL) of Dp4e4mT (1 mmol) was added slowly, with constant stirring, to a hot methanolic solution (15 mL) of either $Fe(ClO_4)_3{\cdot}6H_2O$, $CoCl_2{\cdot}6H_2O$, $NiCl_2{\cdot}6H_2O$, $Cu(ClO_4)_2{\cdot}6H_2O$, $Zn(ClO_4)_2{\cdot}6H_2O$, $Ga(NO_3)_3{\cdot}H_2O$, or $PdCl_2$ (0.5 mmol), and the resulting solution was refluxed for 4 h. After cooling the reaction mixture to room temperature, the resulting solid product was collected by filtration, washed with diethyl ether, dried under vacuum, and recrystallized from methanol.

[Fe(Dp4e4mT)$_2$](ClO$_4$)

Greenish brown crystals (0.5 g). Yield 66%. ESI-MS (positive mode) in MeOH, found mass: 652.17 (100%). Calc. mass for $C_{30}H_{32}N_{10}S_2Fe$, 652.17 $[M - ClO_4]^+$. Anal. Calc. (%) for $C_{30}H_{32}N_{10}S_2Fe{\cdot}ClO_4$: C 47.91, H 4.29, N 18.62, S 8.53. Found: C 47.85, H 4.17, N 18.54, S 8.47. UV-Vis in DMSO [λ_{max} (nm) (ε/M^{-1} cm^{-1})]: 303 (15400), 392 (18100), 481 (5700).

[Co(Dp4e4mT)$_2$](Cl)

Brown crystals (0.5 g). Yield 79%. Single crystals suitable for X-ray diffraction were obtained from the slow evaporation of ethanol. ESI-MS (positive mode) in MeOH, found mass: 655.19 (100%). Calc. mass for $C_{30}H_{32}N_{10}S_2Co$, 655.16 $[M - Cl]^+$. ^{1}H NMR (DMSO-d$_6$) δ 8.88 (d, J = 2.3, 1H), 8.35 (dd, J = 18.9, 7.4 Hz, 1H), 8.16 (td, J = 7.8, 1.7 Hz, 1H), 8.11 (d, J = 4.9 Hz, 1H), 8.03 (td, J = 8.1, 1.2 Hz, 1H), 7.78 (d, J = 8.0 Hz, 1H), 7.67 – 7.64 (m, 1H), 7.57 (t, J = 6.4 Hz, 1H), 3.56 (s, 2H), 3.14 (d, J = 22.8 Hz, 3H), 1.19 (t, J = 7.3 Hz, 3H). ^{13}C NMR (DMSO-d$_6$): δ 180.47, 157.37, 151.35, 150.69, 149.90, 149.14, 148.90, 140.30, 137.20, 128.23, 127.18, 125.52, 45.74, 38.15, 9.00. Anal. Calc. (%) for $C_{30}H_{32}N_{10}S_2Co{\cdot}Cl$: C 52.13, H 4.67, N 20.27, S 9.28. Found: C 52.11, H 4.48, N 20.19, S 9.16. UV-Vis in DMSO [λ_{max} (nm) (ε/M^{-1} cm^{-1})]: 310 (24700), 397 (22900), 455 (11900).

[Ni(Dp4e4mT)$_2$](Cl)

Brown crystals (0.62 g). Yield 90%. Single crystals suitable for X-ray diffraction were obtained from the slow evaporation of methanol. ESI-MS (positive mode) in MeOH, found mass: 655.23 (100%). Calc. mass for $C_{30}H_{33}N_{10}S_2Ni$, 655.17 [M – Cl]$^+$. Anal. Calc. (%) for $C_{30}H_{33}N_{10}S_2Ni{\cdot}Cl$: C 52.08, H 4.81, N 20.24, S 9.27. Found: C 52.03, H 4.79, N 20.18, S 9.20. UV-Vis in DMSO [λ_{max} (nm) (ε/M^{-1} cm^{-1})]: 319 (18300), 380 (18900), 436 (23600).

[Cu(Dp4e4mT)$_2$](ClO$_4$)

Green crystals (0.65 g). Yield: 86%. Single crystals suitable for X-ray diffraction were obtained from the slow evaporation of methanol. ESI-MS (positive mode) in MeOH, found mass: 660.19 (100%). Calc. mass for $C_{30}H_{33}N_{10}S_2Cu$, 660.16 [M – ClO_4]$^+$. Anal. Calc. (%) for $C_{30}H_{33}N_{10}S_2Cu{\cdot}ClO_4$: C 47.36, H 4.37, N 18.41, S 8.43. Found: C 47.12, H 4.21, N 18.24, S 8.24. UV-Vis in DMSO [λmax (nm) (ε/M−1 cm−1)]: 311 (18900), 355 (14900), 433 (15600).

[Zn(Dp4e4mT)$_2$]

Yellow crystals (0.55 g). Yield: 89%. Single crystals suitable for X-ray diffraction were obtained from the slow evaporation of methanol. ESI-MS (positive mode) in MeOH, found mass: 661.24 (100%). Calc. mass for $C_{30}H_{32}N_{10}S_2Zn$, 661.15 [M + H]$^+$. ^{1}H NMR (DMSO-d$_6$) δ 8.77 (d, *J* = 4.9 Hz, 1H), 8.03 – 7.97 (m, 2H), 7.91 (d, *J* = 7.3 Hz, 1H), 7.75 (td, *J* = 8.0, 1.5 Hz, 1H), 7.50-7.46 (m, 1H), 7.31 (dd, *J* = 6.8, 5.5 Hz, 1H), 7.18 (d, *J* = 8.0 Hz, 1H), 3.60 (s, 2H), 3.09 (s, 3H), 1.19 – 1.00 (m, 3H). ^{13}C NMR (DMSO-d$_6$): δ 181.59, 153.30, 149.98, 149.52, 146.05, 143.65, 138.95, 136.37, 127.41, 124.21, 123.87, 123.42, 46.25, 37.81, 12.65. Anal. Calc. (%) for $C_{30}H_{32}N_{10}S_2Zn$: C 54.42, H 4.87, N 21.15, S 9.69. Found: C 54.38, H 4.71, N 21.07, S 9.57. UV-Vis in DMSO [λ_{max} (nm) (ε/M^{-1} cm^{-1})]: 316 (23600), 423 (46500).

[Ga(Dp4e4mT)$_2$](NO$_3$)

Orange crystals (0.64 g). Yield 88%. Single crystals suitable for X-ray diffraction were obtained from the slow evaporation of methanol. ESI-MS (positive mode) in MeOH, found mass: 665.21 (100%). Calc. mass for $C_{30}H_{32}N_{10}S_2Ga$, 665.15 $[M - NO_3]^+$. ^{1}H NMR (DMSO-d$_6$) δ 8.88 (d, *J* = 4.9, 1H), 8.32 (dd, *J* = 25.5, 7.8 Hz, 1H), 8.18-8.16 (m, 3H), 7.82 – 7.77 (m, 1H), 7.69 –7.66 (m, 2H), 3.64 (d, *J* = 6.7 Hz, 2H), 3.19 (s, 3H), 1.06 (t, *J* = 6.7 Hz, 3H). ^{13}C NMR (DMSO-d$_6$): δ 176.42, 153.30, 149.87, 149.70, 145.42, 144.76, 142.64, 132.71, 137.04, 127.53, 126.36, 125.61, 48.29, 36.18, 12.69. Anal. Calc. (%) for $C_{30}H_{32}N_{10}S_2Ga{\cdot}NO_3$: C 49.46, H 4.43, N 21.15, S 8.80. Found: C 49.38, H 4.28, N 21.08, S 8.64. UV-Vis in DMSO [λ_{max} (nm) (ε/M^{-1} cm^{-1})]: 306 (15800), 428 (31000).

[Pd(Dp4e4mT)Cl]

Orange crystals (0.37 g). Yield 83%. Single crystals suitable for X-ray diffraction were obtained from slow evaporation of a methanol solution. ESI-MS (positive mode) in MeOH, found mass: 404.60 (100%). Calc. mass for $C_{15}H_{16}N_5SPd$, 404.81 $[M - Cl]^+$. ^{1}H NMR (DMSO-d$_6$) δ 8.76 (dd, *J* = 4.8, 1.7, 1H), 8.62 (d, *J* = 4.6 Hz, 1H), 8.03 (td, *J* = 7.9, 1.5 Hz, 1H), 8.00 – 7.95 (m, 1H), 7.90 – 7.82 (m, 1H), 7.61 (t, *J* = 6.4 Hz, 1H), 7.51-7.55 (m, 1H), 7.39 (d, *J* = 7.8 Hz, 1H), 3.55 (s, 2H), 3.15 (d, *J* = 21.5 Hz, 3H), 1.16 – 0.97 (m, 3H). ^{13}C NMR (DMSO-d$_6$): δ 180.78, 158.58, 150.68, 150.19, 149.78, 148.62, 140.99, 136.67, 127.91, 126.74, 125.83, 125.12, 47.68, 38.03, 13.07. Anal. Calc. (%) for $C_{15}H_{16}ClN_5SPd$: C 40.92, H 3.66, N 15.91, S 7.28. Found: C 40.89, H 3.62, N 15.87, S 7.21. UV-Vis in DMSO [λ_{max} (nm) (ε/M^{-1} cm^{-1})]: 305 (14600), 398 (16000), 486 (6400).

X-ray Crystallography

Single crystals of Co(III), Ni(II), Cu(II), Zn(II), Ga(III), and Pd(II) Dp4e4mT complexes were **obtained from a slow concentration of methanolic and ethanolic solutions**. A suitable crystal was selected and **mounted on a loop using Paratone® protective oil. M**irror monochromated Cu Kα radiation (1.54184 Å) was used on either a SuperNova Rigaku Oxford Diffraction System with an **Atlas** CCD area detector or an Oxford Diffraction Gemini Ultra S diffractometer. Using Olex2,[5] the structure was solved with SHELXS[6] structure solution program using direct methods and refined with the SHELXL[7] refinement package using least squares minimization.

Electrochemistry

Cyclic voltammograms were acquired for the Fe(III), Co(III), Ni(II), Cu(II), Ga(III), and Pd(II) complexes of Dp4e4mT utilizing a BAS100B/W potentiostat, equipped with an aqueous Ag/AgCl reference electrode, a glassy carbon working electrode, and a Pt wire auxiliary electrode. The complexes were dissolved at 1 mM in MeCN:H_2O (70:30 v/v) with Et_4NClO_4 (0.1 M) as the supporting electrolyte.[8] Prior to measurements, the complex solutions were purged with N_2.

Transmetallation Studies

The Dp4e4mT metal complexes were mixed in equimolar amounts with either $FeCl_3$ or $Cu(OAc)_2$ and incubated for 24 h at room temperature in DMSO. Then, the solution was further diluted in a 1:1 ratio with MeOH, and then analyzed using LC-MS with a Thermo Fisher ISQ™ EM Single Quadrupole Mass Spectrometer (Thermo Fisher, MA).

Transmetallation was also monitored using continuous-wave (CW) X-band (*ca.* 9.42 GHz, 105 K) electron paramagnetic resonance (EPR) spectroscopy, which was recorded on a Bruker Elexsys E540 spectrometer equipped with an ElexSys Super High Sensitivity Probehead and LN2 cooling (Bruker ER4141VT temperature control unit). Spectra were simulated with EasySpin.[9]

Time-dependent UV-Vis absorption spectra were measured on an Agilent 8453 diode array UV−Vis spectrophotometer, equipped with a thermostatted (± 0.1 °C) multicell transport cooled by a Huber Ministat 230 recirculating cooling bath. Spectral analysis was carried out with Reactlab Kinetics.[10]

Biological Studies

Cell Culture

All cell lines were procured from the American Type Culture Collection (ATCC; Manassas, VA). The SK-N-MC neuroepithelioma cell line and the mortal MRC-5 fibroblasts were cultured in minimum essential medium (Sigma-Aldrich), while the AsPC-1 cell line was cultured in Roswell Park Memorial Institute 1640 medium (Thermo Fisher Scientific, MA, USA). Both media were supplemented with 10% fetal calf serum, non-essential amino acids (1 mM), sodium pyruvate (1 mM), L-glutamine (2 mM), penicillin (100 U/mL), streptomycin (100 U/mL), and Fungizone (0.5 µg/mL). Cells were maintained at 37 °C in an incubator under a humidified atmosphere with 5% CO_2, following established protocols.[11]

Cellular Proliferation Assay

Cellular proliferation was evaluated through the established 3-(4,5-dimethylthiazol-2-yl)-2,5-diphenyltetrazolium bromide (MTT) proliferation assay, validated by viable cell counts.[12]

Ligands and complexes were dissolved in DMSO to make a stock solution of 10 mM, subsequently diluted in culture media. For cell culture experiments, the DMSO's maximum concentration remained below 0.5% (v/v), with no discernible impact on proliferation.[12] MTT color development was found to be directly proportional to viable cell count measured *via* Trypan blue staining.[12] Analysis of the data was executed employing MARS Data Analysis Software (BMG LabTech; version 3.30). The concentration of agents necessary to inhibit cellular proliferation by 50% (IC_{50}) was then calculated.

ROS Generation Analysis with H_2DCF-DA

The generation of reactive oxygen species (ROS) was assessed by examining the oxidation of H_2DCF.[2, 8, 13, 14] Solutions containing $CuCl_2$, TM, DpC, Dp4e4mT, $[Fe(DpC)_2]^+$, $[Cu(DpC)Cl_2]$, $[Cu(DpC)_2]$ and the Dp4e4mT complexes were prepared at a concentration of 10 μM in either HBSS (pH 7.4) or 150 mM acetate buffer (pH 5) to simulate the conditions of the cytosol and lysosome, respectively.[2, 8, 14, 15] The H_2DCF was prepared by deacetylation of DCFH-DA (Sigma-Aldrich) through alkaline hydrolysis using NaOH.[16]

In these prepared solutions, L-cysteine (100 μM) was added as a reducing agent and then H_2DCF (5 μM) added. The initiation of hydroxyl radical generation was achieved by the addition of hydrogen peroxide (H_2O_2; 100 μM). As a comparative benchmark, HBSS or acetate buffer containing L-cysteine, H_2DCF, and H_2O_2, but lacking ligands or metal complexes, was employed as a control. Fluorescence measurements were carried out using a CLARIOstar Plus microplate reader (BMG LABTECH, Australia) with an excitation wavelength of 485 nm and an emission wavelength of 530 nm.[8, 14]

Spectroscopic Examination of Met-Mb and Met-Hb Formation from Oxy-Mb and Oxy-Hb in Solution

Oxy-Mb and oxy-Hb solutions were prepared following established protocols.[8, 17] In brief, solutions of reduced Mb and Hb in PBS (pH 7.2) were prepared by adding 1.2 mL of $Na_2S_2O_4$ (12 mM) to 38 mL of met-Mb and met-Hb solution (40 μM). [17] Subsequently, oxy-Mb and oxy-Hb were generated by gently passing 150 mL of oxygen (from an air-filled syringe) through the solution containing reduced Mb and Hb, effectively eliminating any residual $Na_2S_2O_4$. Following this, the oxy-Mb and oxy-Hb solutions were passed through a PD-10 column prepacked with 8.3 mL of Sephadex G-25 and equilibrated with PBS (pH 7.2). [17]

In experiments examining the activity of the agents, all complexes were dissolved in 1,2-propanediol, then diluted to 10 μM in PBS (pH 7.2). The experiments were conducted over incubations of 0 to 3 h with the agents. UV-Vis spectra covering the range of 300 to 700 nm were recorded at a temperature of 20°C using a Shimadzu UV−Vis spectrophotometer (UV-1800; Shimadzu, Kyoto, Japan). Concentrations of met-Mb and met-Hb, as well as reduced-Mb and reduced-Hb, were determined at 409 nm and 435 nm, respectively. Concentrations of oxy-Mb and oxy-Hb were determined at 544 nm and 582 nm, respectively.[17]

Oxidation of Oxy-Hb in Human Erythrocytes

Oxidation of oxy-Hb in human erythrocytes was performed by standard methods using fresh blood from healthy donors after collection in Vacutainer® collection tubes (BD, Plymouth, UK).[8] Erythrocytes centrifuged (480 xg/5 min/4°C) and then washed in HBSS, and subsequently resuspended in this buffer. Incubation of the erythrocytes (15% hematocrit) in the absence or presence of the agents (10 μM) was performed for 3 h/37°C. The erythrocytes were

then lysed with ultra-pure water, and the debris removed by centrifugation (16,000g/30 min/ 4°C). The supernatant was used for the assessment of oxy-Hb oxidation by UV-Vis spectrophotometry, as described above.

Statistics

Data were analysed using Student's *t*-test. The results are presented as the mean ± standard deviation (SD) and were considered statistically significant when $p < 0.05$.

Table S1 Crystal data and structure refinement for the Dp4e4mT complexes.

**Parameters**	**[Co(Dp4e4mT)₂]Cl·1½H₂O ·½EtOH**	**[Ni(HDp4e4mT)₂] Cl₂·H₂O**	**[Cu(Dp4e4mT) (HDp4e4mT)] (ClO₄)·H₂O**	**[Zn(Dp4e4mT)₂]**	**[Ga(Dp4e4mT) (HDp4e4mT)](NO₃)₂· 2¾H₂O**	**[Pd(Dp4e4mT)Cl]**
CCDC	2299821	1959772	1982608	1959773	1959775	1959776
Formula	$C_{31}H_{38}ClCoN_{10}O_2S_2$	$C_{30}H_{36}Cl_2N_{10}NiOS_2$	$C_{30}H_{35}ClCuN_{10}O_5S_2$	$C_{30}H_{32}N_{10}S_2Zn$	$C_{30}H_{38.5}GaN_{12}O_{8.75}S_2$	$C_{15}H_{16}ClN_5PdS$
M.W	741.21	746.42	778.79	662.14	746.52	440.24
Crystal system	Monoclinic	triclinic	triclinic	triclinic	monoclinic	triclinic
Space group	*C2/c*	$P\bar{1}$	$P\bar{1}$	$P\bar{1}$	*Cc*	$P\bar{1}$
a (Å)	22.775(1)	9.141(3)	9.5625(3)	10.732(1)	22.3028(3)	10.0507(3)
b (Å)	14.2349(7)	14.090(4)	13.6735(4)	10.827(1)	22.7143(3)	10.5184(3)
c (Å)	23.3316(9)	14.188(3)	13.8397(5)	14.744(1)	14.6945(3)	17.5993(4)
α (°)		94.82(2)	73.376(3)	109.844(9)		73.219(2)
β (°)	91.509(4)	92.36(2)	77.366(3)	91.411(7)	90.769(2)	81.923(2)
γ (°)		107.67(3)	86.693(3)	103.463(9)		69.997(3)
V ($Å^3$)	7561.3(6)	1730.8(9)	1691.9(1)	1556.9(3)	7443.5(2)	1672.07(8)
T (K)	190(2)	100(2)	100(2)	100(2)	100(2)	100(2)
Z	8	2	2	2	8	4
R1 (obsd data)	0.0752	0.1045	0.0407	0.0362	0.0432	0.0307
wR_2	0.2142	0.2267	0.1090	0.0938	0.1204	0.0803
GOF	1.032	0.967	1.022	1.054	1.042	1.059

Table S2. Observed pseudo first order rate constants (k_{obs}, s^{-1}) for reactions of each Dp4e4mT complex with a 10-fold excess of $Cu(OAc)_2$ or $FeCl_3$ in DMSO or water (pH 7.4 or 5.0) at 25 °C.

complex	DMSO		$Cu(OAc)_2$ in water	
	$FeCl_3$	$Cu(OAc)_2$	pH 7.4	pH 5
$[Fe(Dp4e4mT)_2]^+$	-	$1.7(1) \times 10^{-3}$<br>$1.7(1) \times 10^{-4}$	[a]	[a]
$[Co(Dp4e4mT)_2]^+$	No reaction	No reaction	No reaction	No reaction
$[Pd(Dp4e4mT)Cl]$[b]	No reaction	No reaction	$2.1(1) \times 10^{-4}$	$7.8(1) \times 10^{-4}$
$[Ni(Dp4e4mT)_2]$	No reaction	$9.9(1) \times 10^{-5}$	No reaction	$9.0(1) \times 10^{-4}$<br>$8.7(1) \times 10^{-5}$
$[Cu(Dp4e4mT)Cl]$	No reaction	-	-	-
$[Zn(Dp4e4mT)_2]$	$8.9(1) \times 10^{-3}$	> 0.1	> 0.1	> 0.1
$[Ga(Dp4e4mT)_2]^+$	$4.7(1) \times 10^{-5}$	$6.2(1) \times 10^{-5}$	> 0.1	> 0.1

[a] Reaction could not be deciphered by UV-Vis spectroscopy; [b]chlorido ligand hydrolysis

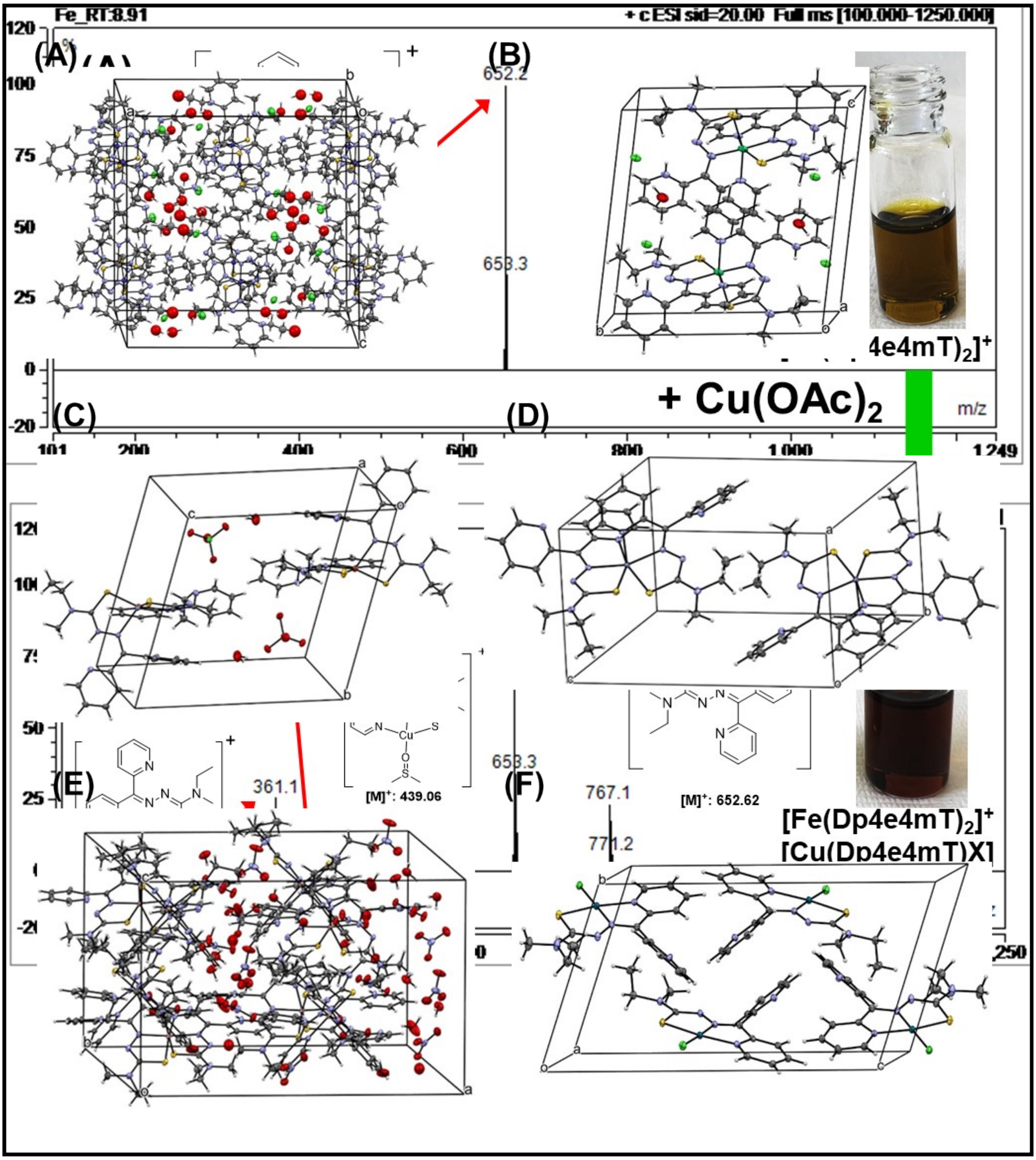

Figure S1. Unit cell packing diagrams for: **(A)** $[Co(Dp4e4mT)_2]Cl·1½H_2O·½EtOH$; **(B)** $[Ni(HDp4e4mT)_2]Cl_2·H_2O$; **(C)** $[Cu(Dp4e4mT)(HDp4e4mT)](ClO_4)·H_2O$; **(D)** $[Zn(Dp4e4mT)_2]$, **(E)** $[Ga(Dp4e4mT)(HDp4e4mT)](NO_3)_2·2¾H_2O$; and **(F)** [Pd(Dp4e4mT)Cl].

Figure S2. (A, B) Transmetallation of $[Fe(Dp4e4mT)_2]^+$ was examined by LC-MS upon the addition of 1 equivalent of $Cu(OAc)_2$. A 1:1 ratio of $[Fe(Dp4e4mT)_2]^+$ to $Cu(OAc)_2$ was prepared in DMSO and incubated for 24 h/20 °C. LC-MS was then performed.

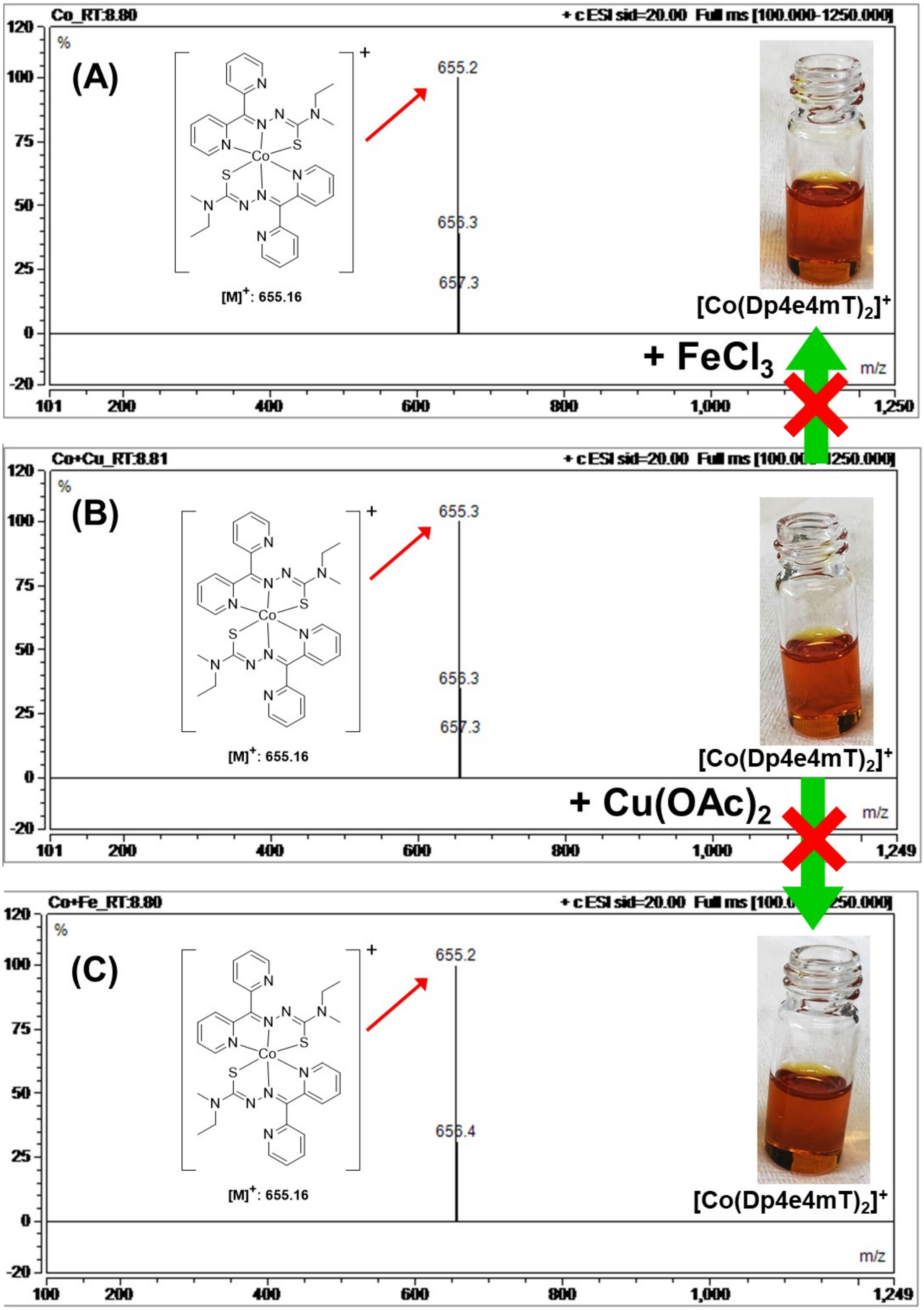

Figure S3. (A-C) Transmetallation of $[Co(Dp4e4mT)_2]^+$ was examined by LC-MS upon the addition of 1 equivalent of $FeCl_3$ or $Cu(OAc)_2$. A 1:1 ratio of $[Co(Dp4e4mT)_2]^+$ to metal salts ($FeCl_3$ or $Cu(OAc)_2$) was prepared in DMSO and incubated for 24 h/20 °C. LC-MS was then performed.

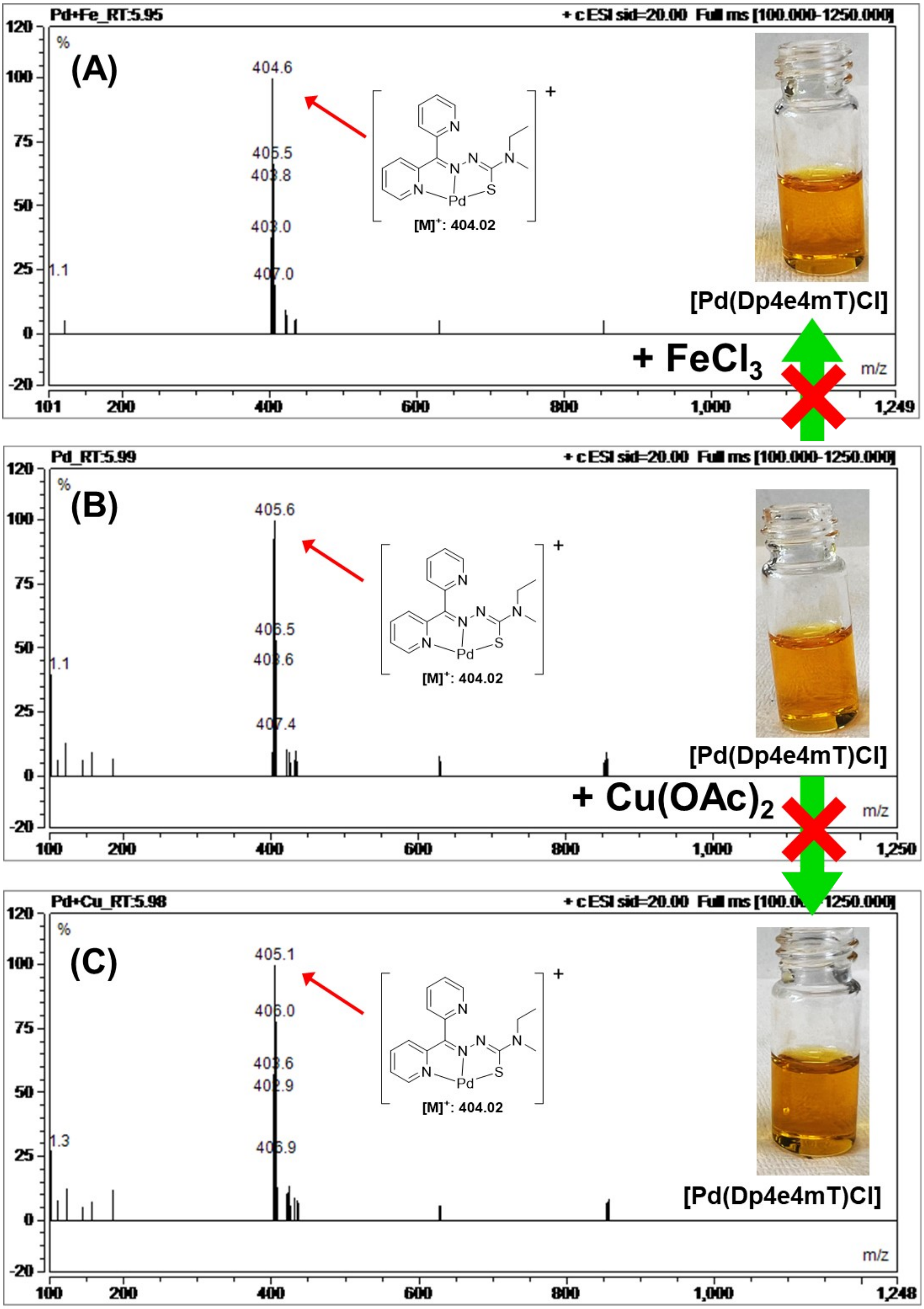

Figure S4. (A-C) Transmetallation of [Pd(Dp4e4mT)Cl] was examined by LC-MS upon the addition of 1 equivalent of $FeCl_3$ or $Cu(OAc)_2$. A 1:1 ratio of [Pd(Dp4e4mT)Cl] to metal salts ($FeCl_3$ or $Cu(OAc)_2$) was prepared in DMSO and incubated for 24 h/20 °C. LC-MS was then performed.

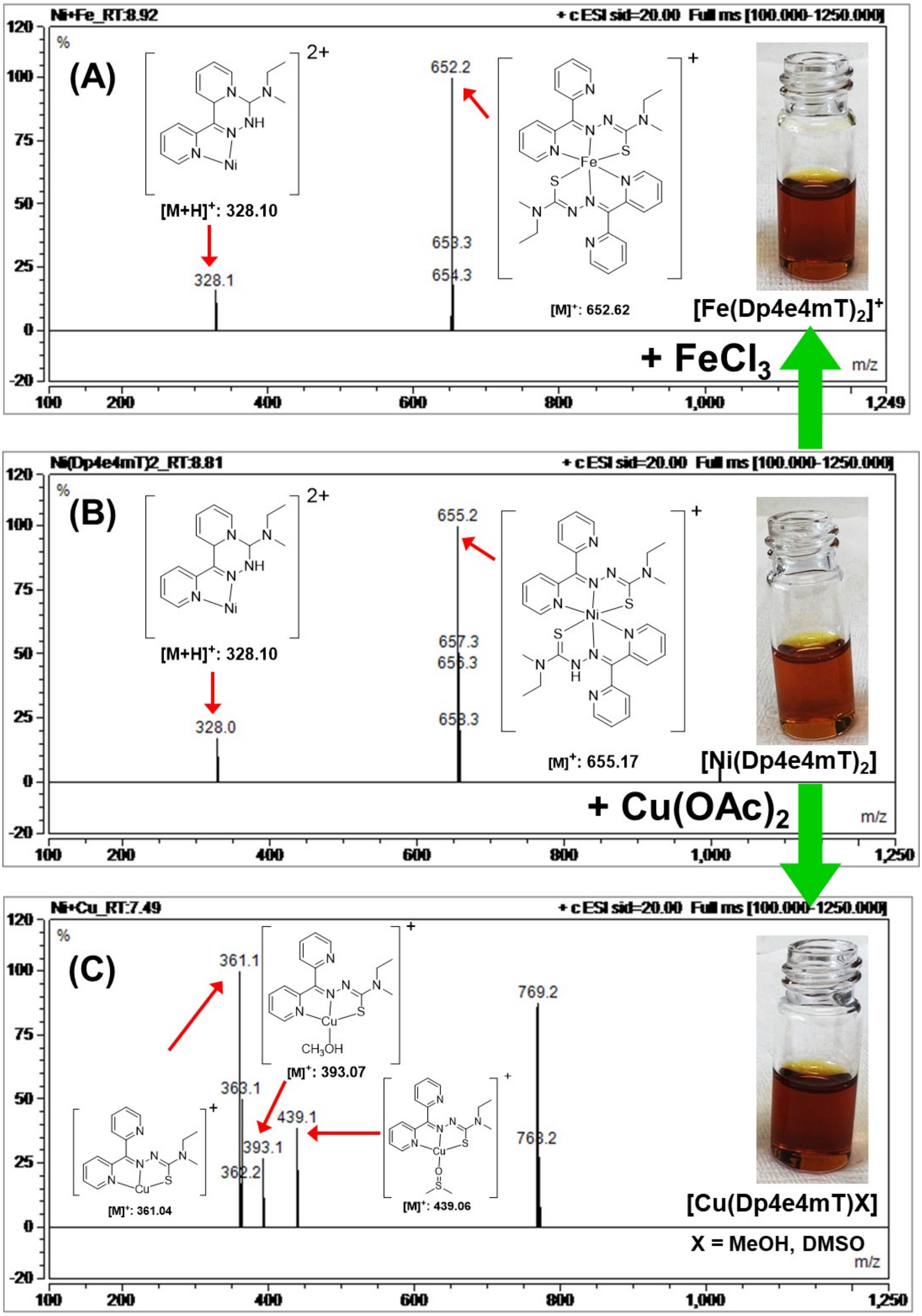

Figure S5. (A-C) Transmetallation of [Ni(Dp4e4mT)$_2$] was examined by LC-MS upon the addition of 1 equivalent of $FeCl_3$ or $Cu(OAc)_2$. A 1:1 ratio of [Ni(Dp4e4mT)$_2$] to metal salts ($FeCl_3$ or $Cu(OAc)_2$) was prepared in DMSO and incubated for 24 h/20 °C. LC-MS was then performed.

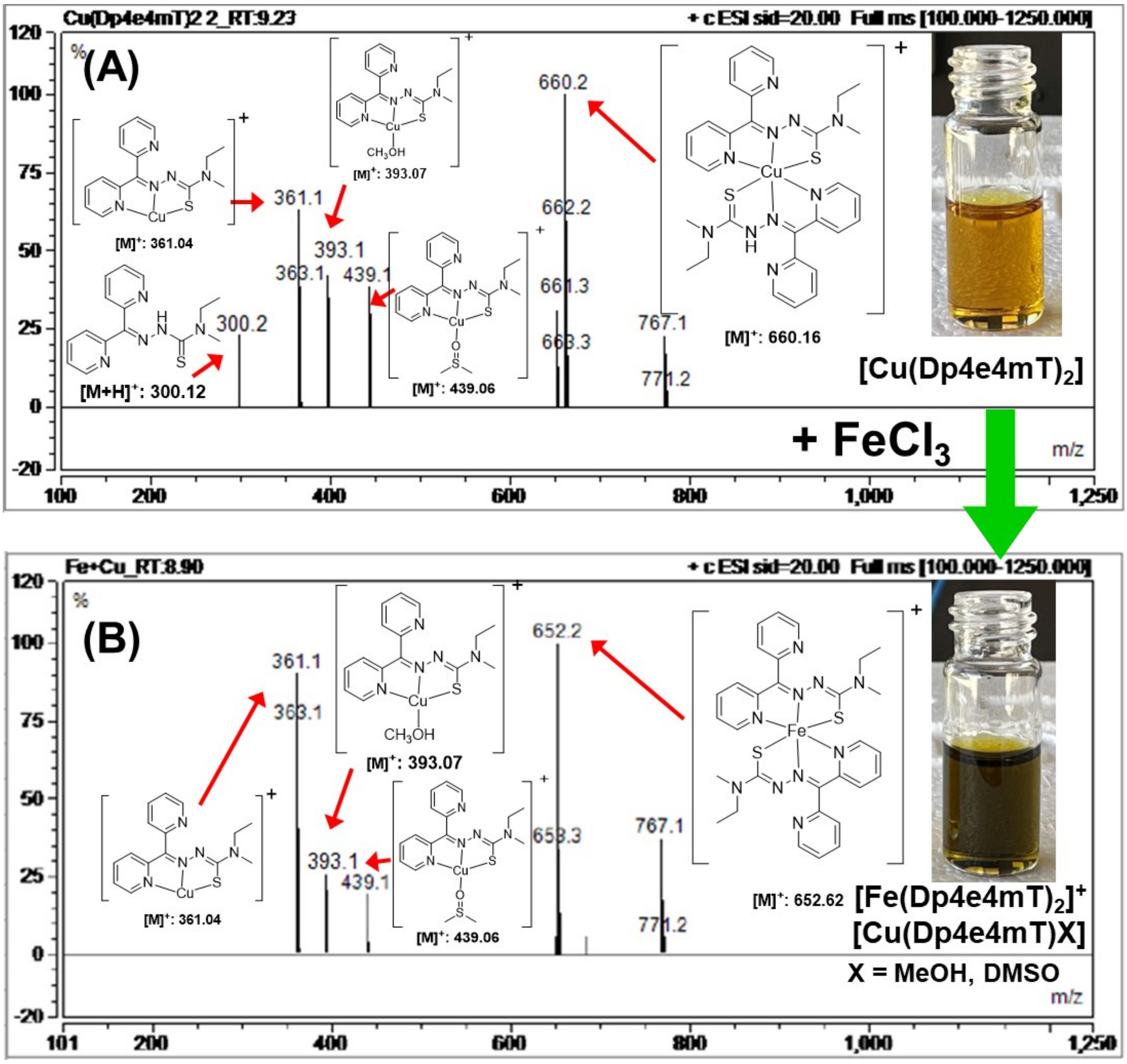

Figure S6. (A, B) Transmetallation of [Cu(Dp4e4mT)$_2$] was examined by LC-MS upon the addition of 1 equivalent of $FeCl_3$. A 1:1 ratio of [Cu(Dp4e4mT)$_2$] to $FeCl_3$ was prepared in DMSO and incubated for 24 h/20 °C. LC-MS was then performed.

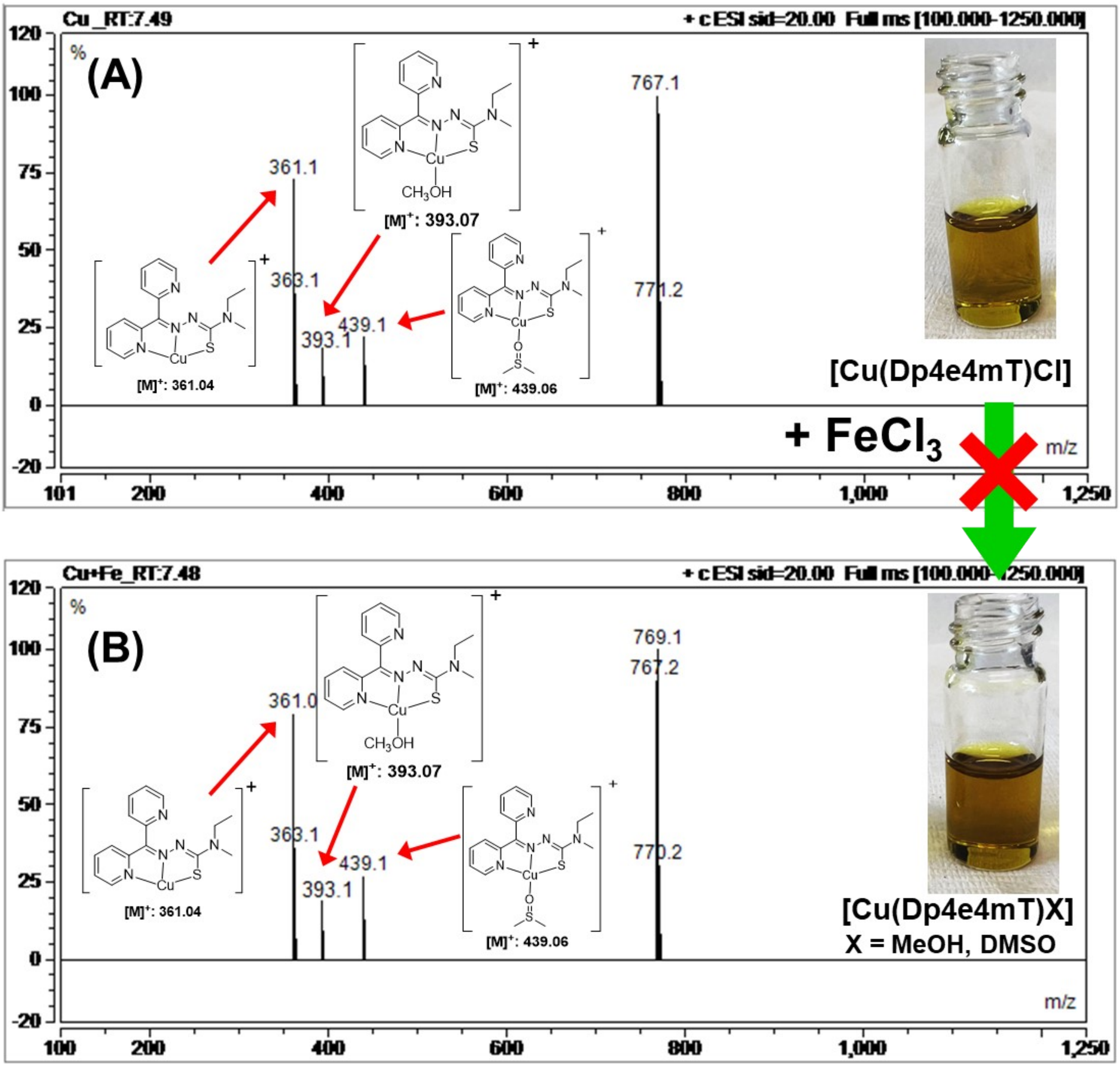

Figure S7. (A, B) Transmetallation of [Cu(Dp4e4mT)Cl] was examined by LC-MS upon the addition of 1 equivalent of $FeCl_3$. A 1:1 ratio of [Cu(Dp4e4mT)Cl] to $FeCl_3$ was prepared in DMSO and incubated for 24 h/20 °C. LC-MS was then performed.

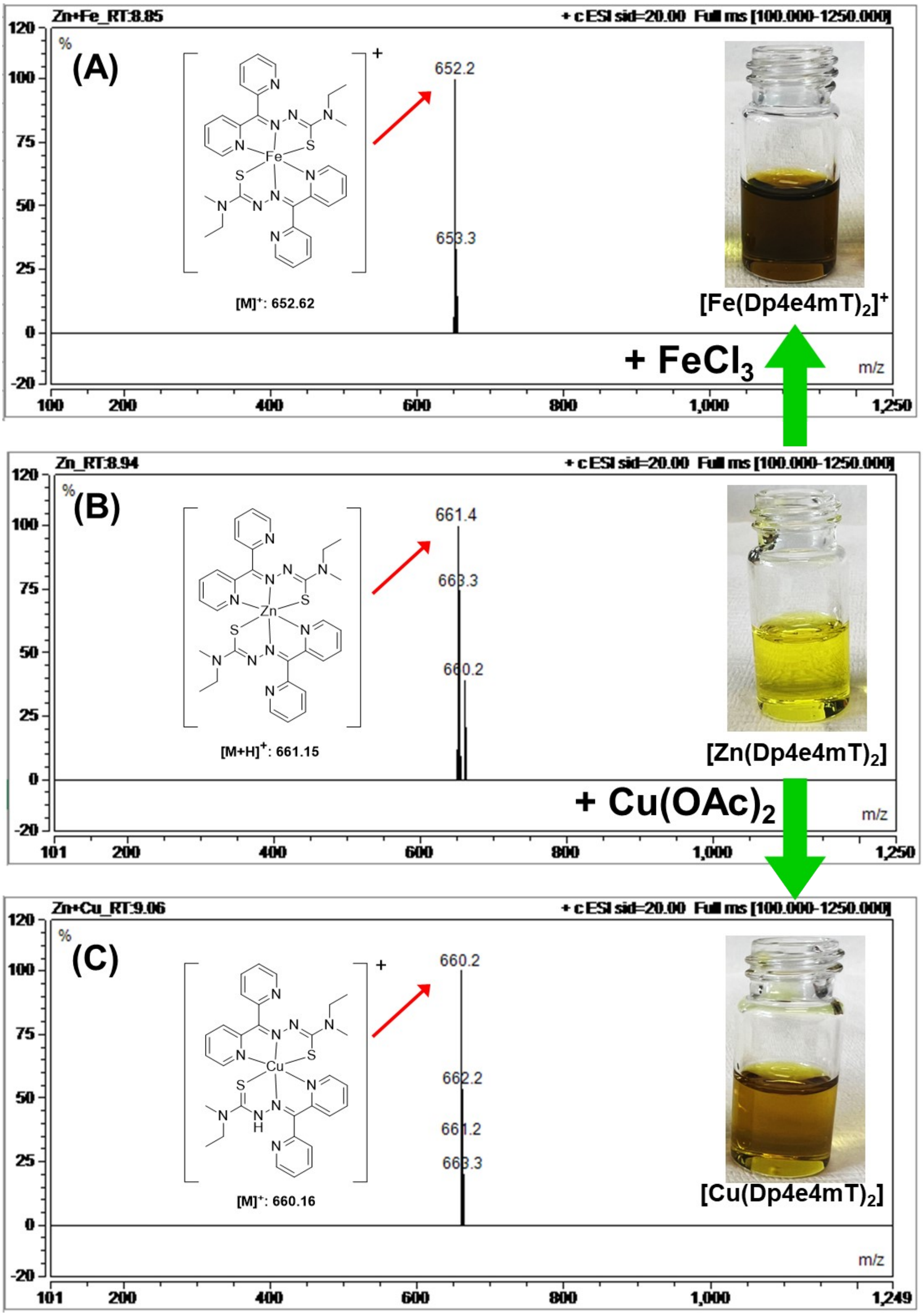

Figure S8. (A-C) Transmetallation of [Zn(Dp4e4mT)$_2$] was examined by LC-MS upon the addition of 1 equivalent of $FeCl_3$ or $Cu(OAc)_2$. A 1:1 ratio of [Zn(Dp4e4mT)$_2$] to metal salts ($FeCl_3$ or $Cu(OAc)_2$) was prepared in DMSO and incubated for 24 h/20 °C. LC-MS was then performed.

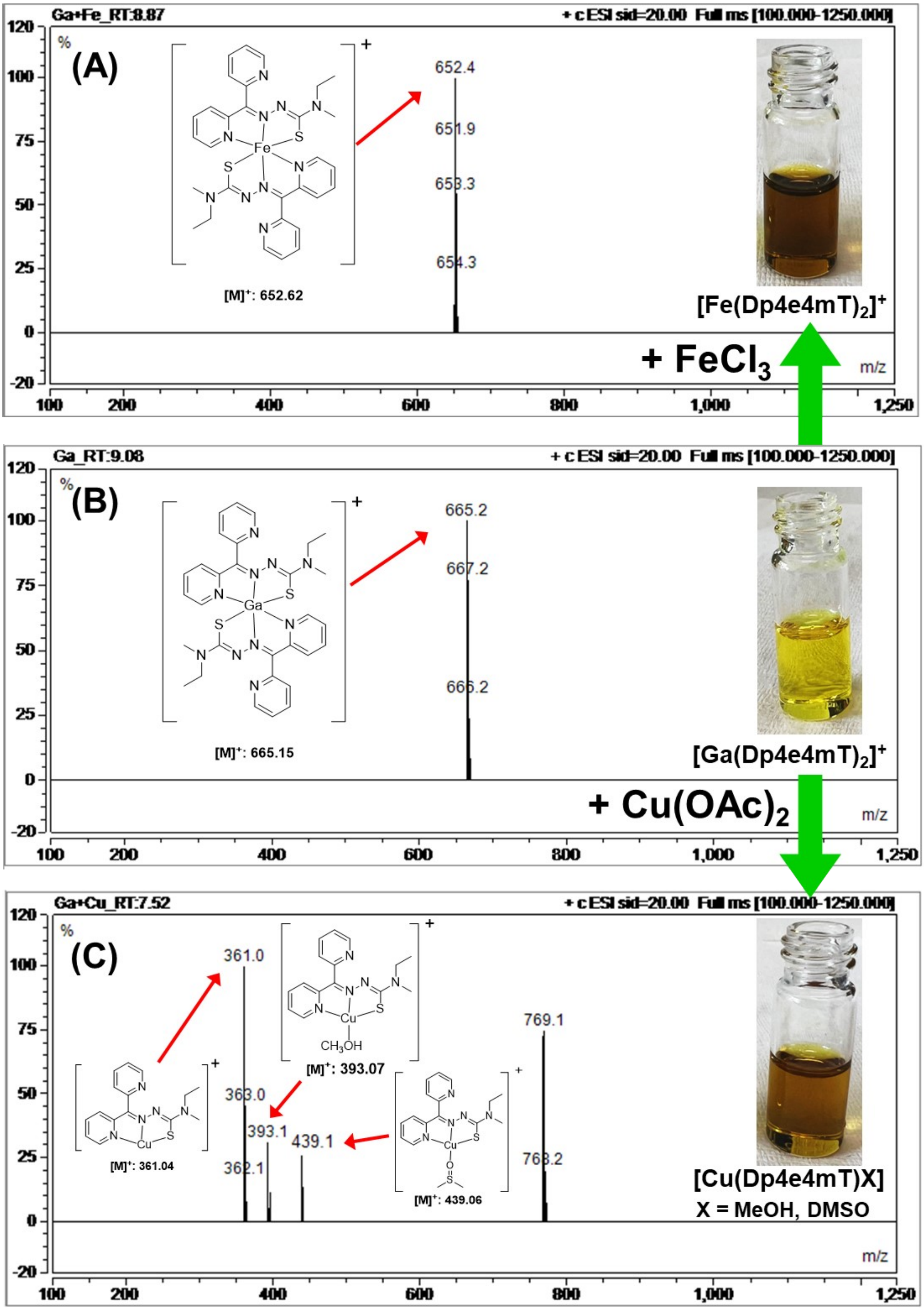

Figure S9. (A-C) Transmetallation of $[Ga(Dp4e4mT)_2]^+$ was examined by LC-MS upon the addition of 1 equivalent of $FeCl_3$ or $Cu(OAc)_2$. A 1:1 ratio of $[Ga(Dp4e4mT)_2]^+$ to metal salts ($FeCl_3$ or $Cu(OAc)_2$) was prepared in DMSO and incubated for 24 h/20 °C. LC-MS was then performed.

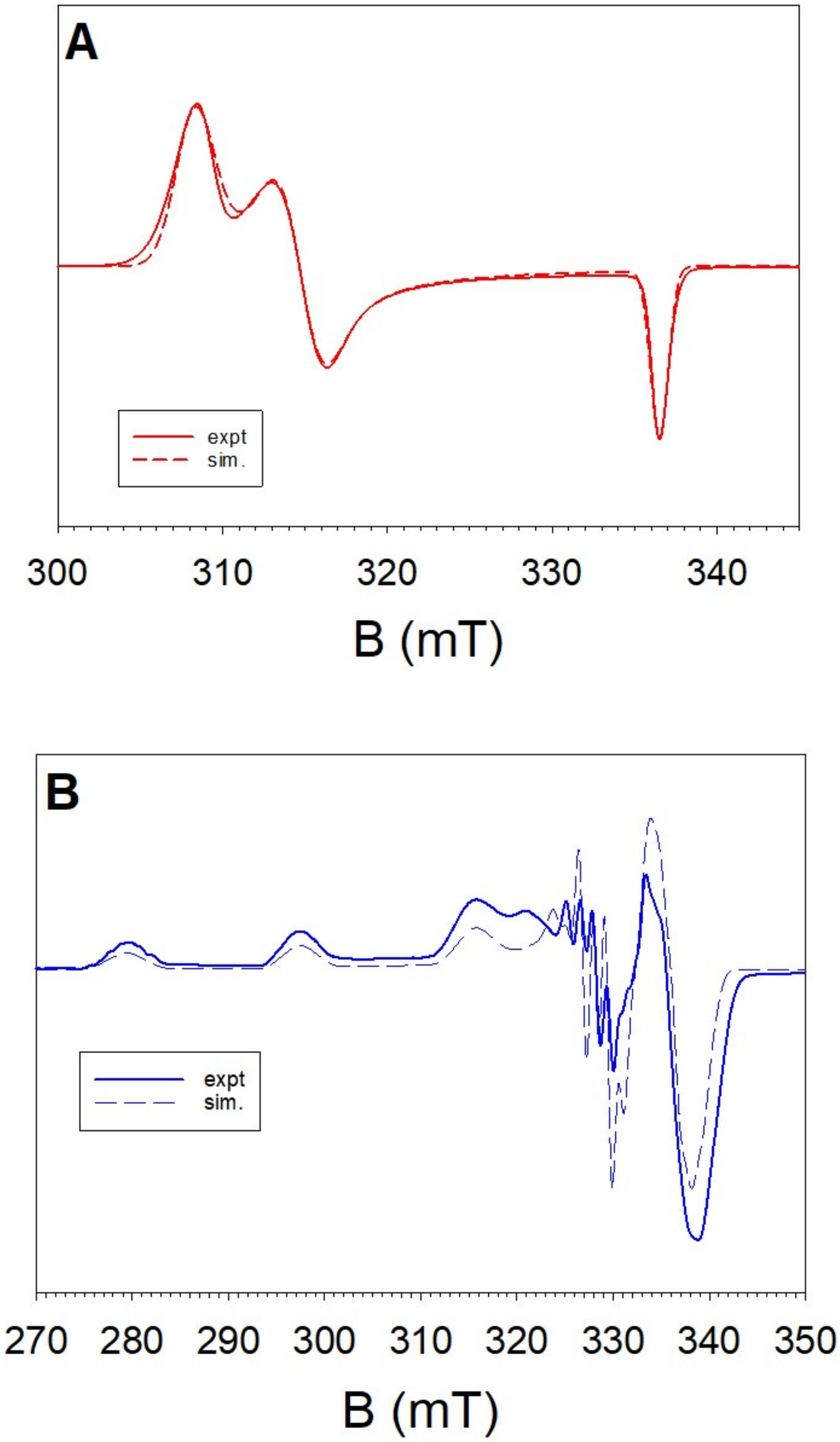

Figure S10. Experimental and simulated X-band EPR spectra of: **(A)** $[Fe(Dp4e4mT)_2]^+$; and **(B)** [Cu(Dp4e4mT)Cl] (500 µM in DMSO:$CHCl_3$ 1:1, 105 K).

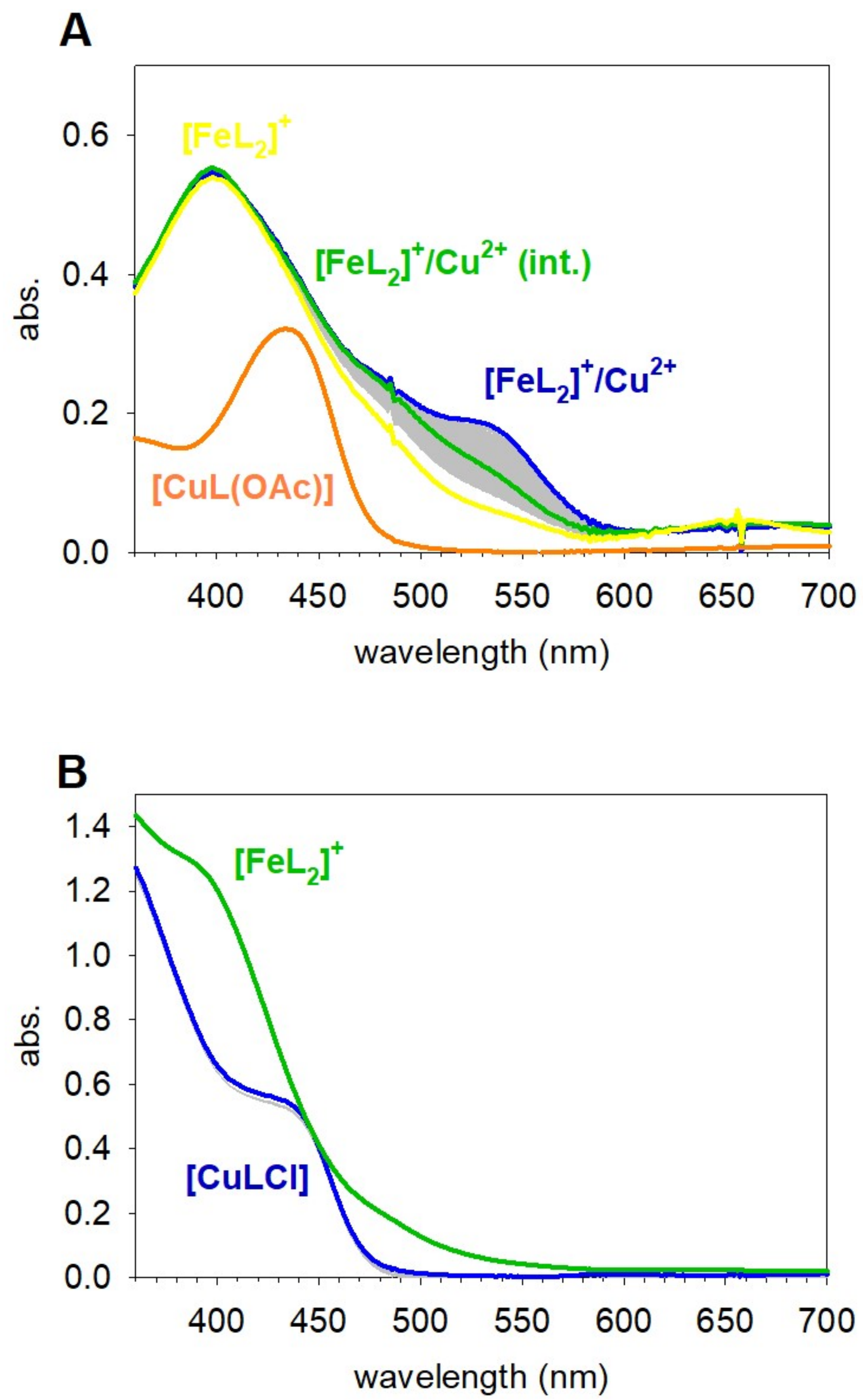

Figure S11. Time resolved UV-Vis spectra of: **(A)** $[Fe(Dp4e4mT)_2]^+$ (25 μM in DMSO) in reaction with $Cu(OAc)_2$ (250 μM) (13 s intervals over 100 min). The blue curve is the initial spectrum the green curve is the intermediate and the yellow curve is the final product and the same as the spectrum of $[Fe(Dp4e4mT)_2]^+$. The spectrum of [Cu(Dp4e4mT)(OAc)] is shown (green) for comparison but is not formed here; and **(B)** [Cu(Dp4e4mT)Cl] (25 μM in DMSO) in reaction with $FeCl_3$ (250 μM) (15 min intervals over 15 h). The spectrum of $[Fe(Dp4e4mT)_2]^+$ is shown (green) for comparison but is not formed here. The overlaid grey curves are the experimental data.

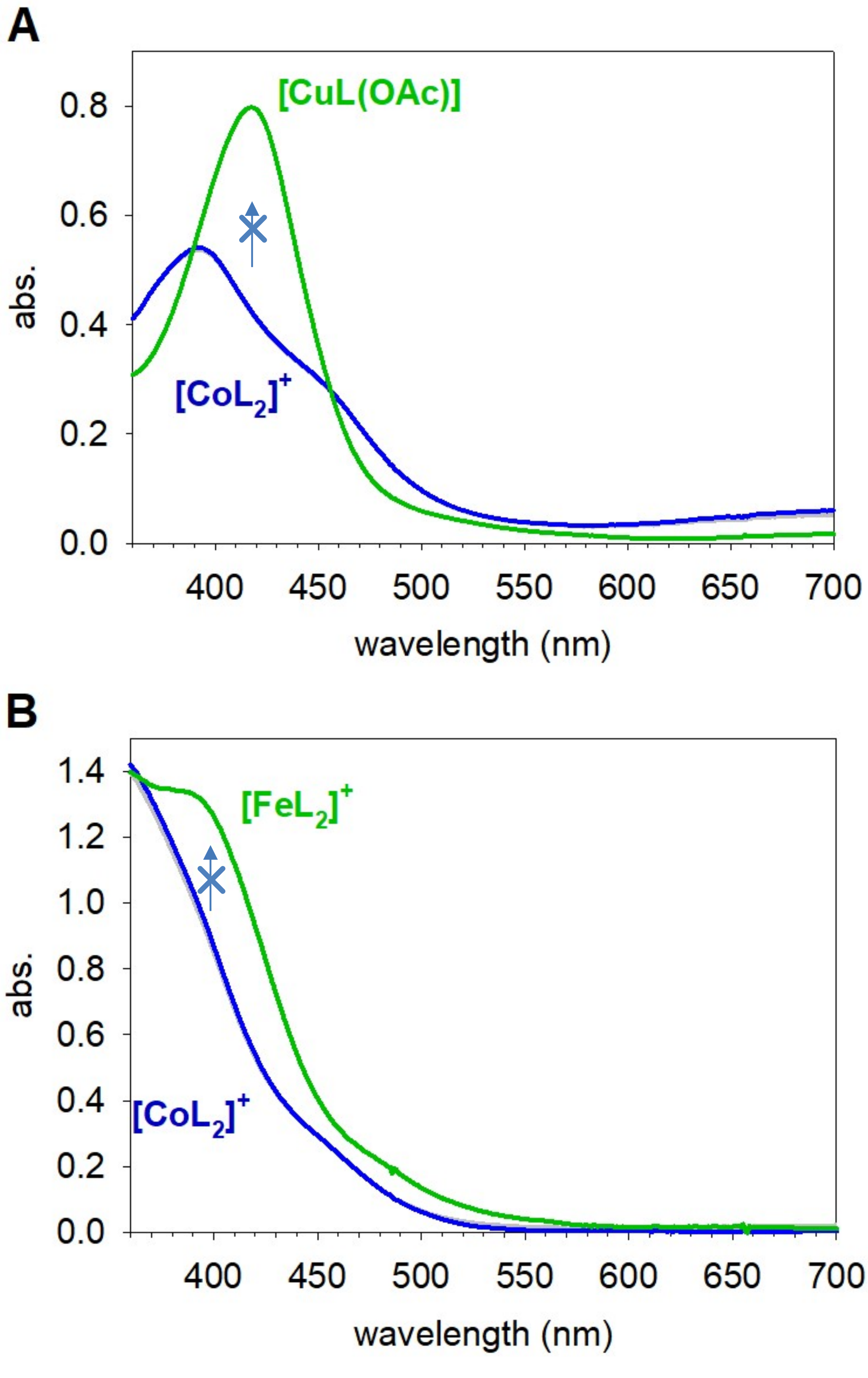

Figure S12. Time resolved UV-Vis spectra of $[Co(Dp4e4mT)_2]^+$ (25 μM in DMSO) in reaction with: **(A)** $Cu(OAc)_2$ (250 μM) (15 min intervals over 14 h) and **(B)** $FeCl_3$ (250 μM) (15 min intervals over 14 h). The blue curves show the Co(III) complex spectra prior to reaction and the green curves are the spectra of the possible products (not formed in either case), while the overlaid grey curves are the experimental data. Absorbance at ~360 nm in panel **(B)** is due to $FeCl_3$.

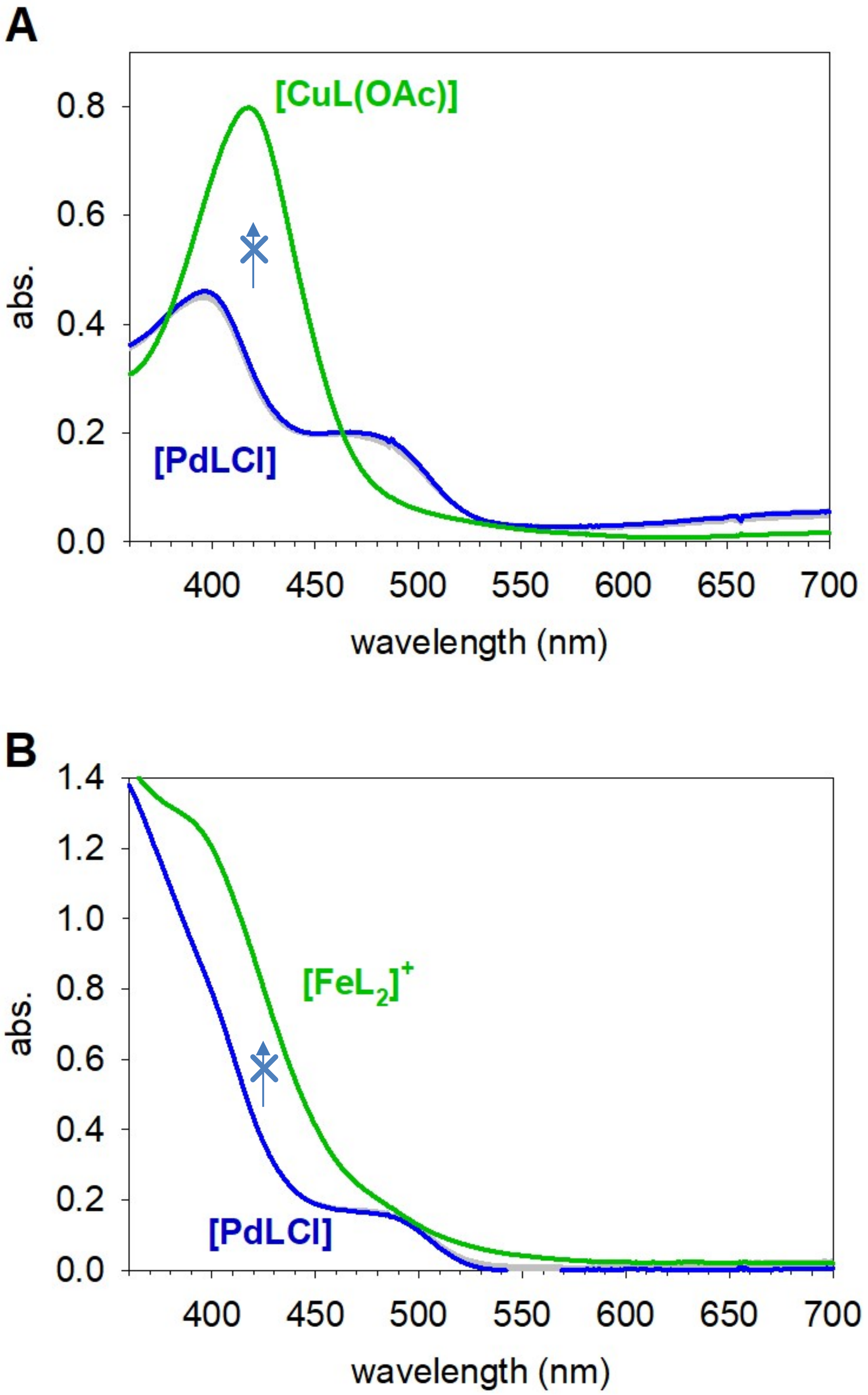

Figure S13. Time resolved UV-Vis spectra of [Pd(Dp4e4mT)Cl] (25 μM in DMSO) in reaction with: **(A)** $Cu(OAc)_2$ (250 μM) (15 min intervals over 14 h); and **(B)** $FeCl_3$ (250 μM) (15 min intervals over 14 h). The blue curves show the Pd(II) complex spectra prior to reaction and the green curves are the spectra of the possible products (not formed in either case), while the overlaid grey curves are the experimental data. Absorbance at ~360 nm in panel **(B)** is due to $FeCl_3$.

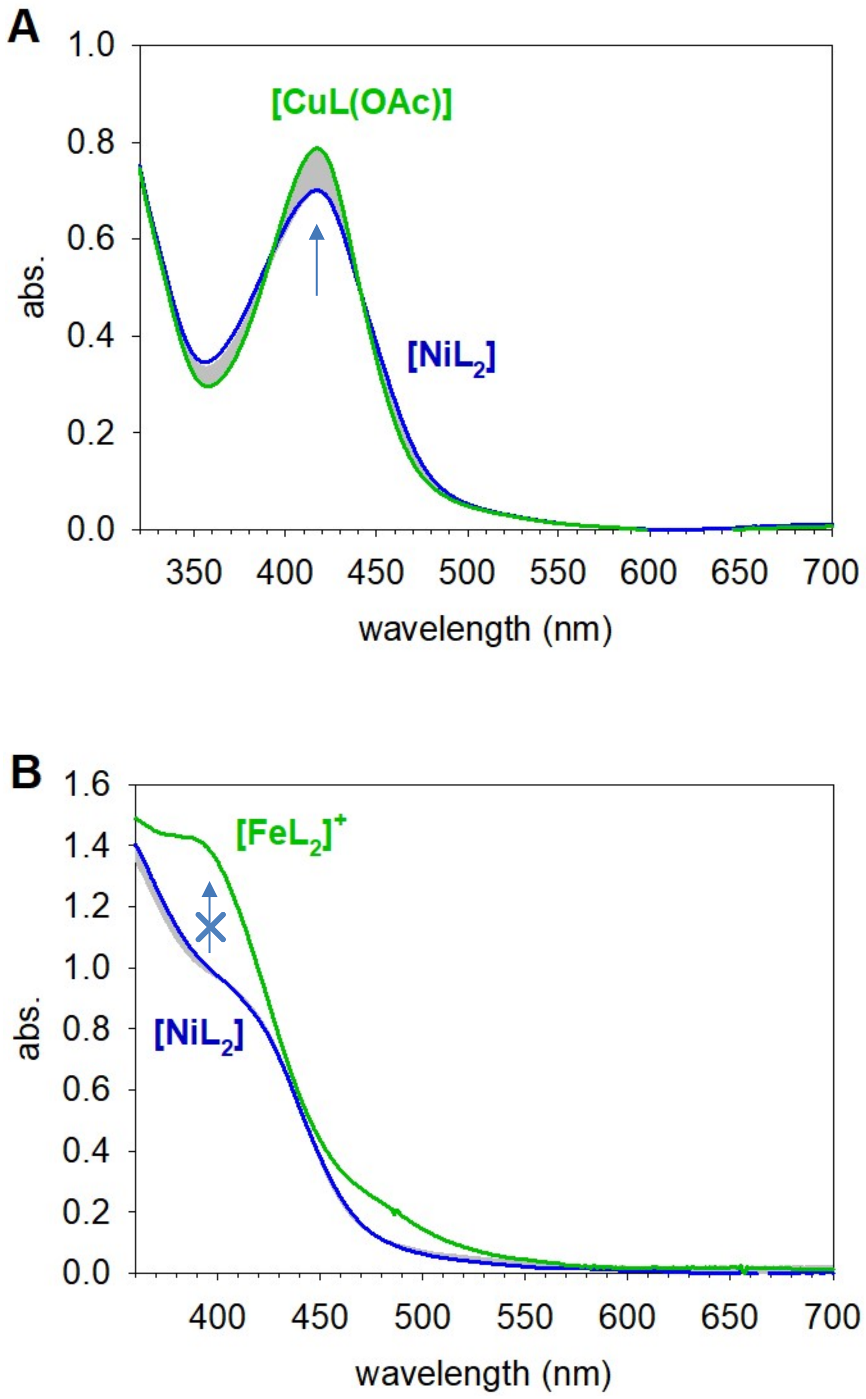

Figure S14. Time resolved UV-Vis spectra of [Ni(Dp4e4mT)$_2$] (25 μM in DMSO) in reaction with: **(A)** Cu(OAc)$_2$ (250 μM) (3 min intervals over 14 h); and **(B)** $FeCl_3$ (250 μM) (15 min intervals over 14 h). The blue curves show the Ni(II) complex spectra prior to reaction and the green curves are the spectra of: **(A)** the final product; or **(B)** the possible product (not formed), while the overlaid grey curves are the experimental data. Absorbance at ~360 nm in panel (**B)** is due to $FeCl_3$.

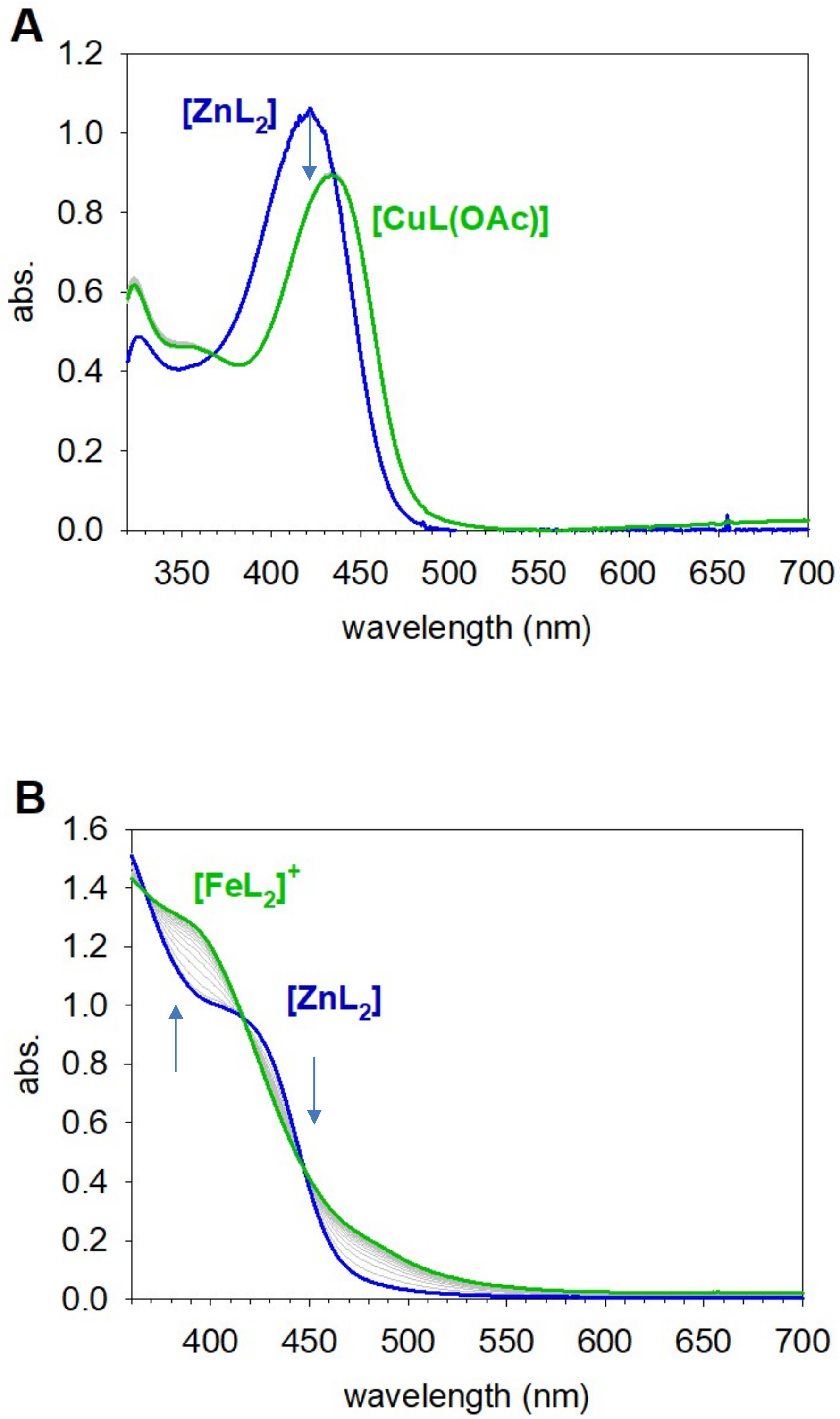

Figure S15. Time resolved UV-Vis spectra of [Zn(Dp4e4mT)$_2$] (25 µM in DMSO) in reaction with: **(A)** Cu(OAc)$_2$ (250 µM) (13 s intervals over 17 min); and **(B)** $FeCl_3$ (250 µM) (30 s intervals over 17 min). The blue curves show the Zn(II) complex spectra prior to reaction and the green curves are the spectra of the final products, while the overlaid grey curves are the experimental data. The reaction is immediate in panel **(A)**. Absorbance at ~360 nm in panel **(B)** is due to $FeCl_3$.

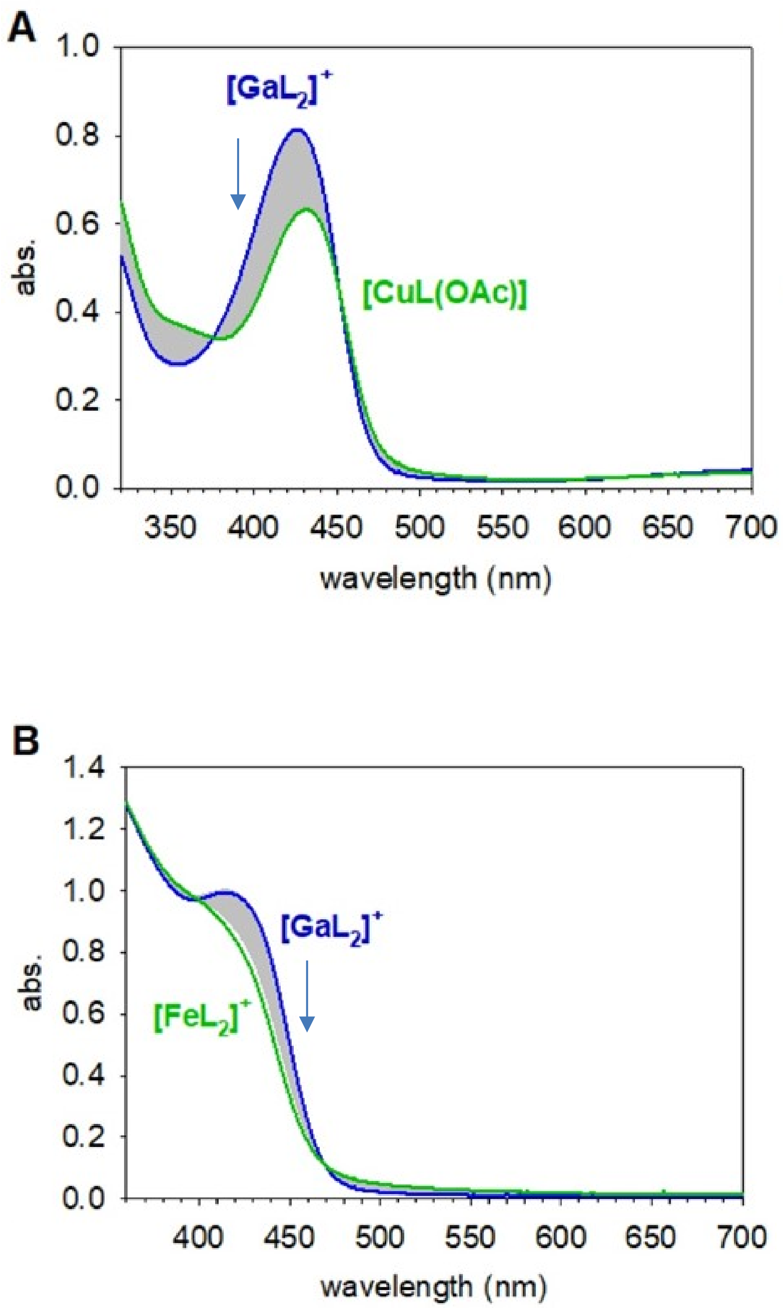

Figure S16. Time resolved UV-Vis spectra of $[Ga(Dp4e4mT)_2]^+$ (25 µM in DMSO) in reaction with: **(A)** $Cu(OAc)_2$ (250 µM) (3 min intervals over 14 h); and **(B)** $FeCl_3$ (250 µM) (3 min intervals over 14 h). The blue curves show the Ga(III) complex spectra prior to reaction and the green curves are the spectra of the final products, while the overlaid grey curves are the experimental data. Absorbance at ~360 nm in panel **(B)** is due to $FeCl_3$.

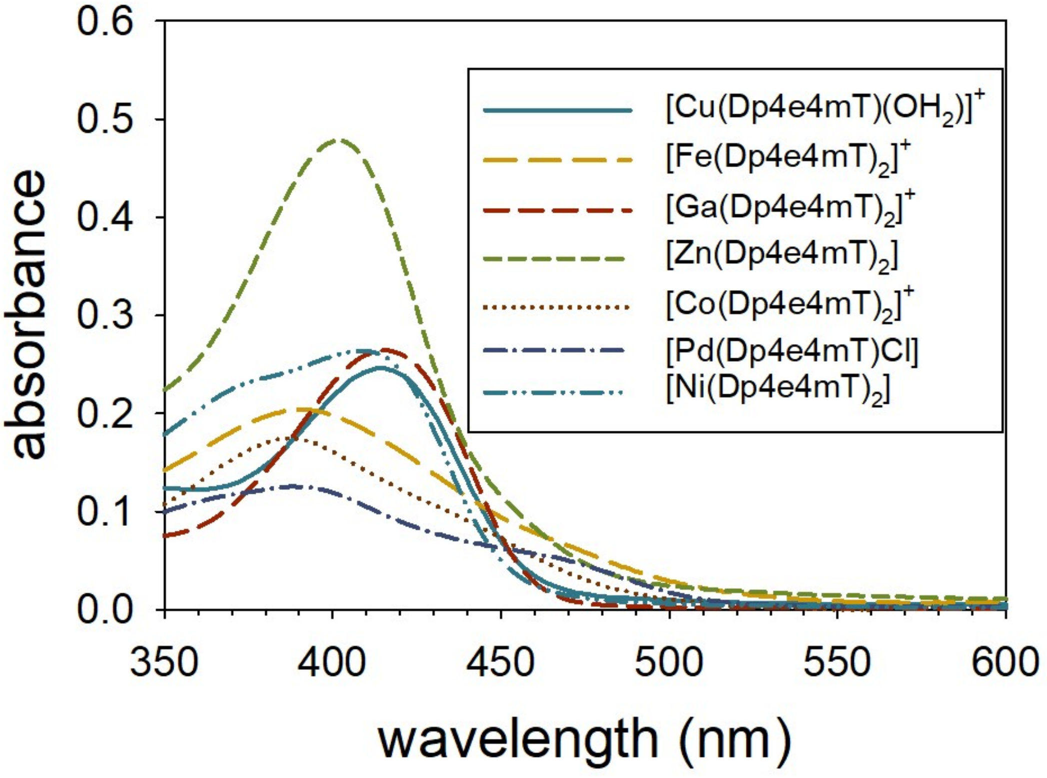

Figure S17. UV-Vis spectra (*ca*. 10 μM aqueous solutions at pH 7.4) of the Dp4e4mT complexes.

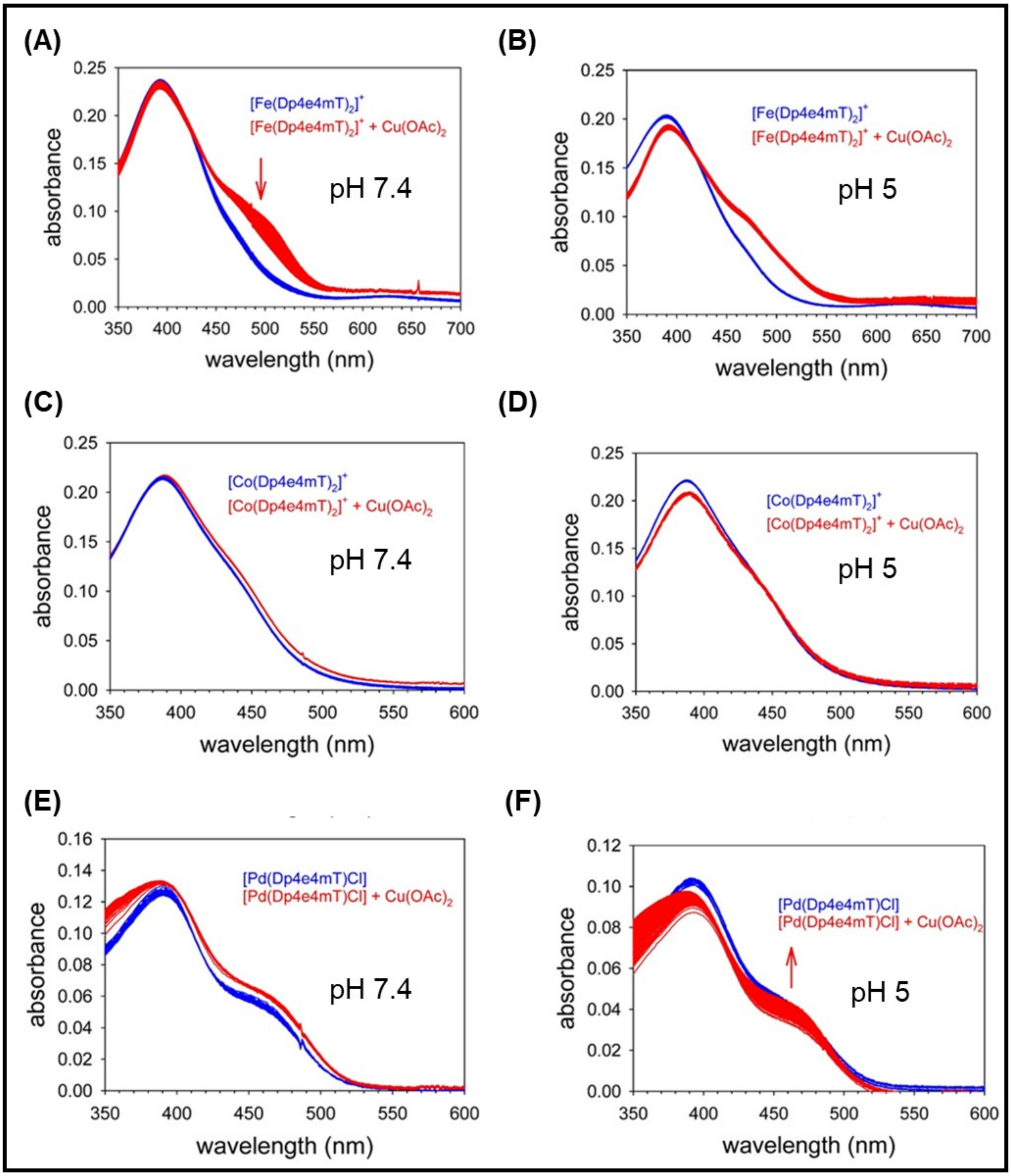

Figure S18. Time resolved UV-Vis spectra at 30 s intervals of the following complexes (*ca.* 10 µM) in the absence (blue) and presence (red) of $Cu(OAc)_2$ (100 µM): **(A)** $[Fe(Dp4e4mT)_2]^+$ at pH 7.4; **(B)** $[Fe(Dp4e4mT)_2]^+$ at pH 5.0; **(C)** $[Co(Dp4e4mT)_2]^+$ at pH 7.4; **(D)** $[Co(Dp4e4mT)_2]^+$ at pH 5.0; **(E)** [Pd(Dp4e4mT)Cl] at pH 7.4; **(F)** [Pd(Dp4e4mT)Cl] at pH 5.0.

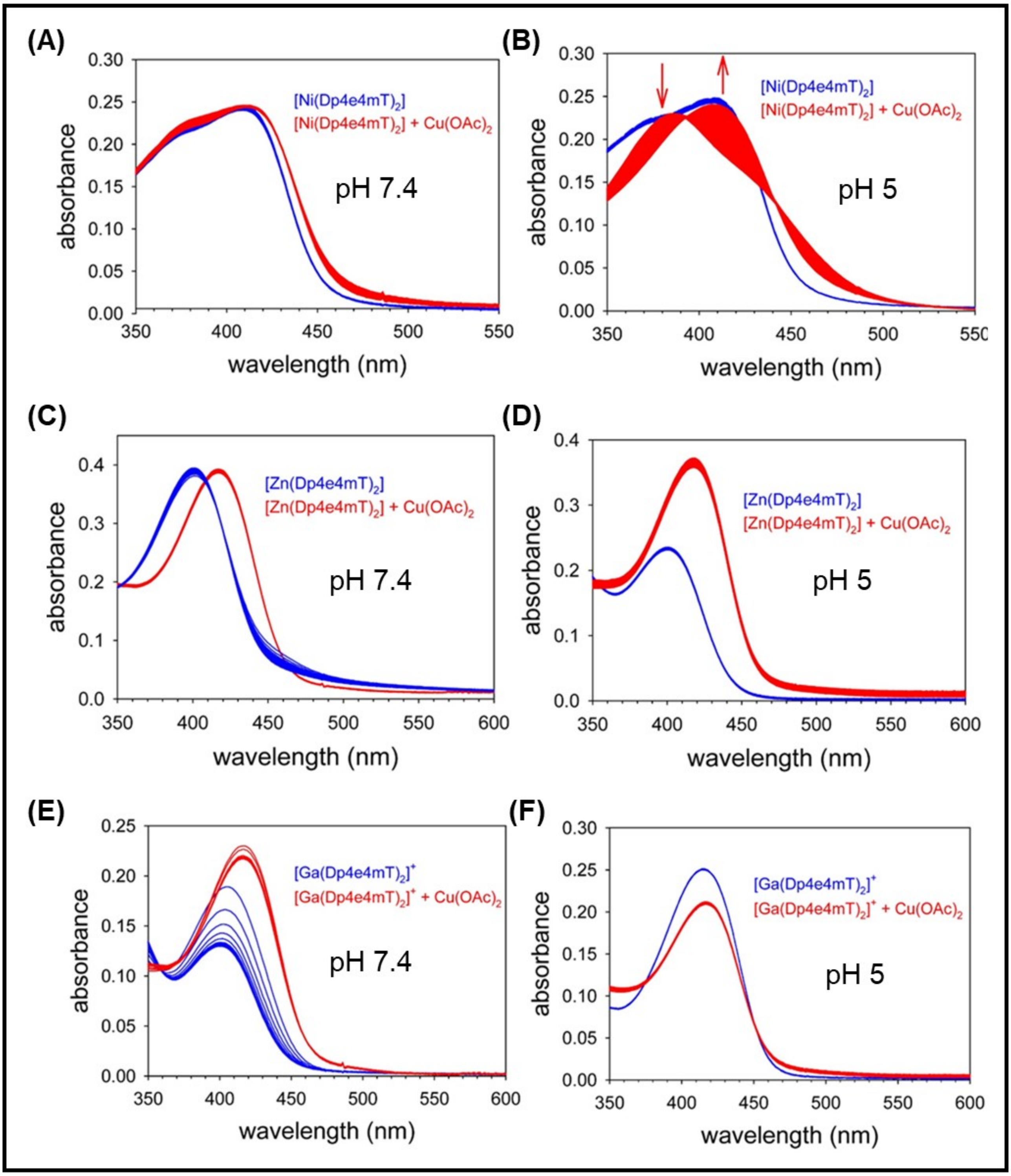

Figure S19. Time resolved UV-Vis spectra at 30 s intervals of the following complexes (*ca.* 10 µM) in the absence (blue) and presence (red) of $Cu(OAc)_2$ (100 µM): **(A)** $[Ni(Dp4e4mT)_2]$ at pH 7.4; **(B)** $[Ni(Dp4e4mT)_2]$ at pH 5.0; **(C)** $[Zn(Dp4e4mT)_2]$ at pH 7.4; **(D)** $[Zn(Dp4e4mT)_2]$ at pH 5.0; **(E)** $[Ga(Dp4e4mT)_2]^+$ at pH 7.4; **(F)** $[Ga(Dp4e4mT)_2]^+$ at pH 5.0.

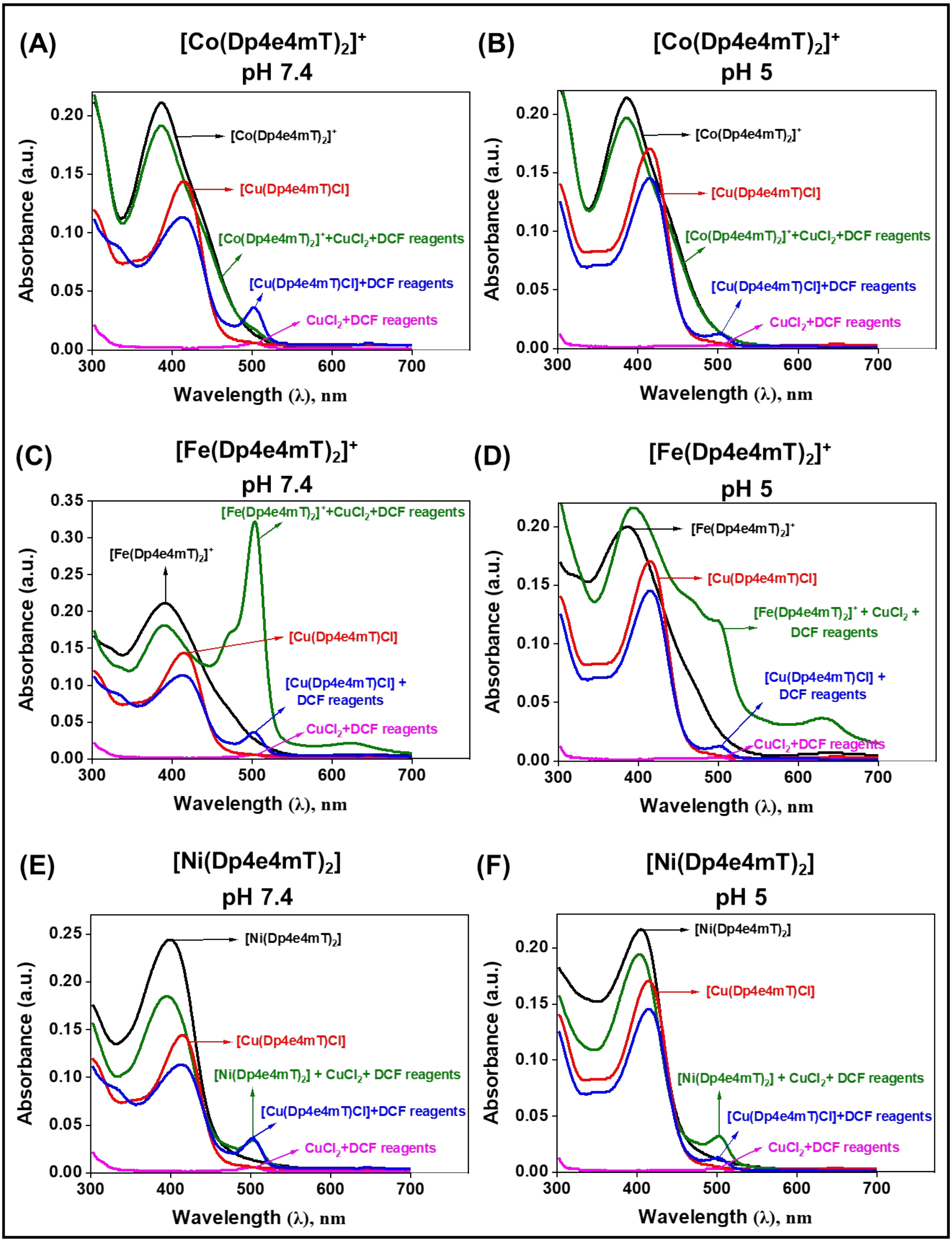

Figure S20. (A-F) The H_2DCF oxidizing activity of $[Co(Dp4e4mT)_2]^+$, $[Fe(Dp4e4mT)_2]^+$, and $[Ni(Dp4e4mT)_2]$ (10 μM) was measured by UV-Vis spectroscopy upon adding $CuCl_2$ and DCF reagents (*i.e.*, L-cysteine, H_2O_2, and H_2DCF) at both pH 7.4 and pH 5. Oxidation of H_2DCF leads to the production of the fluorescent 2′,7′-dichlorofluorescein (DCF), with an excitation at 503 nm.

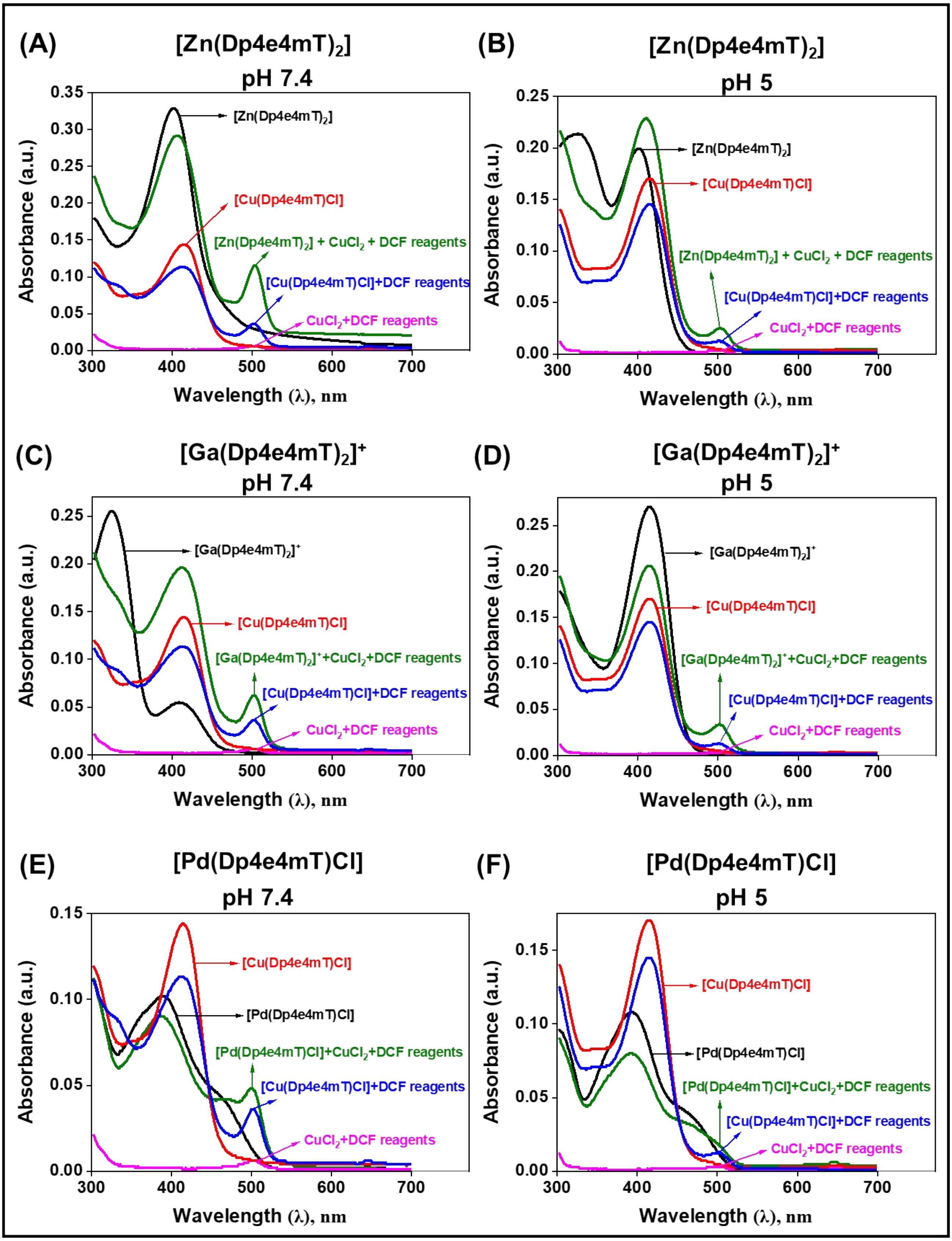

Figure S21. (A-F) The H_2DCF oxidizing activity of [Zn(Dp4e4mT)$_2$], [Ga(Dp4e4mT)$_2$]$^+$, and [Pd(Dp4e4mT)Cl] (10 μM) was measured by UV-Vis spectroscopy upon adding $CuCl_2$ and DCF reagents (*i.e.*, L-cysteine, H_2O_2, and H_2DCF) at both pH 7.4 and pH 5. Oxidation of H_2DCF leads to the production of the fluorescent 2′,7′-dichlorofluorescein (DCF), with an excitation at 503 nm.

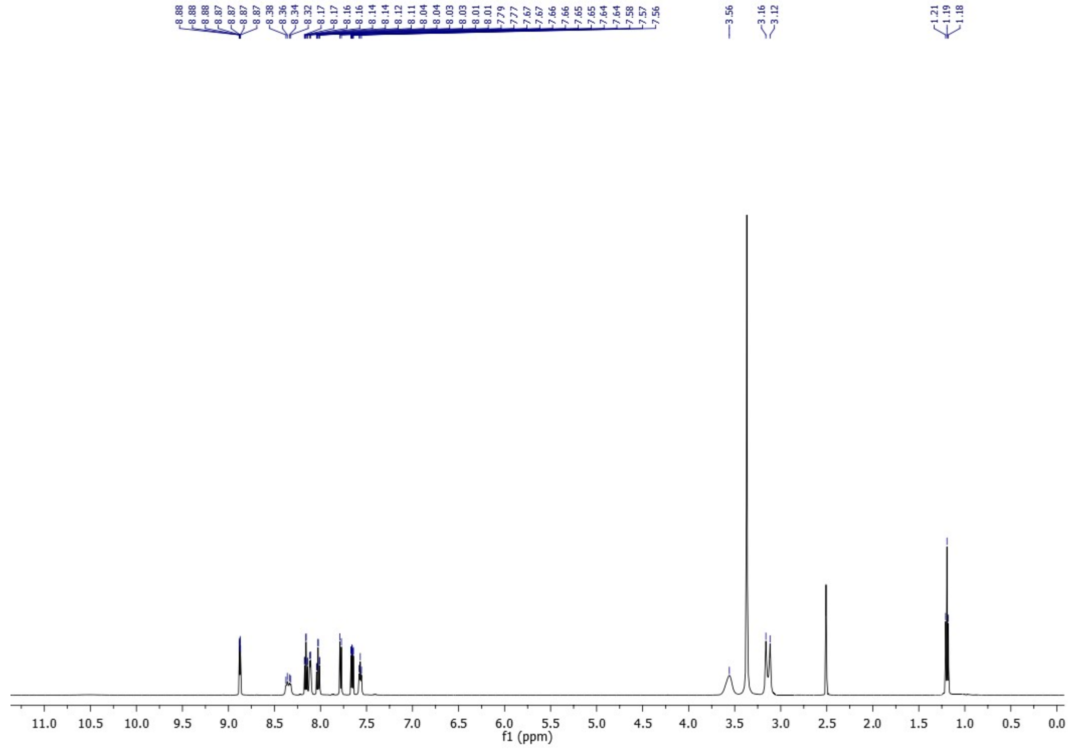

Figure S22. ^{1}H NMR spectrum of $[Co(Dp4e4mT)_2]^+$ in d_6-DMSO.

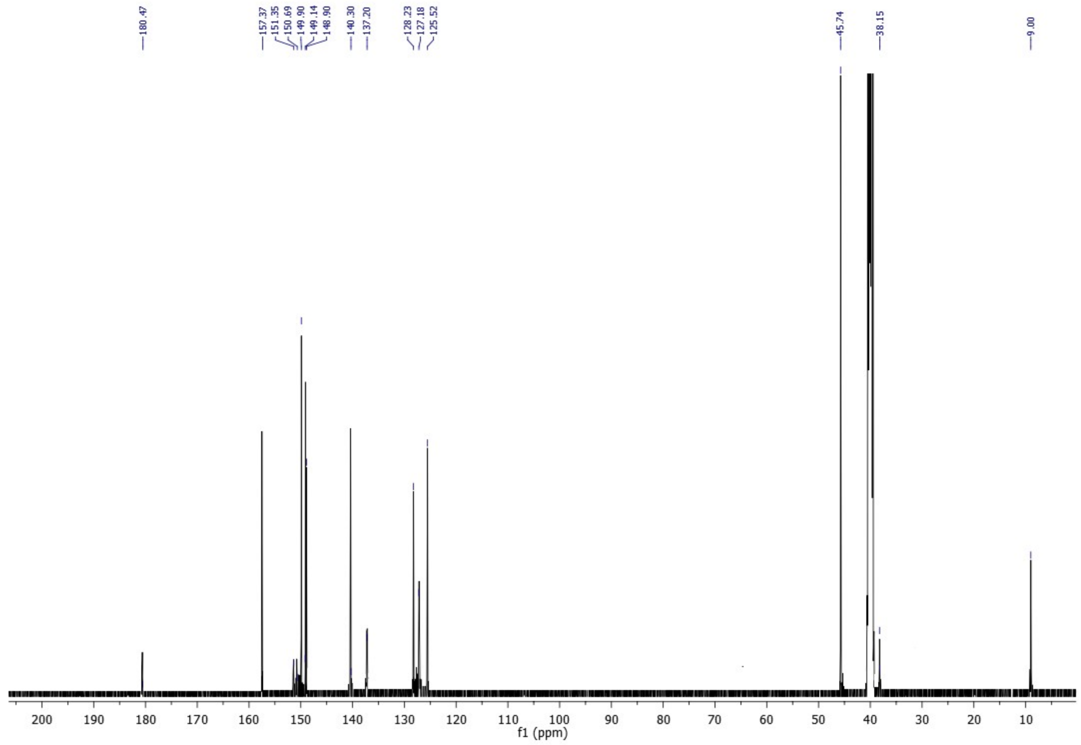

Figure S23. ^{13}C NMR spectrum of $[Co(Dp4e4mT)_2]^+$ in d_6-DMSO.

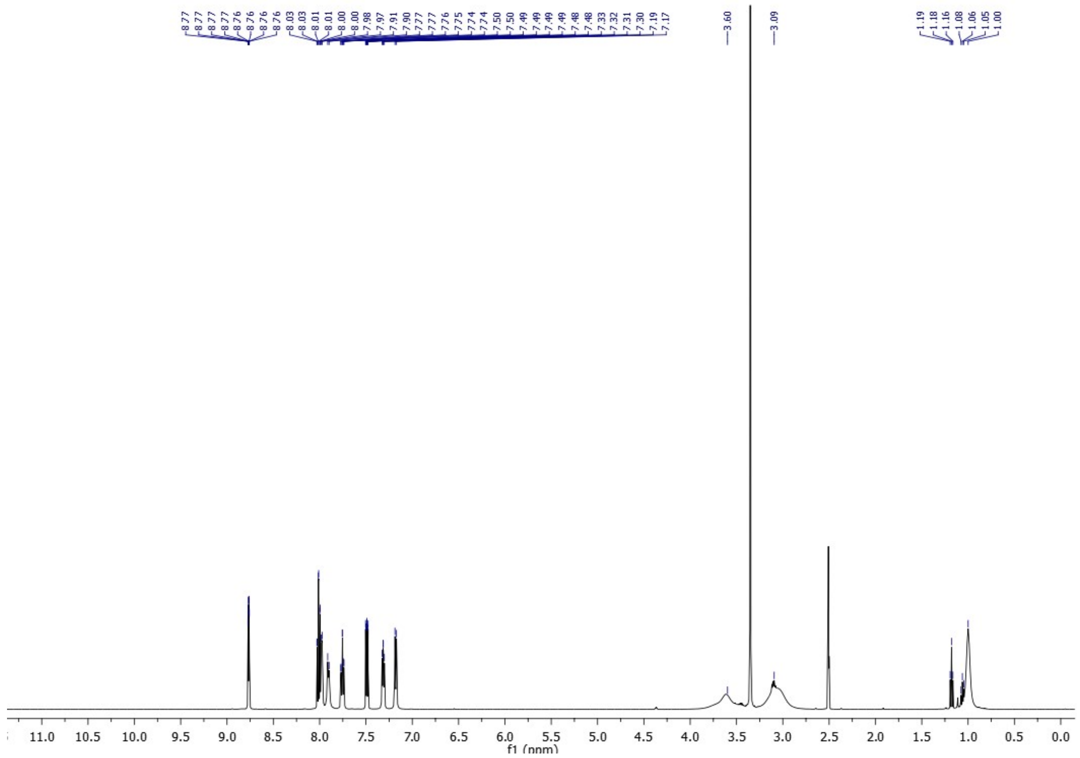

Figure S24. ^{1}H NMR spectrum of [Zn(Dp4e4mT)$_2$] in d_6-DMSO.

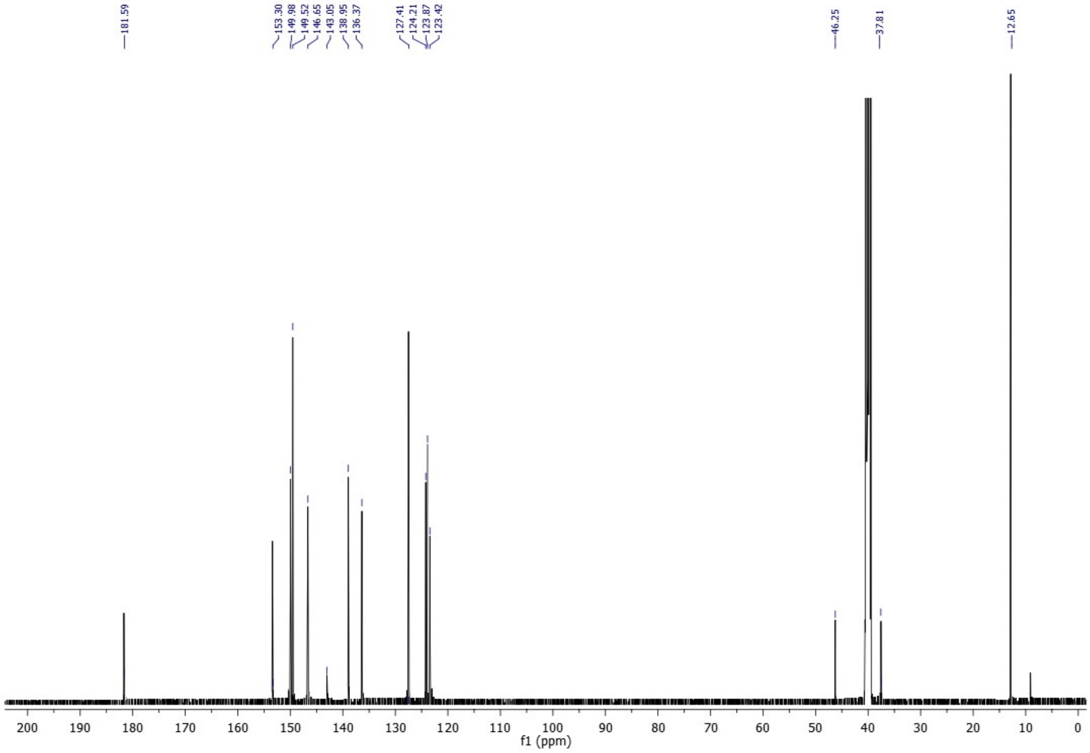

Figure S25. ^{13}C NMR spectrum of [Zn(Dp4e4mT)$_2$] in d_6-DMSO.

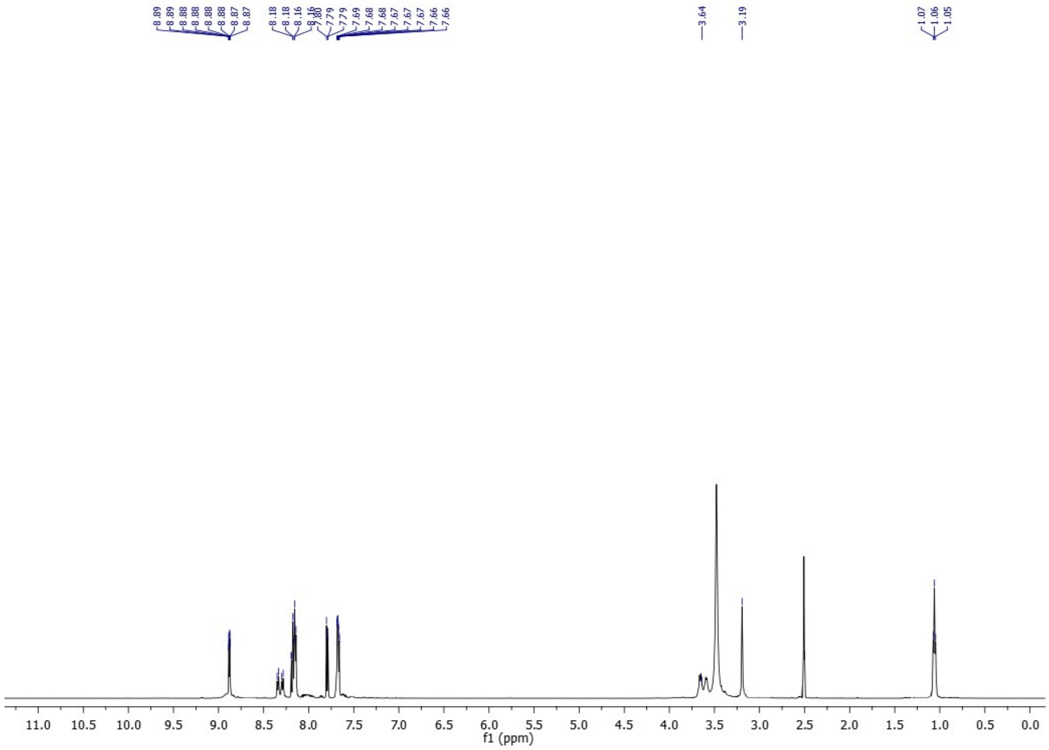

Figure S26. ^{1}H NMR spectrum of $[Ga(Dp4e4mT)_2]^+$ in d_6-DMSO.

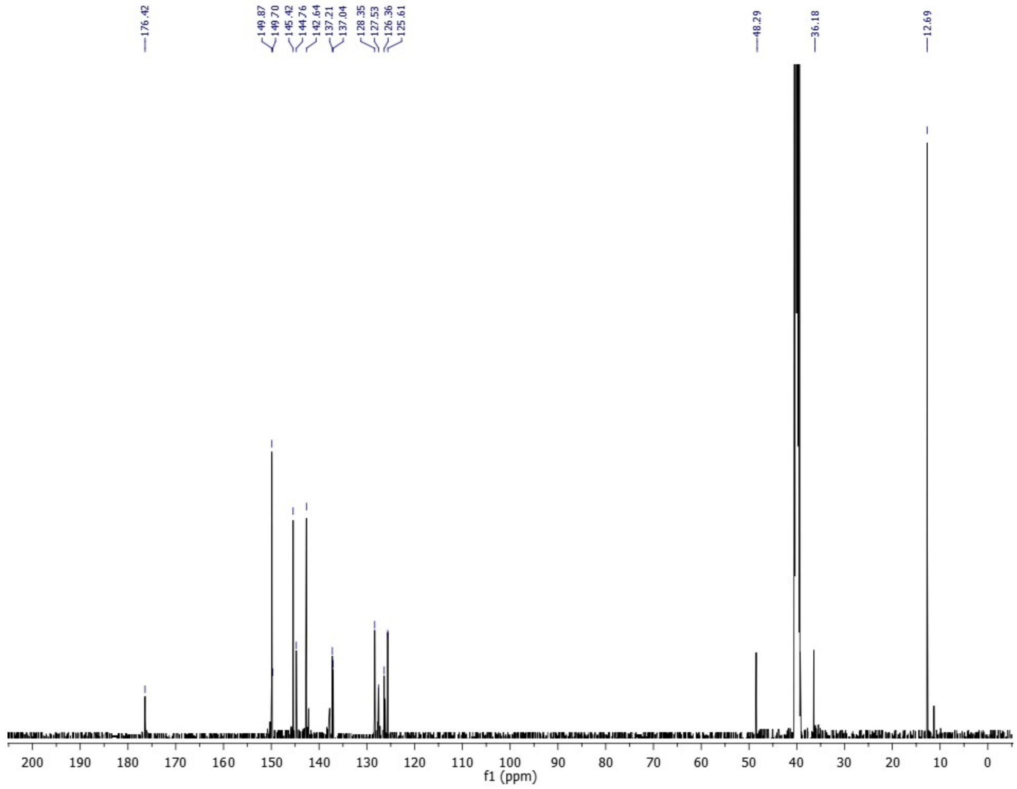

Figure S27. ^{13}C NMR spectrum of $[Ga(Dp4e4mT)_2]^+$ in d_6-DMSO.

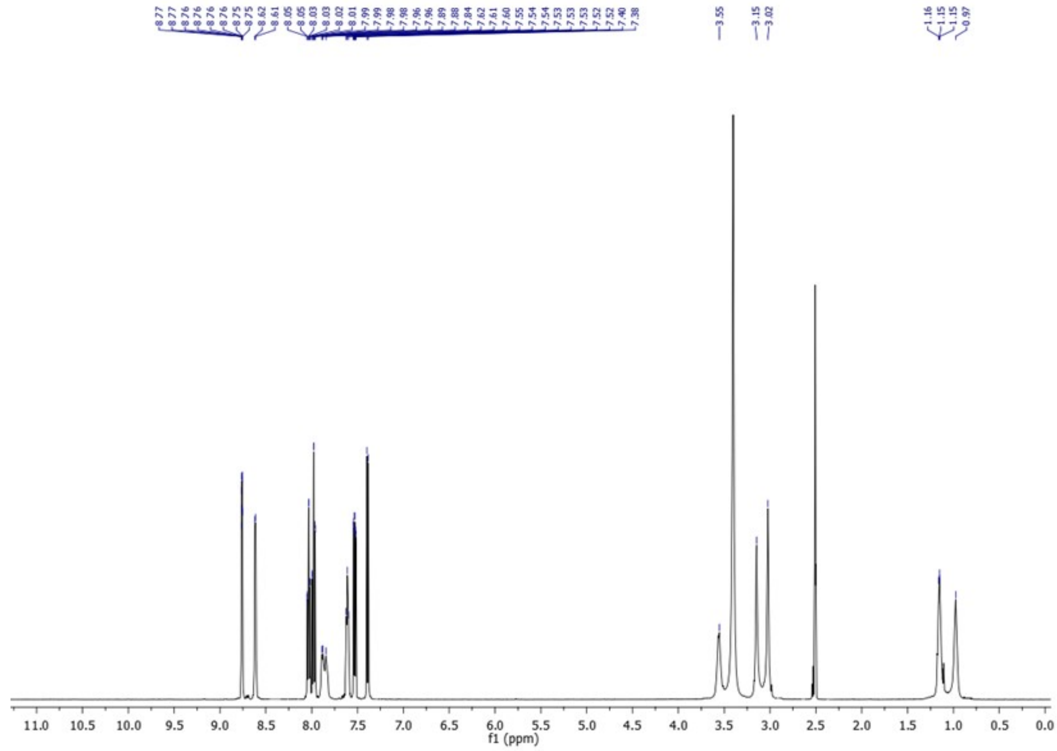

Figure S28. ^{1}H NMR spectrum of [Pd(Dp4e4mT)Cl] in d_6-DMSO.

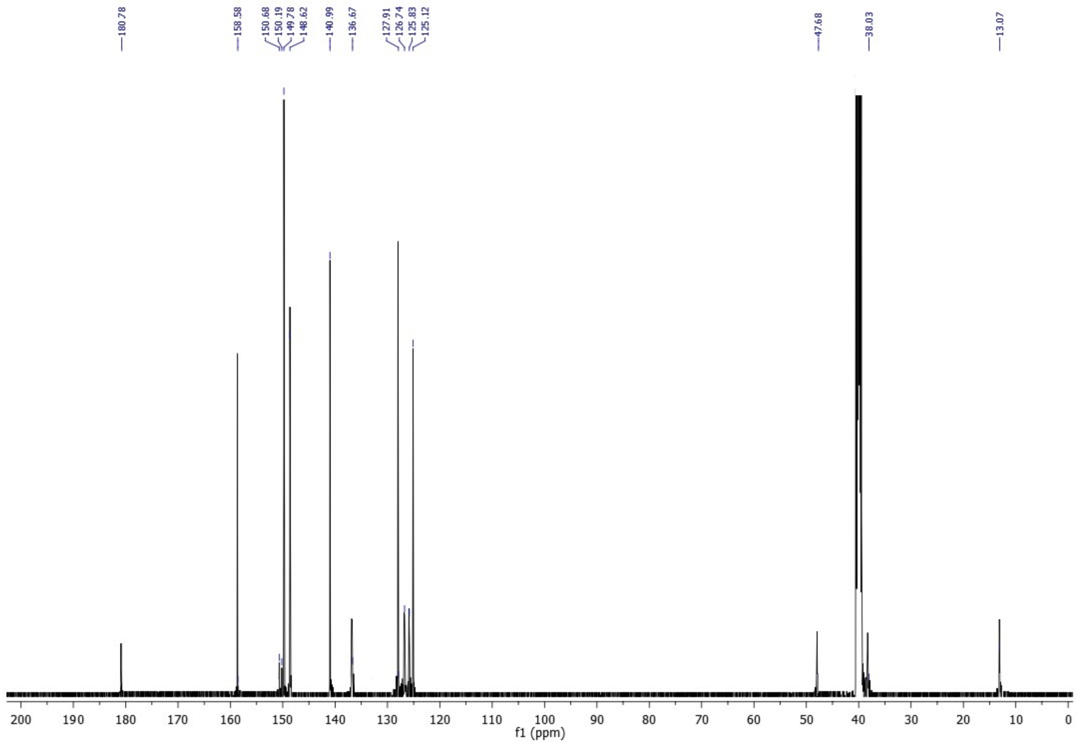

Figure S29. ^{13}C NMR spectrum of [Pd(Dp4e4mT)Cl] in d_6-DMSO.

References:

1. D. B. Lovejoy, D. M. Sharp, N. Seebacher, P. Obeidy, T. Prichard, C. Stefani, M. T. Basha, P. C. Sharpe, P. J. Jansson and D. S. Kalinowski, *J. Med. Chem.*, 2012, **55**, 7230-7244.

2. P. J. Jansson, P. C. Sharpe, P. V. Bernhardt and D. R. Richardson, *J. Med. Chem.*, 2010, **53**, 5759-5769.

3. D. R. Richardson, P. C. Sharpe, D. B. Lovejoy, D. Senaratne, D. S. Kalinowski, M. Islam and P. V. Bernhardt, *J. Med. Chem.*, 2006, **49**, 6510-6521.

4. A. E. Stacy, D. Palanimuthu, P. V. Bernhardt, D. S. Kalinowski, P. J. Jansson and D. R. Richardson, *J. Med. Chem.*, 2016, **59**, 8601-8620.

5. O. V. Dolomanov, L. J. Bourhis, R. J. Gildea, J. A. Howard and H. Puschmann, *J. Appl. Crystallogr.*, 2009, **42**, 339-341.

6. G. M. Sheldrick, *Acta Crystallogr. Sect. A: Found. Crystallogr.*, 2008, **64**, 112-122.

7. G. M. Sheldrick, *Acta Crystallogr. C Struct. Chem.*, 2015, **71**, 3-8.

8. M. Dharmasivam, B. Kaya, T. Wijesinghe, M. Gholam Azad, M. A. Gonzálvez, M. Hussaini, J. Chekmarev, P. V. Bernhardt and D. R. Richardson, *J. Med. Chem.*, 2023, **66**, 1426-1453.

9. S. Stoll and A. Schweiger, *J. Magn. Reson.*, 2006, **178**, 42-55.

10. M. Maeder and P. King, *ReactLab Kinetics; Jplus Consulting Pty Ltd: East Fremantle: WA, Australia*, 2009.

11. D. Richardson and E. Baker, *Biochim. Biophys. Acta - Mol. Cell Res.*, 1990, **1053**, 1-12.

12. D. R. Richardson, E. H. Tran and P. Ponka, *Blood*, 1995, **86**, 4295-4306.

13. A. E. Stacy, D. Palanimuthu, P. V. Bernhardt, D. S. Kalinowski, P. J. Jansson and D. R. Richardson, *J. Med. Chem.*, 2016, **59**, 4965-4984.

14. T. P. Wijesinghe, B. Kaya, M. A. Gonzalvez, J. R. Harmer, M. Gholam Azad, P. V. Bernhardt, M. Dharmasivam and D. R. Richardson, *J. Med. Chem.*, 2023, **66**, 15453-15476.

15. D. B. Lovejoy, P. J. Jansson, U. T. Brunk, J. Wong, P. Ponka and D. R. Richardson, *Cancer Res.*, 2011, **71**, 5871-5880.

16. M. J. Reiniers, R. F. van Golen, S. Bonnet, M. Broekgaarden, T. M. van Gulik, M. R. Egmond and M. Heger, *Anal. Chem.*, 2017, **89**, 3853-3857.

17. J. A. Bailey, *J. Chem. Educ.*, 2011, **88**, 995-998.
